# Coevolutionary analyses require phylogenetically deep alignments and better null models to accurately detect inter-protein contacts within and between species

**DOI:** 10.1186/s12859-015-0677-y

**Published:** 2015-08-25

**Authors:** Aram Avila-Herrera, Katherine S. Pollard

**Affiliations:** 1 Bioinformatics Graduate Program, University of California, San Francisco, USA; 2 Gladstone Institute of Cardiovascular Disease, University of California, San Francisco, USA; 3 Department of Epidemiology and Biostatistics, University of California, San Francisco, USA; 4 Institute for Human Genetics, University of California, San Francisco, 94158 CA USA

**Keywords:** Coevolution, Methods comparison, Inter-protein, Cross-species, Host-virus, Contact prediction, Protein interaction

## Abstract

**Background:**

When biomolecules physically interact, natural selection operates on them jointly. Contacting positions in protein and RNA structures exhibit correlated patterns of sequence evolution due to constraints imposed by the interaction, and molecular arms races can develop between interacting proteins in pathogens and their hosts. To evaluate how well methods developed to detect coevolving residues within proteins can be adapted for cross-species, inter-protein analysis, we used statistical criteria to quantify the performance of these methods in detecting inter-protein residues within 8 angstroms of each other in the co-crystal structures of 33 bacterial protein interactions. We also evaluated their performance for detecting known residues at the interface of a host-virus protein complex with a partially solved structure.

**Results:**

Our quantitative benchmarking showed that all coevolutionary methods clearly benefit from alignments with many sequences. Methods that aim to detect direct correlations generally outperform other approaches. However, faster mutual information based methods are occasionally competitive in small alignments and with relaxed false positive rates. Two commonly used null distributions are anti-conservative and have high false positive rates in some scenarios, although the empirical distribution of scores performs reasonably well with deep alignments.

**Conclusions:**

We conclude that coevolutionary analysis of cross-species protein interactions holds great promise but requires sequencing many more species pairs.

**Electronic supplementary material:**

The online version of this article (doi:10.1186/s12859-015-0677-y) contains supplementary material, which is available to authorized users.

## Background

Coevolution—“the change of a biological object triggered by the change of a related object” [1]—is a powerful concept when applied to molecular sequence analysis because it reveals positional relationships that are preserved across evolutionary time scales. Sequence evolution is constrained by essential molecular interactions, such as contacts within a protein or RNA structure, as well as inter-molecular interactions within protein complexes and signaling pathways. These constraints define an epistasis (i.e. genetic interaction) between sites (residues or base-pairs) where the probability of a substitution depends on the states of other sites involved in an interaction [2]. For example, a mildly deleterious or neutral mutation may change the fitness landscape such that compensatory or advantageous mutations at another site become more likely. Understanding the basic connections and dependencies between these molecular machines is invaluable in learning how cells function, adapt, and how they can be manipulated into performing new tasks or correcting harmful behaviors, as in disease for example.

Because epistasis can induce correlation between substitution patterns among columns in multiple sequence alignments, many methods have been developed that use evidence of coevolving alignment columns to detect physical interactions within and between biomolecules. These methods draw inspiration from diverse techniques in molecular phylogenetics, inverse statistical mechanics, Bayesian graphical modeling, information theory, sparse inference, and spectral theory (reviewed in [3, 4]).

Despite good rationale for coevolutionary approaches, physically interacting alignment columns have been notoriously difficult to identify from correlated patterns of sequence evolution for several reasons. First, shared evolutionary history creates a background of correlated substitution patterns against which it can be difficult to distinguish additional constraints derived from physical interactions. Common phylogeny is particularly strong within a gene family (e.g. predicting intra-molecular contacts). But it is also present across gene families within a species or even between species (e.g. predicting host-virus protein interactions), especially at shorter evolutionary distances where gene trees mirror species trees more closely. Coevolution methods have used a variety of approaches to counter the dependence induced by shared phylogeny, including removing closely related sequences from alignments to reduce non-independence [5, 6], differential weighting of sequences when computing statistics [7–9], and null distributions that directly model or indirectly account for phylogeny [10–13].

A second challenge arises when trying to distinguish correlated evolution that arises from direct versus indirect interactions. Alignment columns that are indirectly implicated in an interaction can be strongly correlated, and most columns are involved in multiple, partially overlapping interactions. For these reasons, close physical interactions may not produce patterns of substitution that are significantly more highly correlated than the background present in structures. This problem has been the focus of a recent class of coevolution methods that focuses on reducing the number of incorrect predictions by disentangling direct from indirect correlations [9, 14–17]. An alternative point of view considers these networks of indirectly correlated residues as protein sectors that can easily, through cooperative substitutions, respond to fluctuating evolutionary pressures [18]. Proteins are in fact quite dynamic, and many *unstructured* proteins are known to have important interactions [19, 20]. Coevolution methods have the exciting potential to reveal these hard to identify interactions, however distinguishing spurious correlations from true non-structural interactions remains a challenge.

The main barrier to overcoming this challenge is the impressively difficult task of compiling “gold standard” data sets in which true coevolving sites are clearly defined. Structural and systems biology have had great success in identifying and characterizing many important interactions (e.g. Nucleosome [21], Proteasome [22], regulation in protein networks [23, 24]). However, resolving large complexes and unstructured proteins remains technically difficult, a daunting task as the number of proteins is ever increasing.

Finally, due to low power—resulting in part from the previous two challenges—physically interacting sites can typically only be detected in multiple sequence alignments that span large evolutionary divergences and contain many hundreds to thousands of sequences. Recent evaluations of a number of coevolution methods concluded that accurate contact predictions require alignments with one to five times as many sequences (with < 90 % sequence redundancy) as positions [25, 26]. Even in the current data rich era of computational biology, such deep alignments are difficult to obtain, especially for cross-species protein interactions (e.g. host and pathogen interactions) because both members of the interaction must be equally deeply sequenced. Additionally, resolving orthologs and paralogs is not trivial.

Despite these challenges, coevolutionary prediction of physically interacting alignment columns has been applied with success to intra-molecular contacts [7, 27–29] and well-characterized inter-molecular interactions [30], such as bacterial two-component signaling systems [31], enzyme complexes [32], and fertilization proteins [33]. Although the signal-to-noise ratio is too low and the search space too large to use sequence evolution to effectively identify pairs of physically interacting protein residues across entire proteomes; most pairs of sites with correlated substitution patterns are not in direct contact, and most physically interacting sites do not have statistically correlated substitution patterns [34].

However, the ability to now measure physical interactions between biomolecules with high-throughput technologies, such as affinity purification followed by mass spectrometry (APMS) [35], two-hybrid methods [36, 37], and protein complementation assays [38], raises the possibility of using sequence coevolution to refine predicted interactions in an experimentally reduced search space. For example, correlated substitution patterns in pairs of proteins could help determine if an experimentally measured interaction is likely to represent direct physical contact versus an indirect interaction in a complex or a false positive. Coevolutionary analysis could also be informative regarding which of the sites in a pair of interacting molecules are most likely to be in physical contact.

One particularly exciting application of this approach is to characterize and potentially manipulate interacting residues in host-virus and host-parasite protein interactomes [23, 39]. Newly emerging data on antibody and antigen sequences within a host [40] offers an opportunity to harness coevolutionary signals to investigate the mechanisms of broadly neutralizing antibodies and immune evasion. The primary open question for these new applications is whether existing methods are sensitive and specific enough to detect coevolution with the levels of constraint and divergence that are present in inter-molecular data sets of modest size.

To this end, we designed data processing scripts, statistical evaluation and visualization tools, and simulation pipelines that allowed us to easily extend a suite of coevolution methods designed for intra-protein interaction prediction (Table 1) so that they can be used to test for patterns of correlated sequence evolution at pairs of sites in two different proteins, potentially from different sets of organisms in different parts of the tree of life (e.g. human-bacteria, bacteria-phage interactions). We then applied this integrated framework for coevolutionary analysis to refine and annotate a recently derived human-HIV1 protein-protein interaction network [23] and to test for coevolution in the well studied arms-race interaction between the mammalian cytidine deaminase APOBEC3G (A3G) and its HIV1 antagonist, Vif. Because fewer than ten orthologous mammal-lentivirus proteome pairs have been sequenced and mammalian divergence is low, we hypothesized that power would be low in these settings.

**Table 1:** List of methods benchmarked

	Method	APC	Re-weighting	Reference	Software package
Information-based	MI	No	None	[8, 71]	infCalc
	VI			[65]	
	MI_j_			[8]	
	MI_Hmin_				
	MI_w_		seq %id	[9]	DCA
Direct	DI	Yes	seq %id, pseudocount		
	DI_256_			[68]	Code S1 in [68]
	DI_32_				
	DI_plm_		seq %id	[72]	plmDCA
	PSICOV		Blosum, pseudocount	[14]	PSICOV
Phylogenetic	CMP_cor_	No	Downsampling	[10]	CoMap
	CMP_chg_			[2]	
	CMP_vol_				
	CMP_pol_				

Coevolution methods benchmarked fall into three categories. Information-based methods: MI: mutual information [71], VI: variation of information [65], MI_j_: MI divided by alignment column-pair entropy, MI_Hmin_: MI divided by minimum column entropy [8], MI_w_: MI with adjusted amino acid probabilities. Direct methods: DI: direct information—MI with re-estimated joint probabilities [9], DI_256_, DI_32_: DI using Hopfield-Potts for dimensional reduction (256 and 32 patterns respectively) [68], DI_plm_: Frobenius norm of coupling matrices in 21-state Potts model using pseudolikelihood maximization [72], PSICOV: sparse inverse covariance estimation [14]. Phylogenetic methods: CoMap *P*-values for four analyses CMP_cor_: substitution correlation analysis [10], CMP_pol_ for polarity compensation, CMP_chg_ for charge compensation, CMP_vol_ for volume compensation [2]

To quantify the limitations of coevolutionary methods when only a handful of sequences are available, we used a data set of 33 within-species bacterial protein-protein interactions. To systematically determine the parameters that affect performance, we focused on the well-characterized interaction between bacterial histidine kinase A (HisKA) and its response regulator (RR), for which a co-crystal structure and thousands of sequences are available. By sub-sampling HisKA-RR sequence pairs, we show that most methods have appreciable precision or power at low false positive rates for alignments with ∼500 or more sequences. However, the best performing method for a particular analysis will depend on whether power or precision is more important, the number of non-redundant sequences in the alignment, and whether the goal is to find structurally or functionally linked residues (i.e. long range interactions). By expanding this analysis to 32 additional bacterial interactions [30], we showed that these trends generalize beyond the specific example of HiskA and RR. We conclude that coevolution methods are able to identify some residues important for cross-species protein-protein interactions, but this approach will benefit greatly from additional sequence data.

## Results

### Performance benchmarking of coevolution methods

The coevolutionary methods benchmarked in our analyses fall into three general groups (Table 1). Information-based methods are various flavors of mutual information (MI) between pairs of sites, each considered independently. Direct methods are those that consider pairs of sites in the context of a sparse global statistical model for contacts in the multiple sequence alignment. Phylogenetic methods explicitly use a substitution rate matrix and phylogenetic tree in their calculation of a coevolution statistic. The phylogenetic tree is used to account for the relatedness of the sequences—the observed sequences are themselves correlated due to their shared evolutionary histories. The substitution rate matrix may take into account the biochemical and physical properties of amino acid residues. The main phylogenetic method we report on, CoMap, reports a *P*-value based on internal simulation of independently evolving sites. In this benchmark we use this *P*-value as a statistic for comparison with other coevolution methods. Other differences among the coevolution methods include the incorporation of two additional techniques that have been shown to improve performance, re-weighting sequences such that similar sequences contribute less to the final score [5] and applying an Average Product Correction (APC) to remove background noise and phylogenetic signal from “raw” coevolution statistics [8].

To benchmark coevolution methods, we used 33 within-species pairs of proteins with co-crystal structures determined from *E. coli* proteins. These include a set of paired alignments compiled by [30] (Ovch32), plus the histidine kinase-response regulator (HisKA-RR) bacterial two-component system from Procaccini et al. [41], provided by the authors. We included HisKA-RR, because it is a well-characterized interaction with a very large, diverse multiple sequence alignment (8998 sequences for each gene pair) and genetic evidence supporting several interactions. For these reasons, HisKA-RR has also been used previously in coevolutionary analyses [42].

Because the HisKA-RR alignment is so deep, it enabled us to quantify the effects of alignment size and diversity by uniformly down-sampling the full alignment to produce a wide range of smaller pairs of HisKA and RR multiple sequence alignments. These sub-sampled alignments have six different numbers of sequences (5, 50, 250, 500, 1000, 5000), with phylogenies also sub-sampled from the original tree (Additional file 13: Figure S1). The 32 alignment pairs in Ovch32 naturally varied in size (range 216–6732 sequences) (Additional file 13: Figure S2).

In addition to the number of sequences in the alignments (N), we consider the phylogenetic diversity (PD [43]) of the alignments—also captured in the effective number of sequences (N_eff_) as calculated by PSICOV [14], the diversity within individual alignment columns measured by entropy, the alignment length (L) (i.e. the number of alignment columns), the proportion of contacting residues in the alignment.

For each pair of multiple sequence alignments from two interacting proteins, we compared every site in the first protein to every site in the second protein and scored these pairs of alignment columns for coevolution using each of the methods in Table 1. We then used coevolution scores to predict inter-domain pairs of amino acid residues that are less than 8 angstroms (Å) to each other, measured between C_*β*_s, in the representative co-crystal structure (See Methods and Table 2).

**Table 2:** TP: True positive, FP: False positive, TN: True negative, FN: False negative

	Prediction	
C _*β*_ distance	Coevolving	Not coevolving
<8Å	TP	FN
≥8Å	FP	TN

We evaluated performance using power (also called recall, sensitivity, and true positive rate (TPR)) (Eq. 1) and precision (also called positive predictive value (PPV)) (Eq. 3) at a range of low false positive rates (FPR)—the proportion of negatives falsely predicted as positives (Eq. 2). The false positive rate is equivalent to 1 - specificity. Power and precision are complementary performance measures that quantify the percentage of interacting residue pairs that are found and the percentage of identified residue pairs that are interacting, respectively. Precision is a useful measure of performance in cases where positives (contacting pairs of residues) are overwhelmed by negatives (non-contacting residues). A method with high precision is helpful for generating lists of high confidence pairs of residues for expensive follow-up studies, even if it misses a number of truly interacting sites and therefore has relatively low power. We additionally examined four threshold-independent performance measures, area under Receiver-Operator Curve (auROC), area under precision-recall curve (auPR), maximum F_1_-score (f _*max*_) (Eq. 4), maximum *ϕ* (*ϕ*_*max*_) (Eq. 5).

(1) $$ TPR = \frac{TP}{TP + FN}   $$

(2) $$ FPR = \frac{FP}{FP + TN}   $$

(3) $$ PPV = \frac{TP}{TP + FP}   $$

(4) $$ F_{1} = \frac{2 \cdot PPV \cdot TPR}{TPR + PPV}   $$

(5) $$ \phi = \frac{TP \cdot TN - FP \cdot FN}{\sqrt{(TP + FN)(TN + FP)(TP + FP)(TN + FN)}}   $$

We also evaluated performance using two stricter definitions of contacts. First, we defined contacts as residue-pairs with less than 6Å between their closest non-hydrogen atoms. We then evaluated performance in the HisKA-RR sub-alignments using a definition of contacts that, in addition to spatial proximity (C_*β*_< 8Å), requires biochemical evidence for the role of the contacting residues in determing ortholog- and paralog- specificity of the interaction (i.e. reducing cross-talk between orthologous and paralagous interacting proteins). A list of such residues in representative sequences is found in Casino et al. [44], Li et al. [45], Haldimann et al. [46], Skerker et al. [47], and Laub and Goulian [48]. Trends in the results were generally similar across these choices of definition for true interactions, but we observed some differences in performance between definitions when the false positive rate (FPR) is controlled (Additional file 13: Figure S8 and S10).

### Physically interacting sites can be accurately detected in large sequence alignments

Our primary finding is that many coevolutionary methods are able to detect inter-molecular contacts at low FPRs in alignments with hundreds of diverse sequences from each protein, consistent with previous studies of intra-molecular contacts [3, 17], specifically when the alignments are deeper than they are long [25, 26]. We capture this rectangular quality in the statistic N_*eff*_/L, where N_*eff*_ is the effective number of sequences as calculated by PSICOV [14] and L is the total number of columns in both alignments. We observe similar trends when using the number of sequences (N) or their phylogenetic diversity (PD) [43], rather than N_*eff*_/L, to compare performance.

**Fig. 1:**
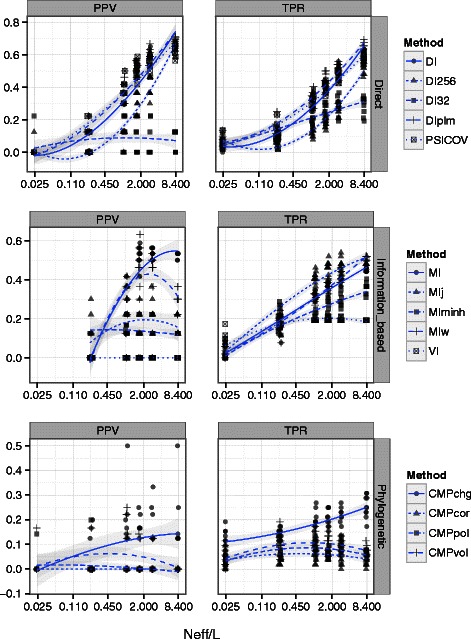
Coevolution statistics differ in their ability to detect residue contacts in HisKA-RR sub-alignments. Direct methods benefit from larger, more diverse alignments. Left: Precision (PPV) at false positive rate (FPR) < 0.1 %. Right: Power (TPR) at false positive rate (FPR) < 5 %. Blue lines indicate a loess fit to each method, 95 % confidence intervals are shown in gray. See Abbreviations and Table 1 for abbreviations

Both power and precision improve with increasing N_*eff*_/L for nearly all coevolutionary methods in the HisKA-RR data set (Fig. 1). However, for alignments with N_*eff*_/L < 1.0, power at FPR < 5 % remains relatively low (< 50 %), and even lower (< 10 %) when controlling the false positive rate more strictly (FPR < 0.1 %). Precision is expectedly higher at FPR < 0.1 % than at FPR < 5 %, but also remains below 50 % for “square” (N_*eff*_/L = 1.0) alignments. Additionally, the performance metrics f_*max*_ and *ϕ*_max_ show that there are no score thresholds (i.e. the strictness of predictions) that achieve both high precision and power in alignments with N_*eff*_/L ≲ 3.0 (Additional file 13: Figure S15-S17). Despite the smaller range in N_*eff*_/L values, these performance trends are also observed across the Ovch32 alignments (Additional file 13: Figure S11 and S19).

However, in the HisKA-RR alignment, we observed two exceptions to this trend when using the strictest definition for contacting pairs (i.e. requiring residue C_*β*_< 8Å coupled with biochemical evidence for specificity determination). First, the standard MI statistic is the most precise method for detecting contacting sites in alignments with N_*eff*_/L >1.6 and FPR < 0.1 % (Additional file 13: Figure S10, Additional file 11). Second, mutual information normalized by the joint entropy (MI_j_) has relatively high power compared to the Information-based methods and is the most powerful method for detecting contacting sites that are supported by experimental evidence at FPR < 5 % (Additional file 13: Figure S8, Additional file 12). However, MI_j_ has drastically lower power at FPR < 0.1 % (Additional file 13: Figure S9). These findings suggest MI_j_ may be useful for detecting as many contacts as possible if a moderate FPR can be tolerated. Information-based methods are straightforward to compute, adding to their utility in these settings.

CoMap performance is an interesting case because, in contrast to DI, DI _plm_, and PSICOV, it was not initially designed to find contacting residues, rather a mix of both short and long-range interactions. In the smallest alignments (5 sequences) we tested, we occasionally observe CMP _chg_ has higher power than the Direct methods (Mann-Whitney U *P* = 0.003). However, its lower performance in other alignments may indicate that it is identifying a set of coevolving residue pairs that partially overlap with contacting residues. Additionally, a filtering step necessary to run CoMap on large alignments may be limiting its performance (See Methods). It remains to explore whether CoMap can be used to prioritize residue pairs predicted by the other methods for functional assays.

Finally, we looked at the relationship between performance and the proportion of residue pairs that are contacts. Comparing across the structures in the Ovch32 data set, we observed the proportion of contacts is correlated with precision at FPR < 0.1 % (Additional file 13: Figure S24, Additional file 10). This means that most strongly coevolving residues in a protein pair are more likely to be physically interacting in co-crystal structures with a larger fraction interface residues. Power is also correlated with the proportion of contacts, though not as strongly as precision (Additional file 13: Figure S25).

### Diversity of sequences is important for accurately detecting contacts

**Fig. 2:**
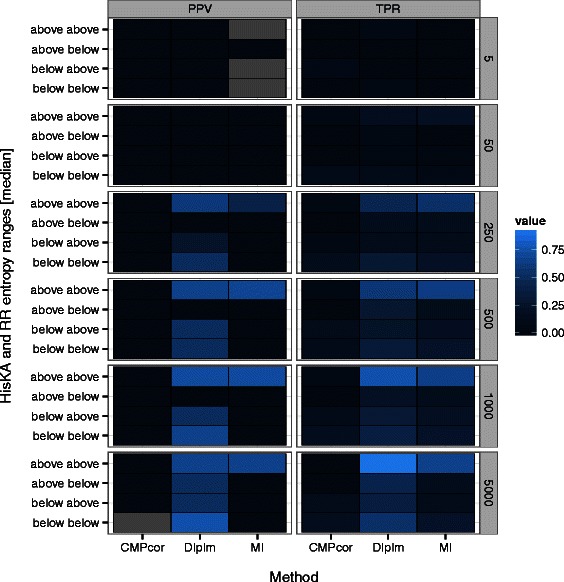
Detecting coevolving alignment columns is easier when individual alignment columns have similar levels of variation. Column pairs in the HisKA-RR sub-alignments are parsed according to above- or below- median entropy for each size alignment size (number of sequences: N). Left: Median precision (PPV) at FPR < 0.1 %. Right: Median power (TPR) at FPR < 5 %. See Abbreviations and Table 1 for abbreviations

The diversity of residues within the individual alignment columns that make up each pair is another important factor to consider. To explore this, we assessed performance among column pairs with respect to their marginal entropies. We computed power and precision separately for each rate category group (See Additional file 13: Supplemental Methods). This analysis showed that faster evolving (i.e. above-median-HisKA paired with above-median-RR) contacts are generally the easiest to detect with coevolutionary methods. Dually conserved residues (i.e. low-HisKA paired with low-RR) are the next easiest to detect (Fig. 2). We conclude that MI _w_’s drop in performance at 5000 sequences may be due to dually-variable columns being improperly reweighted. These analyses show that sequence variation quantitatively affects the accuracy of coevolution analyses, with most methods performing best when coevolving residue pairs have similar substitution rates.

To investigate whether higher power in larger alignments results primarily from the number sequences per se or depends upon the diversity of the sequences, we compared the performance across alignments with different diversity values but the same number of sequences. We quantified diversity using phylogenetic diversity (PD) [43] and the effective number of sequences as calculated by PSICOV (N _eff_) [14] (Additional file 13: Figure S5 and S6).

For HisKA-RR sub-alignments, we found weak positive and negative relationships between the nominal false positive rate and PD for some methods in alignments with 5000 sequences at given target false positive rates. For each group of equally sized alignments for each method (and for each null distribution and significance threshold), we tested whether the false positive rate correlates with PD using Spearman’s rho. Few methods had uncorrected *P*-values < 0.05 and none did when controlling for the 336 comparisons (smallest uncorrected *P*: 1.73e-3; *ρ*: 0.85 for MI _j_ at N = 5000, *P*_*empirical*_ < 0.001). Testing for a bulk correlation (ignoring method; normalizing PD by alignment size) reveals a weak positive correlation (*ρ*= 0.27, *P* < 1.9e-29) at *P*_*normal*_ and *P*_*empirical*_ < 0.05 but not < 0.001. Overall this suggests that the false positive rate may increase with more diverse sequences at loose significance thresholds. Alternatively, the PD ranges were too small to detect a relationship with false positive rate.

While the range in diversity for alignments with 5 sequences is small (PD: 7.5-11, N _eff_: 5), under the normal distribution, the false positive rate is better controlled in diverse alignments. However, under the empirical null, the Information-based methods do not control the FPR for these alignments and have larger false positive rates as diversity increases in these alignments.

One caveat of the HisKA-RR analysis is that (for computational reasons) we generated sub-alignments by random sampling and therefore only explored a range of phylogenies close to the typical diversity for each alignment size. We observe fairly strong correlations between cutoff-independent performance metrics and N _eff_ (and also N _eff_/L as L is constant in HisKA-RR). The alignments in Ovch32 provide a broader range of phylogenetic scenarios. Across these 32 interactions, N _eff_ is weakly negatively correlated with the same performance metrics (Additional file 8). However, accounting for alignment length (with N _eff_/L) reveals that there is a positive relationship between alignment depth and performance. (Additional file 9, Additional file 13: Figure S5 and S7) show that high N _eff_ alone does not guarantee good performance. For example, taking the best performing method at each alignment pair, the alignment pair with the highest N _eff_ had at best the fourth poorest *ϕ*_max_. Conversely, the third smallest N _eff_ corresponds to the third best *ϕ*_max_; and at FPR < 0.001, it had the highest precision (PPV = 63 %). Interestingly, it also has the shortest length (L = 168 columns), suggesting that perhaps taking into account the proportion of possible contacts may play an important role in estimating expected performance.

### Choice of null distribution affects performance

The previous results show performance based on the known HisKA-RR structure. In practice, when applying the methods in our study the structure usually is not known. One therefore uses a null distribution to control false predictions. Specifically, an upper quantile of the distribution of coevolutionary statistics in the absence of coevolutionary constraint is used as a threshold; one declares any pair of sites with a statistic exceeding the threshold a predicted contact. The goal is to minimize false predictions by predicting contacts only when statistics are much larger than expected by chance under the null distribution. A variety of null distributions are commonly used, including theoretical limiting distributions [8, 49, 50], the empirical distribution of observed scores (under the assumption that most pairs of sites are not coevolving) [51], and parametric, semi-parametric, and non-parametric bootstrap distributions [10, 52]. Theoretical and empirical nulls are computationally inexpensive compared to bootstrap methods, which require accurately simulating thousands of large data sets (See Additional file 13: Supplemental Text).

We used our sampled sub-alignments of HisKA-RR and the Ovch32 alignments [30] to compare the performance of two commonly used null distributions and to evaluate the sensitivity of each approach to alignment size. For each null distribution and coevolutionary statistic, we first employed the non-contact pairs of residues to assess if the FPR was truly controlled or not at given target FPRs (*α*) of 5 % and 0.1 %.

**Fig. 3:**
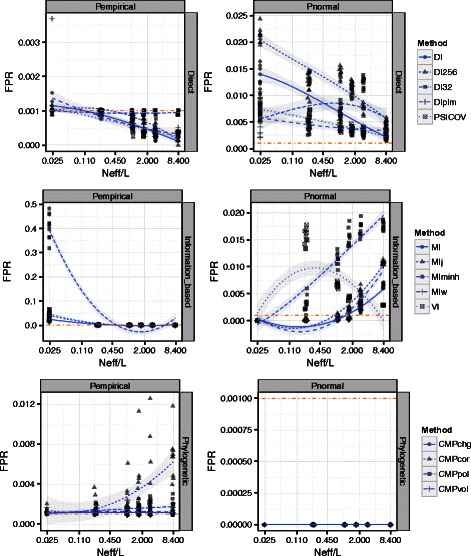
Null distributions for coevolution statistics differ in their control of the false positive rate (FPR). Nominal FPRs for a given target FPR 0.1 % (Dashed orange line) are shown for the HisKA-RR sub-alignments. Left: Nominal FPRs using the empirical distribution of score ranks as the null distribution (i.e. using *P*_*empirical*_). Right: Nominal FPRs assuming standardized scores have a standard normal null distribution (i.e. using *P*_*normal*_). Blue lines indicate a loess fit for each method, 95 % confidence intervals are shown in gray. See Abbreviations and Table 1 for abbreviations

The normal distribution can be used as theoretical null for mutual information and its normalized variants. Under this assumption, coevolution scores are standardized to Z-scores and compared to upper quantiles of the standard normal distribution (mean = 0, variance = 1). We then used the resulting upper-tail *P*-values (*P*_*normal*_) to predict contacting residue pairs. We found that nominal FPRs using this approach consistently exceed the target FPR across the range of N _eff_/L values in both the HisKA-RR sub-alignments and the Ovch32 alignments [30] (Fig. 3; Additional file 13: Figure S12-S14). In general, as N _eff_/L increases, the nominal FPR for Direct methods decreases while it increases in Information-based methods, suggesting that Direct methods truly benefit from deeper alignments. Nominal FPRs were observed to be as great as twice to 24 times the target FPR for target FPRs 5 % and 0.1 % respectively. This suggests that either non-contacting residue pairs carry signals of coevolution (e.g. due to phylogeny, structural, or other evolutionary constraints) and/or that Z-scores of coevolution statistics have variance greater than one across non-contacting residues (e.g. due to an underestimated standard deviation across residue pairs resulting from within protein constraints or residues appearing in many pairs). Three of the four phylogeny aware CoMap methods controlled the nominal FPR below the target in all cases suggesting that the charge compensation analysis is predicting long-range residue interactions as well as contacts.

Thus, while the normal distribution applied to standardized coevolution statistics can practically be used as a null distribution, we conclude that this approach results in elevated rates of false positive predictions, likely due to shared phylogeny, structural constraints affecting non-contacting residue pairs, or coevolution scores not being normally distributed (Additional file 13: Figure S30-S32). A theoretical null (e.g. non-central gamma [53]) that is parameterized for individual column pairs may therefore be more appropriate (See Additional file 13: Supplemental Text) and warrants future investigation.

Another choice of null distribution is the observed empirical distribution of the coevolution statistics. A *P*-value (*P*_*empirical*_) for a score *S* is simply the proportion of scores that are more extreme than *S*. This straightforward method can be easily applied with any statistic. However, it also assumes that no pairs of sites are coevolving and should therefore produce thresholds that are too strict when there are some coevolving sites in the data set (i.e., making it harder to predict real contacts). Although, we found that the empirical null distribution does produce nominal FPRs that exceed target FPRs (Fig. 3; Additional file 13: Figure S13). As the proportion of contacts increases, the *P*_*empirical*_-values become more conservative (Additional file 13: Figure S26 and S27). The Direct methods best control the nominal FPR in both sets of alignments, marginally exceeding the target FPR in only a couple of cases (maximum FPR/ *α*= 3.68). The Information-based methods controlled the FPR below 1.58 times *α* in the Ovch32 alignments [30], however the HisKA-RR sub-alignments reveal that at N _eff_/L < 0.3, control of the FPR is lost, especially in MI _Hmin_ (FPR/ *α*> 400). The Phylogenetic method that consistently exceeded the target FPR was the CoMap correlation analysis (CMP _cor_) which makes no assumptions regarding the biochemical properties of the amino acids. These results suggest that the empirical null distribution is not as conservative of an approach as one might expect from including contacting residue pairs in the null distribution. Although, it may suffer from some of the same effects that make the normal null distribution anti-conservative, such as shared phylogeny or structural constraints. In some methods like MI _minh_, alignments with very few sequences (e.g. 5–50) have a limited number of possible scores which leads to ties in *P*-values between contacting and non-contacting residues. If contacts and non-contacts have roughly the same *P*_*empirical*_ values, the target and nominal FPRs should be similar. But with large ammounts of ties, predictions are made in blocks, possibly forcing discontinuous jumps in the nominal FPR with respect to the target FPR. This could compound or diminish the anti-conservativeness of *P*_*empirical*_.

### Cross-species case study: applying coevolution methods to Vif-A3G identifies some residues known to affect host-virus interactions

Viral infectivity factor (Vif) is a lentiviral accessory protein whose primary function is to target the antiviral cytidine deaminase APOBEC3G (A3G) of its mammalian hosts through ubiquitination. Because the two protein families are in an evolutionary arms race [54, 55], we hypothesized that they would be an informative example for exploring the utility of coevolution methods in host-virus protein pairs (i.e. inter-protein, inter-species interactions). This is a novel application of coevolution analysis, which has primarily been applied to residues within a protein or between pairs of proteins in the same genome.

A major challenge in performing coevolutionary analysis on cross-species protein pairs is acquiring appropriate data, including paired alignments and protein structures for validation. For Vif-A3G, we were able to identify 16 pairs of sequences (N _eff_= 10.0) from different primates (A3G orthologs) and their lentiviruses (Vif orthologs) in public databases (Additional file 5). Our benchmarking results on HisKA-RR indicate that such small protein families push the useful limits of the coevolution statistics we tested (N _eff_/L = 0.014). The low sequence diversity of A3G (N _eff_= 3.04) within primates compared to Vif (N _eff_= 11.3) within primate lentiviruses also presents challenges. Hence, we expect coevolutionary analysis to potentially have limited power in this scenario. To quantitatively evaluate performance, requires validated Vif-A3G interactions. The structure of Vif in complex with A3G has not been solved. However, biochemical assays have solidly identified regions important for binding and ubiquitination along the individual reference sequences of HIV1 Vif [56–59] and human A3G [60, 61] (Table 3). For this analysis, we therefore take the residues in biochemically-validated regions to be positives even though they might not be contacts (i.e. C _*β*_ distance ≥8Å), and assume that all remaining residues are negatives, even though other sites (including sites deleted in these reference sequences) are possibly involved in the interaction. While further experimentation is needed to understand the relationship between functionally important sites and the structure of the protein interaction, as well as the effects of mutations in these sites on the fitness of lentiviruses, we explore whether any clues can be identified in the limited data that describes the coevolutionary history of the Vif-A3G residues.

**Table 3:** Important residues for the Vif-A3G interaction

	Position	Notes
Vif	21–23,26	A3G-specific
	30	
	40–44	
	55–72	A3G and A3F
A3G	121–149	essential for Vif-binding

HIV1 Vif [56–59]. Human A3G [60, 61]

First, we computed a subset of coevolutionary statistics for all Vif-A3G residue pairs and evaluated how well the statistics pinpoint the positive functionally important residues compared to negatives. For this evaluation, we used the empirical distribution of scores as a null distribution to determine statistical significance (i.e., *P*_*empirical*_) because they have lower false positive rates across N _eff_/L values at strict significance thresholds. Because the positives and negatives are single residues in each sequence instead of inter-protein residue pairs, we summarized *P*_*empirical*_ for each residue by assigning it the most significant *P*_*empirical*_ across all inter-protein pairs to which it belongs, and then explored the Vif and A3G results individually (Additional file 7). From our benchmarking on the bacterial data sets, we know that significance thresholds that control the FPR vary by method and N _eff_/L, and that strict thresholds that yield very low (∼ 2–3 %) power are typically needed to control FPR in small alignments. we therefore chose to identify a significance threshold for each method that maximizes precision on the known functional sites in each protein. Then, we estimated power and FPR at these thresholds.

On Vif, with the exception of CMP _cor_ and DI_32_, the maximum precisions for each method ranged from 9 to 20 % (i.e. only one or two residues out of ten predicted to be positives are truly positives) (Additional file 13: Figure S34). At these precision-optimized thresholds, MI _j_ and MI _minh_ predict almost every Vif residue to be coevolving; a stricter threshold would not result in a lower proportion of incorrect predictions. In contrast, the precisions for CMP _pol_, CMP _cor_, DI_32_ are the highest (20 %, 40 %, 100 % respectively). However, this comes at the cost of making the fewest number of predictions with the latter only making a single prediction. For these methods, less strict thresholds are needed to identify a greater proportion of positives at the cost of increasing the proportion of false discoveries. Across all methods, low f _max_ and *ϕ*_max_ values (0.26 and below) suggest there are no significance thresholds that balance power and precision for this data set.

**Fig. 4:**
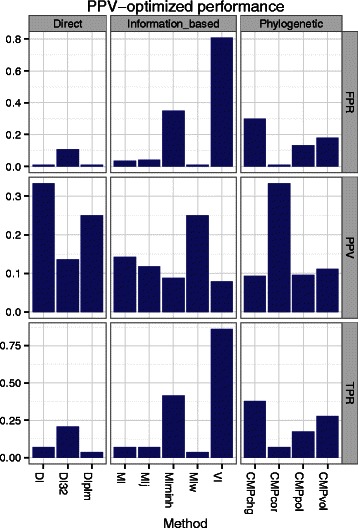
Power (TPR), precision (PPV), and false positive rate (FPR) for predicting antiviral protein A3G residues (not pairs) essential for interacting with its viral antagonist Vif at *P*_*empirical*_ <*α* thresholds that maximize PPV for each coevolution method. Residues defined as positive are taken from previous functional mutation studies in Table 3. See Abbreviations and Table 1 for abbreviations

**Fig. 5:**
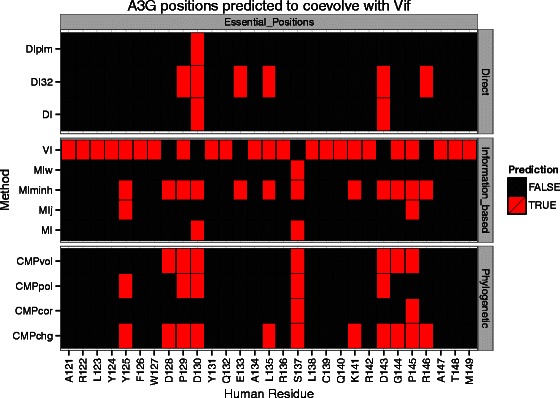
A3G residues currently known to be essential for binding its viral antagonist Vif. Predictions of residues that coevolve with Vif (red) made at a threshold that maximizes precision (PPV) using currently known essential residues identify position D130 which was previously implicated in species specific resistance

We observed similarly low performance on A3G (Fig. 4). Encouragingly, we note that positions 128-130 are correctly identified by multiple methods (Fig. 5). Residues at position 130 (e.g., D vs A) are highly likely to be adaptations that conferred species-specific resistance to Vif-induced degradation in Old World Monkeys 5-6MYA [54, 55]. Position 128, that also provides species-specific resistance, is thought to be more recent [54, 55, 62]. While these coevolution methods alone may not yet be accurate enough to identify functional residues, they potentially enhance other evolutionary analyses. For example, of the many Apobec sites under positive selection [55], it is reasonable that lentiviruses are more likely shaping the evolution of those sites that coevolve with Vif than sites that coevolve with other viral or virus-like agents.

Secondly, we visualized the localization of Vif residues predicted to be coevolving with A3G on a partial structure of Vif in complex with cofactors utilized for protein ubiquitination [63] (Additional file 7, Additional file 13: Figure S36). In [63], the authors are able to see that a critical subset of the Vif positives is solvent-exposed. We re-evaluated performance with only these residues as the positives (Table 3). There is poor precision to identify the putative solvent-exposed interface among the methods; CMP _cor_ at 40 % and CMP _vol_ at 10 % are the only methods with precision > 6 % (Additional file 13: Figure S35).

Our analysis of the Vif-A3G interaction confirms that power to detect functionally important residues in each protein family is also low in inter-protein analyses between species, even though it is plausible that an arms race between lentivirus and mammal would give rise to stronger signals of coevolution compared to background. It is important to consider that perhaps the positions considered positives may not all be of equal evolutionary importance across primates. Interfaces may be gained or lost and the rapid evolution of the two proteins likely produces many alternative solutions to maintaining an antagonistic interaction. There were many predicted positions that were not in the positives and further systematic validation and more comprehensive sequencing of lentiviruses and primates is needed to determine which pairs of residues are actually in close proximity or functionally required for other reasons. Additionally, there appears to be some level of complementarity in the predictions made by VI and MI _minh_ and the CMP methods, which measure different biochemical trade offs between coevolving residues. This strengthens the rationale for integrating methods to better predict interface residues experiencing potentially different evolutionary constraints (e.g. structural, catalytic activity, specificity). Coevolutionary analysis can help to generate and prioritize candidates for these experiments.

### A toolkit for inter-molecular coevolution analysis

Due to the diversity of coevolution methods and the time spanned during which they were developed, it is no surprise that they vary widely in the input and output formats they tolerate. Additionally, many of the coevolution methods we tested are computationally expensive, so we prepared our workflow to take advantage of multiprocessing workstations and high performance computing clusters. We outline a few utilities we developed to aid in processing sequences, structures, and coevolution results for benchmarking and making predictions and visualizations.

Our toolkit consists of three parts, (1) a collection of wrappers for running the coevolution programs from the command line and where possible in a Sun Grid Engine super computing environment (https://github.com/aavilahe/coevo_tools), (2) an R package for evaluating performance and calculating *P*_*empirical*_ and *P*_*normal*_ (https://github.com/aavilahe/coevo_analysis_Rpackage), and (3) pre- and post-processing utilities to facilitate input and output format management, mapping alignments to structural models, and visualizing coevolving residues on protein structures (https://github.com/aavilahe/coevo_analysis_pypackage).

We also implemented the canonical mutual information statistic, the normalizations of mutual information in Martin et al. [64], and VI, the information theoretic distance described by Meila [65] (https://github.com/aavilahe/infcalc).

#### File formats

The coevolution methods we tested accept three different file formats and alignments as two separate files or one horizontally concatenated file. The different formats, (fasta, phylip, raw reads) store more or less meta-data and have limits on the length of sequence identifiers.

Our coevo package at https://github.com/aavilahe/coevo_analysis_pypackage depends on the Biopython library and contains many auxiliary functions and executable python scripts for input file preparation.

A typical processing step may involve truncating sequence identifiers when converting between sequence formats, taking care that they remain informative and unique. For example:

 Some methods require one concatenated alignment, while others read in two separate ones.



The coevolution methods return tab, space, or comma delimited output with and without headers. The scores returned are often indexed by column numbers of the concatenated alignment and not the original two alignments of interest, and can be numbered starting from 0 or 1.

The scores module in our coevo package includes definitions for the various formats we encountered, extracts the relevant indices and scores, optionally merges results from different methods, and processes them to a standard tab delimited format with appropriate headers and indices that correspond to the alignments of interest. For example:



#### Structure

Another important procedure is to map column numbers from a given alignment to a reference PDB structure. For example, we used map_column_to_resnum.py, and get_dists.py to map atomic distances to column-pairs in existing alignments in order to compare them to coevolution scores and *P*-values and to validate predictions. The HisKA-RR complex in (PDB: 3DGE) is actually an ABAB tetramer—two sets of identical chains form a structure such that a HisKA chain will make contact with two RR chains. One can use min_dists.py to get the minimum distances between residues from both interactions. For a detailed example, see https:// github.com/aavilahe/coevo_analysis_pypackage/blob/dev/ example/pdb_tests/example_3DGE_column_distances.sh.

Visualization of coevolution score summaries on individual residues can be accomplished by generating an attributes file for use with UCSF Chimera [66] using make_attributes.py (e.g. Additional file 7, Additional file 13: Figure S36 shows Vif residues predicted to coevolve with A3G, each Vif residue is colored by most significant *P*-value out of all A3G residues).

## Discussion

In this work we aimed to paint a picture of the performance of emerging methods to identify inter-protein contacts using coevolution and to identify properties of alignments where performance is expected to be best. As previously noted in intra-protein predictions [3, 9, 14], re-weighting of the sequences to account for the underlying phylogeny is important for inter-protein predictions as well, however as the comparison between MI _w_ and MI shows, it is important to tune the parameters controlling the re-weighting in cases where there are fast evolving alignment columns in an overall conserved protein family. Fortunately, methods that search for direct correlations—using a global statistical model for the sequence alignments—seem to be able to correct for the improper weighting (compare MI _w_ to DI). These methods are more precise at strict false positive rates than their counterparts especially when the alignments have N _eff_/ L < 1.0. However, it may be beneficial to use a faster, MI-based method if the use case allows for a relaxed FPR and is concerned with power versus precision.

We also investigated the use of three null models to control the false positive rate. Counter-intuitively, a null model that explicitly models evolution independently for each alignment fails to control the false positive rate. We believe that our simulated alignments are systematically scoring too low because they fail to capture the correct amount of variation in the observed alignments, resulting in artificially significant *P*-values, except for when the effects of having small alignment sizes results in overly conservative *P*-values. Using a standard normal or the empirical distribution of scores as null models also failed to control the false positive rate, likely due to the correlation structure imposed by the shared evolutionary history of the residues, the distribution of evolutionary rates of the residues, or because asymptotic assumptions do not hold at small sample sizes. Thus, choosing an appropriate *P*-value cutoff in a real analysis when the structure is unknown and alignment depth is shallow still remains a challenge. However, we show that in diverse enough alignments the empirical null successfully controls the false positive rate for Direct methods. As a future direction, we suggest exploring theoretical null distributions that can be parameterized for individual alignment column pairs such as [53] or further improving protein evolution simulators to generate distributions of scores where the evolutionary rates are more similar between the null and alternate hypothesis.

These results are encouraging, but still leave us with the challenge of how to choose an appropriate *P*-value cutoff in a real analysis when the structure is unknown. Since our findings indicate that nominal FPRs exceed target FPRs using *P*_*normal*_ and *P*_*empirical*_ for nearly all methods, stricter *P*-value cutoffs than the target false positive rate seem warranted. But it is not clear how much stricter will be needed in any given alignment pair. Additional information to consider when making such modifications should include incorporating alignment properties such as N _eff_/L, and the expected proportion of contacts expected to exist (Additional file 13: Figure S27; Fig. 3). However, large data sets of many protein interactions are needed in order to be confident in parameters or prior probabilities to be used to correct the *P*-values. Hence, in most applications one must simply aim to control a target FPR, knowing that the true error rate is likely to be larger. For this reason, the empirical null distribution may be the best choice to use as it controls error rates across the majority of alignment sizes, target FPRs, and coevolution methods tested (Fig. 3; Additional file 13: Figure S13). As a rule of thumb, the empirical null overall controls the FPR for the Direct methods, however in small alignments (5 sequences or N _eff_/L < 0.3) it can be up to 1.5 times the target FPR. For the purposes of data collection and experimental design, we therefore recommend sequencing phylogenetically deeply enough to attain N _eff_/L > 1.0 to control FPR and > 2.0 to ensure modest TPR and PPV.

A related problem to the one discussed here is to search a large set of protein pairs (within or between species) to determine which ones are interacting. In this setting, coevolution method performance is potentially more important than when predicting contacting residues for known interactions, because the search space will contain so many negatives (i.e., non-interacting pairs). A permissive *P*-value cutoff will lead to a large number of false positives and that may misinform investigators, while being too strict will lead to false negatives that keep potentially important findings hidden. It would be interesting to understand if thresholds and the methods for choosing them generalize to all protein-protein interactions. Different experimental techniques have strengths and weakness in identifying different types of interactions. Interactions may be transient, but highly critical, or tightly binding but too conserved to detect any sequence variation among the sequenced orthologs Mulberry Ideally, we would like to understand what a null model teaches us about phylogeny-induced correlations when structural inter- or intra-protein constraints are minimal, perhaps at an evolutionary stage where a protein interaction is acquired or lost. What can this reveal about the birth and death of protein interactions, regulatory networks, and neofunctionalization? Another challenge for predicting interacting protein pairs from coevolutionary tests is how to summarize statistics for individual pairs of residues to produce a single score for a pair of proteins. Although outside the scope of our work, such a strategy would likely involve comparing tails of score or *P*-value distributions. Deciding on how to define how much of the tail to consider will depend highly on having an estimate of the false positive rate. Based on some preliminary investigations of these questions, we conclude that it is unlikely that cross-species interacting protein pairs can be accurately distinguished from non-interacting pairs on a genome-wide scale.

The progress of high-throughput interaction mapping highlights the need for continued refinement of inter-protein coevolution detection methods. Given that improper re-weighting of sequences can negatively affect power and the false positive rate, perhaps expanding Direct methods to independently obtain sequence weights for each alignment or using an evolution-based probabilistic weight (such as in CoMap or using phylogenetic logistic regression) for unusual variation in each column is a logical next step forward. Another important contribution would be to develop a generalizable null model that can help differentiate contacts when there are very few sequences available for protein families. Furthermore, investigating the correlations among the coevolution statistics themselves in inter-protein data sets could potentially disentangle structural from non-structural coevolutionary forces as well as serving to construct an ensemble method. Comprehensively sequencing orthologous pairs of protein families is a straightforward way to test the usefulness of these future contributions while simultaneously enabling current methods to perform to their fullest.

## Conclusion

We benchmarked 13 coevolution methods on 33 protein interactions with associated sequence alignments of varying depths. We conclude that coevolutionary analyses of cross-species protein-protein interactions is largely hindered by a lack of phylogenetically deep protein alignments for many proteins, and furthur demonstrate this in an example case involving an HIV1-human protein interaction. Additionally, we report that commonly used null distributions generally fail to control false positives in coevolutionary analyses, though errors are best controlled by the empirical null in large alignments.

## Methods

### Multiple sequence alignments

A master alignment of 8998 horizontally concatenated HisKA and RR sequences from Procaccini et al. [41] was graciously provided by the authors (Additional files 2 and 3). From this alignment, aligned sequences were sampled uniformly (each sequence had equal probability of being sampled) to create sub-alignments with 5, 50, 250, 500, 1000, and 5000 sequences. We sampled 10 sub-alignments of each alignment size (number of sequences in sub-alignment), resulting in 60 total alignment pairs (Additional file 4).

The Ovch32 alignments [30] were downloaded from complexes section of the Baker lab website (http://gremlin.bakerlab.org/complexes/PDB_benchmark_alignments.zip) on Aug 29, 2014 (Additional file 1). A stable link is located at the Dryad repository, doi:10.5061/dryad.s00vr/7 [67]. The corresponding structures were downloaded from PDB and processed to obtain contacts between representative protein chains. See Supplemental Files for accessions. Columns comprised of more than 75 % gaps were removed as in [30]. Additionally, only columns mapping to the representative structure were kept.

The CoMap implementation requires a preprocessing step to remove sequence redundancy (a data munging alternative to sequence weighting). This additional step was also necessary to prevent buffer underflow errors when evaluating likelihoods in very large input trees. Therefore, all alignments with more than 200 sequences were culled to contain the 200 most diverse sequences before being passed to CoMap. The sub-alignment used corresponds to the 200-leaf sub-tree that maximizes PD for each original input alignment and tree.

### Measuring coevolution

The coevolution methods benchmarked are listed in Tables 1 and 4. Wrappers for the Direct methods are provided in our coevo_tools code repository to facilitate running from the command line (See Supplement for details). For methods in the plmDCA, mfDCA and hpDCA packages, MATLAB, or the MATLAB runtime executable is required as well as various MATLAB Toolbox dependencies and licenses. Default settings were used for all methods, including sequence re-weighting and APC. DI_32_ and DI_256_ are variations of DI in the hpDCA package with an additional parameter for tuning dimensionality reduction, “p”, set to 32 and 256 respectively as it had no default (a selection from a parameter search in [68]).

**Table 4:** Versions and sources of coevolution methods benchmarked

	Method	Software package	Version	URL
Information-based	MI	infCalc	v0.1.2	https://github.com/aavilahe/infcalc
	VI			
	MI_j_			
	MI_Hmin_			
	MI_w_	DCA	“2011/12”	http://dca.rice.edu/portal/dca/download
Direct	DI			
	DI_256_	Code S1 in [68]	“2013”	http://doi.org/10.1371/journal.pcbi.1003176.s002
	DI_32_			
	DI_plm_	plmDCA	symmetric_v2	http://plmdca.csc.kth.se/
	PSICOV	PSICOV	V1.09	http://bioinfadmin.cs.ucl.ac.uk/downloads/PSICOV/
Phylogenetic	CMP_cor_	CoMap	1.5.1b5	http://home.gna.org/comap/doc/html/index.html
	CMP_chg_			
	CMP_vol_			
	CMP_pol_			

### Evaluating coevolution performance

For each method, coevolution scores for pairs of amino acid positions were used to predict inter-domain pairs of amino acid residues that are close to each other in the representative co-crystal structure (PDB ID: 3DGE).

As previously described in Ezkurdia et al. [69], Monastyrskyy et al. [70], Jones et al. [14], and to be consistent with Morcos et al. [9], we define *positives* as pairs of alignment positions mapping to amino acid residues whose beta carbons (C _*β*_) are less than 8 angstroms apart in 3DGE. All other pairs of alignment positions are considered *negatives*.

We considered the following two alternative definitions of *positives*:

- Closest non-hydrogen atom-atom distance between residues is less than 6 angstroms [14]
- C _*β*_ distance is less than 8 angstroms *and* at least one residue is mentioned as important in determining specificity of the HisKA-RR interaction in [44–48].

Residue pairs are predicted as coevolving if their scores or *P*-values are above a given threshold (eg. top 1 %, *P* < 0.05) (Table 2).

### Phylogenetic diversity

Phylogenetic diversity (PD) is calculated as the sum of the branch lengths in a tree built from the concatenated multiple sequence alignment of both proteins. Trees were built using FastTree (version2.1.7 SSE3) with options -gamma -nosupport -wag.

## Additional files

Additional file 1
**PDB IDs from [** 67 **].** (CSV 0.46 kb)

Additional file 2
**Accessions for HisKA-RR alignment.** (CSV 796 kb)

Additional file 3
**HisKA-RR master alignment.** (ZIP 1136 kb)

Additional file 4
**Sequence identifiers for the 60 subsampled alignments from the HisKA-RR master alignment.** (CSV 5611 kb)

Additional file 5
**Accessions for Vif-A3G analysis.** (CSV 0.73 kb)

Additional file 6
**Uniprot identifiers for lentivirus proteins in HIV1-human interactome analysis.** (CSV 0.803 kb)

Additional file 7
***P***_***empirical***_ **for Vif residues as an attributes file for use with UCSF Chimera [** 66 **].** (CSV 163 kb)

Additional file 8
**Spearman test for correlations between cutoff independent metrics (** ***ϕ***_**max**_ **, f**_**max**_ **, auPR, auROC) and N**_**eff**_ **for Ovch32 and HisKA-RR data sets.** (CSV 0.24 kb)

Additional file 9
**Spearman test for correlations between cutoff independent metrics (** ***ϕ***_**max**_ **, f**_**max**_ **, auPR, auROC) and N**_**eff**_ **/L for Ovch32 and HisKA-RR data sets.** (CSV 0.24 kb)

Additional file 10
**Spearman test for correlations between precision (PPV) and the proportion of contacts for Ovch32 and HisKA-RR data sets.** (CSV 0.13 kb)

Additional file 11
**Mann-Whitney tests for difference in precision between MI and other methods for N**_**eff**_ **/L > 1.6 and FPR < 0.1 % in the HisKA-RR sub-alignments, with contacts defined from experimental studies.** (CSV 0.48 kb)

Additional file 12
**Mann-Whitney tests for difference in power between MI**_**J**_ **and other methods for N**_**eff**_ **/L > 1.6 and FPR < 5 % in the HisKA-RR sub-alignments, with contacts defined from experimental studies.** (CSV 0.47 kb)

Additional file 13
**Figure S1.** HisKA-RR. Number of effective sequences (N _eff_) versus number of sequence (N) in the 60 sub-sampled HisKA-RR alignments. Dashed line indicates the diagonal. Blue line indicates a linear fit with 95 % confidence intervals in gray. **Figure S2.** Ovch32. Number of effective sequences (N _eff_) versus number of sequence (N) in the Ovch32 alignments. Dashed line indicates the diagonal. Blue line indicates a linear fit with 95 % confidence intervals in gray. **Figure S3.** Distribution of C _*β*_ distances in HisKA-RR interaction (PDB: 3DGE). **Figure S4.** Distribution of C _*β*_ distances in Ovch32 interactions [67] (See supplemental file for PDB accessions). **Figure S5.** Ovch32. Precision (PPV) versus N_eff_ at FPR < 0.1 %. Blue lines indicate a loess fit to each method, 95 % confidence intervals are shown in gray. **Figure S6.** Ovch32. Power (TPR) versus N_eff_ at FPR < 5 %. Blue lines indicate a loess fit to each method, 95 % confidence intervals are shown in gray. **Figure S7.** Ovch32. *ϕ*_max_ versus N_eff_. Blue lines indicate a loess fit to each method, 95 % confidence intervals are shown in gray. **Figure S8.** HisKA-RR alt.. Power (TPR) vs N_eff_/L at FPR < 5 %. A stricter definition of positives, defined experimentally in [46–48] is used. Blue lines indicate a loess fit to each method, 95 % confidence intervals are shown in gray. **Figure S9.** HisKA-RR alt.. Power (TPR) vs N_eff_/L at FPR < 0.1 %. A stricter definition of positives, defined experimentally in [46–48] is used. Blue lines indicate a loess fit to each method, 95 % confidence intervals are shown in gray. **Figure S10.** HisKA-RR alt.. Precision (PPV) vs N_eff_/L at FPR < 0.1 %. A stricter definition of positives, defined experimentally in [46–48] is used. Blue lines indicate a loess fit to each method, 95 % confidence intervals are shown in gray. **Figure S11.** Ovch32. Power (TPR) at FPR < 5 % and Precision (PPV) at FPR < 0.1 % versus N_eff_/L. Blue lines indicate a loess fit to each method, 95 % confidence intervals are shown in gray. **Figure S12.** HisKA-RR. Nominal false positive rate (FPR) for target FPR 5 %. **Figure S13.** Ovch32. Nominal false positive rate (FPR) for target FPR 0.1 %. **Figure S14.** Ovch32. Nominal false positive rate (FPR) for target 5 %. **Figure S15.** HisKA-RR. *ϕ*_max_. **Figure S16.** HisKA-RR. *F*_max_. **Figure S17.** HisKA-RR. Area under precision-recall curve. **Figure S18.** HisKA-RR. Area under ROC curve. **Figure S19.** Ovch32. *ϕ*_max_. **Figure S20.** Ovch32. *F*_max_. **Figure S21.** Ovch32. Area under precision-recall curve. **Figure S22.** Ovch32. Area under ROC curve. **Figure S23.** HisKA-RR. Median precision (PPV) at FPR <0.1 % and median power (TPR) at FPR < 5 % per rate categories of individual alignment columns. Rate categories are defined as above- and below- median entropy for the HisKA and RR columns in each set of 10 alignments of equal size (number of sequences (N)). **Figure S24.** Ovch32. Precision (PPV) versus the proportion of contacting pairs of residues in each interaction (i.e. contacting pairs divided by all pairs of residues) at FPR < 0.1 %. **Figure S25.** Ovch32. Precision (PPV) versus the proportion of contacting pairs of residues in each interaction (i.e. contacting pairs divided by all pairs of residues) at FPR <5 %. **Figure S26.** Ovch32. False positive rate (FPR) versus the proportion of contacting pairs of residues in each interaction (i.e. contacting pairs divided by all pairs of residues) at *P* < 0.05 **Figure S27.** Ovch32. False positive rate (FPR) versus the proportion of contacting pairs of residues in each interaction (i.e. contacting pairs divided by all pairs of residues) at *P* < 0.001 **Figure S28.** HisKA-RR. The phylogenetic methods CTMP and Spidermonkey successfully ran on a subset of our alignments. Power (TPR) at FPR < 5 % and precision (PPV) at FPR < 0.1 %. Select methods are included for comparison. Blue line indicates a linear fit with 95 % confidence intervals in gray. **Figure S29.** HisKA-RR. The phylogenetic method CTMP and Spidermonkey successfully ran on a subset of our alignments. Nominal false positive rate (FPR) at target FPR 0.1 %. Select methods are included for comparison. Blue line indicates a linear fit with 95 % confidence intervals in gray. **Figure S30.** HisKA-RR. Quantile quantile plots of standardized coevolution scores are not always normally distributed. Scores are from 10 alignments with 5 sequences. **Figure S31.** HisKA-RR. Quantile quantile plots of standardized coevolution scores are not always normally distributed. Scores are from 10 alignments with 500 sequences. **Figure S32.** HisKA-RR. Quantile quantile plots of standardized coevolution scores are not always normally distributed. Scores are from 10 alignments with 5000 sequences. **Figure S33.** HisKA-RR. *P*_boostrap_ fails to control the FPR except for PSICOV at target FPR < 5 % in HisKA-RR alignments. Eliminating residue pairs with large simulation errors shows PSICOV and MI_Hmin_ are most robust to variation at individual sites. See Misc. Abbreviations and Table 1 for abbreviations. **Figure S34.** Vif. Power (TPR), precision (PPV), and false positive rate (FPR) for predicting viral protein Vif residues (not pairs) essential for interacting with its host target A3G at *P*_empirical_ <*α* thresholds that maximize PPV for each coevolution method. Residues defined as positive are taken from previous functional mutation studies in Table 3. See Abbreviations and Table 1 for abbreviations. **Figure S35.** Vif. Power (TPR), precision (PPV), and false positive rate (FPR) for predicting viral protein Vif residues (not pairs) essential for interacting with its host target A3G at *P*_empirical_ <*α* thresholds that maximize PPV for each coevolution method. Residues defined as positive are taken from previous functional mutation studies in Table 3. See Abbreviations and Table 1 for abbreviations.Vifcrit PPVoptbars **Figure S36.** Residues (red) on viral protein Vif (light blue) that are predicted to coevolve with it host target A3G (structure unknown). Cofactors are shown in gray. Predictions are made at a threshold that maximizes precision (PPV) using **A** known essential residues (Table 3) using **B-D** MI, **Figure S37.** HIV1-human. Distinguishing HIV1-human interactors from a protein pairs in a permuted network is difficult with small N_eff_">/L. *ϕ*_max_ across a the number of predicted coevolving column-pairs per protein-pair versus *Pˆp*_empirical_ threshold for making column-pair predictions. Blue line indicates a linear fit with 95 % confidence intervals in gray. **Figure S38.** HIV1-human. N _eff_/L distribution of alignments in HIV1-human interactors The minimum N _eff_/L seen in the HisKA-RR (red) and Ovch32 (orange) data sets is marked. (ZIP 28364 kb)
