# Supplementary material for: Coevolutionary analyses require phylogenetically deep alignments and better null models to accurately detect inter-protein contacts within and between species
Source: BMC Bioinformatics. 2015 Aug 25;16:268. doi: 10.1186/s12859-015-0677-y (PMC4549020; doi:10.1186/s12859-015-0677-y)
Supplement: Additional file 13 — Figure S1. HisKA-RR. Number of effective sequences (N eff) versus number of sequence (N) in the 60 sub-sampled HisKA-RR alignments. Dashed line indicates the diagonal. Blue line indicates a linear fit with 95 % confidence intervals in gray. Figure S2. Ovch32. Number of effective sequences (N eff) versus number of sequence (N) in the Ovch32 alignments. Dashed line indicates the diagonal. Blue line indicates a linear fit with 95 % confidence intervals in gray. Figure S3. Distribution of C β distances in HisKA-RR interaction (PDB: 3DGE). Figure S4. Distribution of C β distances in Ovch32 interactions [67] (See supplemental file for PDB accessions). Figure S5. Ovch32. Precision (PPV) versus Neff at FPR < 0.1 %. Blue lines indicate a loess fit to each method, 95 % confidence intervals are shown in gray. Figure S6. Ovch32. Power (TPR) versus Neff at FPR < 5 %. Blue lines indicate a loess fit to each method, 95 % confidence intervals are shown in gray. Figure S7. Ovch32. ϕ max versus Neff. Blue lines indicate a loess fit to each method, 95 % confidence intervals are shown in gray. Figure S8. HisKA-RR alt.. Power (TPR) vs Neff/L at FPR < 5 %. A stricter definition of positives, defined experimentally in [46–48] is used. Blue lines indicate a loess fit to each method, 95 % confidence intervals are shown in gray. Figure S9. HisKA-RR alt.. Power (TPR) vs Neff/L at FPR < 0.1 %. A stricter definition of positives, defined experimentally in [46–48] is used. Blue lines indicate a loess fit to each method, 95 % confidence intervals are shown in gray. Figure S10. HisKA-RR alt.. Precision (PPV) vs Neff/L at FPR < 0.1 %. A stricter definition of positives, defined experimentally in [46–48] is used. Blue lines indicate a loess fit to each method, 95 % confidence intervals are shown in gray. Figure S11. Ovch32. Power (TPR) at FPR < 5 % and Precision (PPV) at FPR < 0.1 % versus Neff/L. Blue lines indicate a loess fit to each method, 95 % confidence intervals are shown in gray. Figure S12 [file 12859_2015_677_MOESM13_ESM.zip › 12859_2015_677_add13/supplement_manuscript.pdf]

Supplemental Text: Coevolutionary analyses require phylogenetically deep alignments and better null models to accurately detect inter-protein contacts within and between species

Aram Avila-Herrera[1,2] <aram.avilaherrera@ucsf.edu>, *Katherine S. Pollard[1,2,3,4] <katherine.pollard@gladstone.ucsf.edu>

[1]Bioinformatics Graduate Program [2]Gladstone Institute of Cardiovascular Disease [3]Department of Epidemiology and Biostatistics [4]Institute for Human Genetics, University of California, San Francisco, CA 94158, USA

*Corresponding author

Supplemental Text

Performance by column entropy categories

For a subset of methods, we measured the performance of the coevolution methods in pairs of columns with different rates of evolution. For each alignment size, the column entropies for each of the 10 HisKA and RR sub-alignments were aggregated and their median calculated. Then, for each sub-alignment, column pairs were binned into one of the following four categories:

1. above-median-HisKA-entropy + above-median-RR-entropy
2. above-median-HisKA-entropy + below-median-RR-entropy
3. below-median-HisKA-entropy + above-median-RR-entropy
4. below-median-HisKA-entropy + below-median-RR-entropy

Then for each category, true positive rate, and precision were calculated at false positive rates below 5% and 0.1% respectively. The median performances per category are visualized in Figure S23.

Most methods perform best on pairs of alignment columns with similar sequence variation in the two proteins

To explore the effect of substitution rate variation across sites in HisKA and RR, we parsed our performance results according to the entropy of the two alignment columns (one from each gene) in every pair of evaluated sites. For each alignment size, we split columns into below- versus above-median entropy separately for each gene, and then classified pairs of sites into the resulting four groups (see Methods). Then we computed power and precision separately for each rate category group. This analysis showed that faster evolving (i.e.,

above-median-HisKA paired with above-median-RR) contacts are generally the easiest to detect with coevolutionary methods. Dually conserved residues (i.e., low-HisKA paired with low-RR) (Figure 3) are the next easiest to detect. We conclude that $\mathrm{MI_w}$'s drop in performance at 5000 sequences may be due to dually-variable columns being improperly reweighted. These analyses show that sequence variation quantitatively affects the accuracy of coevolution analyses, with most methods performing best when coevolving residue pairs have similar substitution rates.

Simulating independently evolving pairs of alignments

To extend CoMap-like *P*-values to other methods by simulating pairs of independently evolving protein alignments, we developed a semi-parametric simulation pipeline that combines software from the RAxML or FastTree, ANCESCON, Revolver, and HMMER3 packages [29], [24], [1], [14], [7] to estimate phylogenies from pairs of sequence alignments and then use these fitted models to generate large collections of protein sequence alignments that closely match observed protein families in alignment length, alignment size, phylogenetic diversity, amino acid composition, and domain architecture, in the absence of coevolution (see Methods). An alpha-testing version of our simulation pipeline is available at https://github.com/aavilahe/simulate_tools.

A non-parametric bootstrap method—recently used in an interprotein analysis in [17]—shuffles individual alignment columns tens of thousands of times. This shuffling method is faster than semi-parametric simulations, conserves the entropy and composition of the observed columns, however it does not preserve the relationships between the sequences, potentially leading to null distributions that are overly anti-conservative.

In order to classify pairs of sites as coevolving or not coevolving using a semi-parametric bootstrapped null distribution, we calculated a P-value for the score at every pair of positions by comparing the observed score to the distribution of scores simulated for that pair under the null hypothesis (independent coevolution).

To simulate alignments, we used FastTree (version 2.1.7 SSE3) [24] to build maximum likelihood phylogenetic trees for the HisKA and RR protein families. We used hmmbuild from the HMMER3 package [7] (version 3.0 March 2010) to build a profile hidden Markov model (pHMM) for each family. We sampled amino acid residues from a first order Markov chain to generate an initial sequence for each family. Finally, we used Revolver (version 1.0) [14] to simulate 1000 alignments for each family independently. Revolver can simulate the evolution of a given root sequence that adheres to the domain constraints imposed by a pHMM, and preserves a similar phylogenetic history to the observed alignment. Revolver used the WAG substitution matrix and indel probabilities were set to zero in order to simulate constant length alignments. Gaps from the observed alignment were then overlaid on the simulated alignment. We automated this process in a pipeline available at https://github.com/aavilahe/simulate_tools.

A third type of null distribution is based on employing bootstrap methods to resample the observed alignment in ways that break coevolutionary correlation or to generate alignments from a model without coevolution. These approaches have the benefit that they directly account for phylogenetic effects in the null distribution and therefore have the potential to more accurately control FPRs but are computationally intensive and not suitable for all methods as they can greatly increase computational time. To explore this possibility, we implemented a semi-parametric bootstrap null distribution for the phylogeny unaware methods in the HisKA-RR sub-alignments as an example of this approach.

This null distribution aims to resemble the observed alignments in terms of substitution rates and patterns, but substitutions are generated independently in HisKA and RR and are therefore not correlated beyond any correlation induced by similarities in the phylogenies of the two gene families. Unfortunately, we found that *P*-values calculated using the bootstrap null distribution were heavily influenced by the error in simulating alignment columns with appropriate amino acid variation. Simulation error increased with alignment size, as did nominal FPRs. Residue pairs for which the bootstrap simulated alignment columns have too much sequence variation tend to have small *P*-values, regardless of whether or not they are contacting residues. Consequently, at a target FPR of 5%, the nominal FPR was not adequately controlled for alignments with more than 5 sequences ($N_{eff}/L = 0.02$) for any method except PSICOV. Interestingly, PSICOV is the method least affected by the simulation error.

Recalculating the nominal FPR using only alignment column pairs that were moderately well simulated (no more than 250 of 1000 simulations were over or under conserved) showed much lower FPR for all methods except MI_{Hmin} (Supplemental Figure S19). MI and VI are controlled below a target FPR <5%. At a stricter target FPR < 0.1%, PSICOV, MI, and VI are the only methods with completely controlled FPR at all alignment sizes. MI_w, DI, and MI_j are controlled in alignments with fewer than 1000, 500, 250 sequences respectively. Together these results suggest that the DI, MI_w, MI_j, VI are sensitive to the amount of variation in the simulated alignments, while PSICOV and MI_{Hmin} are more robust to predicting fast and slowly evolving columns. However, MI_{Hmin}'s higher FPR suggests it is identifying coevolving residues that are not structurally close. Perhaps they may be part of an alternate network of evolutionarily important residues, for example "protein sectors [18]" that span more than one protein.

CoMap internally estimates P-values using a similar simulation approach. Nominal FPRs for CoMap methods, using their P-values directly, resemble those of the Information based approaches using the normal distribution as a null (twice to 20 times the target FPR). We conclude that it is very important for the evolutionary conservation of alignment columns in the bootstrap null distribution to closely match conservation levels in the observed data. Despite using currently accepted techniques for generating bootstrap distributions, we found that matching conservation levels this closely is challenging. This is an important problem for future research in the coevolution field.

1000 alignments were independently simulated for 8998 HisKA and RR sequences each.

First a phylogenetic tree for each alignment was built using FastTree (version 2.1.7 SSE3) with options `-gamma -nosupport -wag`.

The following steps were then automated in the simulate_tools pipeline:

1. Build profile HMM
2. Sample starting "root sequence" for simulation using first order Markov chain
3. Generate xml control file for Revolver
    - A. No tree scaling
    - B. Heterogeneous rates (alpha = 1, ncats = 9)
    - C. No indels
4. Run Revolver

An example command for simulating 1000 RR alignments:

```
runSimAli --tree RR.tree     \
    --outdir /path/to/output \
    --num\_sims 1000 JobNameRR RR.phy
```

From these simulated master alignments, sequences corresponding to the observed sub-alignments were extracted to create a total of 60,000 sub-alignments, each corresponding to one of the original 60 observed sub-alignments.

Structure visualization

The Vif complex 4N9F was rendered using the UCSF Chimera package (version 1.81) from the Computer Graphics Laboratory, University of California, San Francisco (supported by NIH P41 RR01081) [22].

Alternate theoretical null P_{gamma}

[9] derive the noncentral gamma distribution for a mutual information estimator sufficiently accurate for when the true MI <0.2 bits. The shape and scaling parameters depend on the number of observations (eg. number of sequences in alignment) and number of realizations of the two categorical variables (eg. number of different residues with non-zero probability in each alignment column), and the noncentrality parameter is used to specify "true MI" under the null hypothesis.

Coevolution methods partially evaluated

Two methods that can be considered as belonging to both Phylogenetic and Direct methods are CTMP [31] and Spidermonkey [23]. CTMP explicitly models coevolution using a continuous time markov process augmented to accomodate two proteins. Then to approximate a spatial dependency network for the alignment columns, it fits a maximum spanning tree to identify a core set of column pairs to investigate.

Spidermonkey [23] maps subsitutions to a phylogenetic tree, similarly to CoMap. However, instead of performing a pairwise analysis, it uses a Bayesian graphical model to learn the conditional dependencies between the alignment columns.

Because of technical difficulties with memory management and automation with these methods, we evaluated them on a subset of the data sets in our full benchmark (Figure S28;Figure S29). Spidermonkey appears to control the false positive rate (FPR) in the HisKA-RR data set for three alignments (N = 5, 500, 5000) at $P_{empirical} < \alpha$, and would appear to benefit from increasingly larger alignment in terms of power (TPR) and precision (PPV). We successfully ran CTMP on 10 alignments of N = 5, six of N = 50, and two of N = 250. CTMP does not appear to benefit from larger alignments as its FPR increases with N_{eff}/L, and PPV does not increase with N_{eff}/L.

Cross-Species Case Study 2: The interaction network of HIV1 and human proteins shows only weak evidence of coevolution across mammals

We sought to use inter-protein residue coevolution to refine a recently derived APMS protein-protein interaction network of the HIV1-human interactome [12]. This study detected human proteins that interact with each HIV1 protein, either via direct physical contact or as members of complexes. Specifically, we hoped

to use evidence of sequence coevolution to resolve direct versus indirect protein interactions amongst all human proteins measured to interact with each HIV1 protein.

For each protein in the HIV1 genome (nine polyproteins and an additional nine protease products), we computed a multiple sequence alignment with as many sequenced immunodeficiency viruses that infect mammals with sequenced genomes. We downloaded viral proteomes from Uniprot [30] and computationally processed the polyproteins for unannotated viruses. Uniprot IDs are in Additional File 6.

Similarly, we leveraged a set multiple alignments of each human protein (22,947 CCDS records) with the sequences of its orthologs from any mammal with a sequenced immunodeficiency virus [16]. Sequences are available at http://downloads.figshare.com/article/public/801140.

After pre-processing, there were a total of 425 joined alignments to analyze. However, even after filtering out the most conserved columns, the N_{eff}/L for the interactors is especially low. More than half of the interactors have lower N_{eff}/L than observed in either the HisKA-RR data set or Ovch32 Figure S38.

We created a null interaction network by permuting the interactions such that HIV1 and human proteins in the original interactome are mispaired. Null scores from the permuted network were used to calculate empirical P-values, $P^{(p)}{}_{empirical}$. The permuted network consists of 382 paired alignments. (alignments with fewer than 5 pairing sequences were discarded).

We ran five Information-based methods (MI, MI_w, MI_{Hmin}, MI_j, and VI) and a Direct method DI. The results with the empirical null distributions corroborate the bacterial benchmarks, establishing that without more sophisticated models and null distributions, coevolution problems with small N_{eff}/L will remain out

of reach (Figure S37).

Supplemental figures

Figure S1 HisKA-RR. Number of effective sequences (N_{eff}) versus number of sequence (N) in the 60 sub-sampled HisKA-RR alignments. Dashed line indicates the diagonal. Blue line indicates a linear fit with 95% confidence intervals in gray.

Figure S2 Ovch32 Number of effective sequences (N_{eff}) versus number of sequence (N) in the Ovch32 alignments. Dashed line indicates the diagonal. Blue line indicates a linear fit with 95% confidence intervals in gray.

Figure S3 Distribution of C distances in HisKA-RR interaction (PDB: 3DGE)

Figure S4 Distribution of C distances in Ovch32 interactions [21] (See supplemental file for PDB accessions)

Figure S5 Ovch32. Precision (PPV) versus N_{eff} at FPR $< 0.1\%$. Blue lines indicate a loess fit to each method, 95% confidence intervals are shown in gray.

Figure S6 Ovch32. Power (TPR) versus N_{eff} at FPR $< 5\%$. Blue lines indicate a loess fit to each method, 95% confidence intervals are shown in gray.

Figure S7 Ovch32. ϕ_{max} versus N_{eff}. Blue lines indicate a loess fit to each method, 95% confidence intervals are shown in gray.

Figure S8 HisKA-RR alt.. Power (TPR) vs N_{eff}/L at FPR $< 5\%$. A stricter definition of positives, defined experimentally in Haldimann et al. [10], Skerker et al. [28], and Laub and Goulian [15] is used. Blue lines indicate a loess fit to each method, 95% confidence intervals are shown in gray.

Figure S9 HisKA-RR alt.. Power (TPR) vs N_{eff}/L at FPR $< 0.1\%$. A

stricter definition of positives, defined experimentally in Haldimann et al. [10], Skerker et al. [28], and Laub and Goulian [15] is used. Blue lines indicate a loess fit to each method, 95% confidence intervals are shown in gray.

Figure S10 HisKA-RR alt.. Precision (PPV) vs N_{eff}/L at FPR < 0.1%. A stricter definition of positives, defined experimentally in Haldimann et al. [10], Skerker et al. [28], and Laub and Goulian [15] is used. Blue lines indicate a loess fit to each method, 95% confidence intervals are shown in gray.

Figure S11 Ovch32. Power (TPR) at FPR < 5% and Precision (PPV) at FPR < 0.1% versus N_{eff}/L. Blue lines indicate a loess fit to each method, 95% confidence intervals are shown in gray.

Figure S12 HisKA-RR. Nominal false positive rate (FPR) for target FPR 5%

Figure S13 Ovch32. Nominal false positive rate (FPR) for target FPR 0.1%

Figure S14 Ovch32. Nominal false positive rate (FPR) for target 5%

Figure S15 HisKA-RR. ϕ_{max}

Figure S16 HisKA-RR. F_{max}

Figure S17 HisKA-RR. Area under precision-recall curve

Figure S18 HisKA-RR. Area under ROC curve

Figure S19 Ovch32. ϕ_{max}

Figure S20 Ovch32. F_{max}

Figure S21 Ovch32. Area under precision-recall curve

Figure S22 Ovch32. Area under ROC curve

Figure S23 HisKA-RR. Median precision (PPV) at FPR <0.1% and median power (TPR) at FPR < 5% per rate categories of individual alignment columns.

Rate categories are defined as above- and below- median entropy for the HisKA and RR columns in each set of 10 alignments of equal size (number of sequences (N)).

Figure S24 Ovch32. Precision (PPV) versus the proportion of contacting pairs of residues in each interaction (i.e. contacting pairs divided by all pairs of residues) at FPR $< 0.1\%$

Figure S25 Ovch32. Precision (PPV) versus the proportion of contacting pairs of residues in each interaction (i.e. contacting pairs divided by all pairs of residues) at FPR $< 5\%$

Figure S26 Ovch32. False positive rate (FPR) versus the proportion of contacting pairs of residues in each interaction (i.e. contacting pairs divided by all pairs of residues) at $P < 0.05$

Figure S27 Ovch32. False positive rate (FPR) versus the proportion of contacting pairs of residues in each interaction (i.e. contacting pairs divided by all pairs of residues) at $P < 0.001$

Figure S28 HisKA-RR. The phylogenetic methods CTMP and Spidermonkey successfully ran on a subset of our alignments. Power (TPR) at FPR $< 5\%$ and precision (PPV) at FPR $< 0.1\%$. Select methods are included for comparison. Blue line indicates a linear fit with 95% confidence intervals in gray.

Figure S29 HisKA-RR. The phylogenetic method CTMP and Spidermonkey successfully ran on a subset of our alignments. Nominal false positive rate (FPR) at target FPR 0.1%. Select methods are included for comparison. Blue line indicates a linear fit with 95% confidence intervals in gray.

Figure S30 HisKA-RR. Quantile quantile plots of standardized coevolution scores are not always normally distributed. Scores are from 10 alignments with 5 sequences.

Figure S31 HisKA-RR. Quantile quantile plots of standardized coevolution scores are not always normally distributed. Scores are from 10 alignments with 500 sequences.

Figure S32 HisKA-RR. Quantile quantile plots of standardized coevolution scores are not always normally distributed. Scores are from 10 alignments with 5000 sequences.

Figure S33 HisKA-RR. $P_{boostrap}$ fails to control the FPR except for PSICOV at target FPR $<$ 5% in HisKA-RR alignments. Eliminating residue pairs with large simulation errors shows PSICOV and $\mathrm{MI}_{\mathrm{Hmin}}$ are most robust to variation at individual sites. See Misc. Abbreviations and Table S1 for abbreviations.

Figure S34 Vif. Power (TPR), precision (PPV), and false positive rate (FPR) for predicting viral protein Vif residues (not pairs) essential for interacting with its host target A3G at $P_{empirical} < \alpha$ thresholds that maximize PPV for each coevolution method. Residues defined as positive are taken from previous functional mutation studies in Table S3. See Abbreviations and Table S1 for abbreviations.

Figure S35 Vif. Power (TPR), precision (PPV), and false positive rate (FPR) for predicting viral protein Vif residues (not pairs) essential for interacting with its host target A3G at $P_{empirical} < \alpha$ thresholds that maximize PPV for each coevolution method. Residues defined as positive are taken from previous functional mutation studies in Table S3. See Abbreviations and Table S1 for abbreviations.Vifcrit PPVoptbars

Figure S36 Residues (red) on viral protein Vif (light blue) that are predicted to coevolve with it host target A3G (structure unknown). Cofactors are shown in gray. Predictions are made at a threshold that maximizes precision (PPV) using **A** known essential residues (Table S3) using **B-D** MI, DI, $\mathrm{CMP}_{\mathrm{vol}}$ respectively.

Figure S37 HIV1-human Distinguishing HIV1-human interactors from a protein pairs in a permuted network is difficult with small N_{eff}/L. ϕ_{max} across a the number of predicted coevolving column-pairs per protein-pair versus $P\hat{\ }p_{empirical}$ threshold for making column-pair predictions. Blue line indicates a linear fit with 95% confidence intervals in gray.

Figure S38 HIV1-human N_{eff}/L distribution of alignments in HIV1-human interactors The minimum N_{eff}/L seen in the HisKA-RR (red) and Ovch32 (orange) data sets is marked.

References

[1] W. Cai, J. Pei, and N. V. Grishin. “Reconstruction of ancestral protein sequences and its applications”. eng. In: *BMC evolutionary biology* 4 (2004), p. 33. DOI: 10.1186/1471-2148-4-33.

[2] G. Chen et al. “A patch of positively charged amino acids surrounding the human immunodeficiency virus type 1 Vif SLVx4Yx9Y motif influences its interaction with APOBEC3G”. eng. In: *Journal of virology* 83.17 (2009), pp. 8674–82. DOI: 10.1128/JVI.00653-09.

[3] S. Cocco, R. Monasson, and M. Weigt. “From principal component to direct coupling analysis of coevolution in proteins: low-eigenvalue modes are needed for structure prediction”. eng. In: *PLoS computational biology* 9.8 (2013), e1003176. DOI: 10.1371/journal.pcbi.1003176.

[4] S. D. Dunn, L. M. Wahl, and G. B. Gloor. “Mutual information without the influence of phylogeny or entropy dramatically improves residue contact prediction”. eng. In: *Bioinformatics* 24.3 (2008), pp. 333–40. DOI: 10.1093/bioinformatics/btm604.

[5] J. Dutheil and N. Galtier. “Detecting groups of coevolving positions in a molecule: a clustering approach”. In: *BMC Evol Biol* 7 (2007), p. 242. DOI: 10.1186/1471-2148-7-242.

[6] J. Dutheil et al. “A model-based approach for detecting coevolving positions in a molecule”. eng. In: *Molecular biology and evolution* 22.9 (2005), pp. 1919–28. DOI: 10.1093/molbev/msi183.

[7] S. R. Eddy. “Accelerated Profile HMM Searches”. eng. In: *PLoS computational biology* 7.10 (2011), e1002195. DOI: 10.1371/journal.pcbi.1002195.

[8] M. Ekeberg et al. “Improved contact prediction in proteins: using pseudolikelihoods to infer Potts models”. eng. In: *Physical review. E, Statistical, nonlinear, and soft matter physics* 87.1 (2013), p. 012707. URL: http://www.ncbi.nlm.nih.gov/pubmed/23410359.

[9] Bernhard Goebel et al. “An approximation to the distribution of finite sample size mutual information estimates”. In: *Communications, 2005. ICC 2005. 2005 IEEE International Conference on*. Vol. 2. IEEE, 2005, pp. 1102–1106. URL: https://ieeexplore.ieee.org/ielx5/9996/32110/01494518.pdf (visited on 02/12/2015).

[10] A. Haldimann et al. “Altered recognition mutants of the response regulator PhoB: a new genetic strategy for studying protein-protein interactions”. eng. In: *Proceedings of the National Academy of Sciences of the United States of America* 93.25 (1996), pp. 14361–6. URL: http://www.ncbi.nlm.nih.gov/pubmed/8962056.

[11] Z. He et al. “Characterization of conserved motifs in HIV-1 Vif required for APOBEC3G and APOBEC3F interaction”. eng. In: *Journal of molecular biology* 381.4 (2008), pp. 1000–11. DOI: 10.1016/j.jmb.2008.06.061.

[12] S. Jager et al. “Global landscape of HIV-human protein complexes”. eng. In: *Nature* 481.7381 (2012), pp. 365–70. DOI: 10.1038/nature10719.

[13] D. T. Jones et al. “PSICOV: precise structural contact prediction using sparse inverse covariance estimation on large multiple sequence alignments”. eng. In: *Bioinformatics* 28.2 (2012), pp. 184–90. DOI: 10.1093/bioinformatics/btr638.

[14] T. Koestler, A. von Haeseler, and I. Ebersberger. “REvolver: modeling sequence evolution under domain constraints”. In: *Mol Biol Evol* 29.9 (2012), pp. 2133–45. DOI: 10.1093/molbev/mss078.

[15] M. T. Laub and M. Goulian. "Specificity in two-component signal transduction pathways". eng. In: *Annual review of genetics* 41 (2007), pp. 121–45. DOI: 10.1146/annurev.genet.41.042007.170548.

[16] M. C. Maher and R. D. Hernandez. "Rock, Paper, Scissors: Harnessing Complementarity in Ortholog Detection Methods Improves Comparative Genomic Inference". en. In: *G3: Genes|Genomes|Genetics* 5.4 (Apr. 2015), pp. 629–638. ISSN: 2160-1836. DOI: 10.1534/g3.115.017095. (Visited on 06/07/2015).

[17] W. Mao et al. "Comparative study of the effectiveness and limitations of current methods for detecting sequence coevolution". en. In: *Bioinformatics* (Feb. 2015). ISSN: 1367-4803, 1460-2059. DOI: 10.1093/bioinformatics/btv103. (Visited on 04/03/2015).

[18] R. N. McLaughlin Jr. et al. "The spatial architecture of protein function and adaptation". eng. In: *Nature* 491.7422 (2012), pp. 138–42. DOI: 10.1038/nature11500.

[19] Marina Meila. "Comparing clusterings–an information based distance". In: *Journal of Multivariate Analysis* 98.5 (2007), pp. 873–895. ISSN: 0047-259X. DOI: http://dx.doi.org/10.1016/j.jmva.2006.11.013.

[20] F. Morcos et al. "Direct-coupling analysis of residue coevolution captures native contacts across many protein families". eng. In: *Proceedings of the National Academy of Sciences of the United States of America* 108.49 (2011), E1293–301. DOI: 10.1073/pnas.1111471108.

[21] Ovchinnikov S, Kamisetty H, and Baker D. *Data from: Robust and accurate prediction of residue-residue interactions across protein interfaces using evolutionary information*. May 2014. URL: http://dx.doi.org/10.5061/dryad.s00vr.

[22] E. F. Pettersen et al. "UCSF Chimera–a visualization system for exploratory research and analysis". eng. In: *Journal of computational chemistry* 25.13 (2004), pp. 1605–12. DOI: 10.1002/jcc.20084.

[23] A. F. Y. Poon et al. "Spidermonkey: rapid detection of co-evolving sites using Bayesian graphical models". en. In: *Bioinformatics* 24.17 (Sept. 2008), pp. 1949–1950. ISSN: 1367-4803, 1460-2059. DOI: 10.1093/bioinformatics/btn313. (Visited on 04/29/2015).

[24] M. N. Price, P. S. Dehal, and A. P. Arkin. "FastTree 2–approximately maximum-likelihood trees for large alignments". eng. In: *PloS one* 5.3 (2010), e9490. DOI: 10.1371/journal.pone.0009490.

[25] R. A. Russell and V. K. Pathak. "Identification of two distinct human immunodeficiency virus type 1 Vif determinants critical for interactions with human APOBEC3G and APOBEC3F". eng. In: *Journal of virology* 81.15 (2007), pp. 8201–10. DOI: 10.1128/JVI.00395-07.

[26] R. A. Russell et al. "Distinct domains within APOBEC3G and APOBEC3F interact with separate regions of human immunodeficiency virus type 1 Vif". eng. In: *Journal of virology* 83.4 (2009), pp. 1992–2003. DOI: 10.1128/JVI.01621-08.

[27] Claude Elwood Shannon. "A mathematical theory of communication". In: *The Bell System Technical Journal* 27 (Oct. 1948), pp. 379–423, 623–656. URL: http://cm.bell-labs.com/cm/ms/what/shannonday/shannon1948.pdf (visited on 02/11/2015).

[28] J. M. Skerker et al. "Rewiring the specificity of two-component signal transduction systems". eng. In: *Cell* 133.6 (2008), pp. 1043–54. DOI: 10.1016/j.cell.2008.04.040.

[29] A. Stamatakis. “RAxML-VI-HPC: maximum likelihood-based phylogenetic analyses with thousands of taxa and mixed models”. eng. In: *Bioinformatics* 22.21 (2006), pp. 2688–90. DOI: 10.1093/bioinformatics/btl446.

[30] The UniProt Consortium. “UniProt: a hub for protein information”. en. In: *Nucleic Acids Research* 43.D1 (Jan. 2015), pp. D204–D212. ISSN: 0305-1048, 1362-4962. DOI: 10.1093/nar/gku989. (Visited on 04/14/2015).

[31] C. H. Yeang and D. Haussler. “Detecting coevolution in and among protein domains”. eng. In: *PLoS computational biology* 3.11 (2007), e211. DOI: 10.1371/journal.pcbi.0030211.

[32] H. Zhang et al. “Human immunodeficiency virus type 1 Vif protein is an integral component of an mRNP complex of viral RNA and could be involved in the viral RNA folding and packaging process”. eng. In: *Journal of virology* 74.18 (2000), pp. 8252–61. URL: http://www.ncbi.nlm.nih.gov/pubmed/10954522.

[33] L. Zhang et al. “Function analysis of sequences in human APOBEC3G involved in Vif-mediated degradation”. eng. In: *Virology* 370.1 (2008), pp. 113–21. DOI: 10.1016/j.virol.2007.08.027.

Table S1: List of methods benchmarked

	Method	APC	Re-weighting	Reference	Software package
Information-based	MI	No	None	[27, 4]	infCalc
	VI			[19]	
	MI_{j}			[4]	
	MI_{Hmin}				
	MI_{w}		seq %id	[20]	DCA
Direct	DI	Yes	seq %id, pseudocount		
	DI_{256}			[3]	Code S1 in [3]
	DI_{32}				
	DI_{plm}		seq %id	[8]	plmDCA
	PSICOV		Blosum, pseudocount	[13]	PSICOV
Phylogenetic	CMP_{cor}	No	Downsampling	[6]	CoMap
	CMP_{chg}			[5]	
	CMP_{vol}				
	CMP_{pol}				

Coevolution methods benchmarked fall into three categories. Information-based methods: MI: mutual information [27], VI: variation of information [19], MI_{j}: MI divided by alignment column-pair entropy, MI_{Hmin}: MI divided by minimum column entropy [4], MI_{w}: MI with adjusted amino acid probabilities. Direct methods: DI: direct information—MI with re-estimated joint probabilities [20], DI_{256}, DI_{32}: DI using Hopfield-Potts for dimensional reduction (256 and 32 patterns respectively) [3], DI_{plm}: Frobenius norm of coupling matrices in 21-state Potts model using pseudolikelihood maximization [8], PSICOV: sparse inverse covariance estimation [13]. Phylogenetic methods: CoMap *P*-values for four analyses CMP_{cor}: substitution correlation analysis [6], CMP_{pol} for polarity compensation, CMP_{chg} for charge compensation, CMP_{vol} for volume compensation [5].

Table S2: Confusion matrix.

	Prediction	
C_β distance	Coevolving	Not coevolving
< 8Å	TP	FN
≥ 8Å	FP	TN

Table S3: Important residues for the Vif-A3G interaction

	Position	Notes
Vif	21-23,26	A3G-specific
	30	
	40-44	
	55-72	A3G and A3F
A3G	121-149	essential for Vif-binding

HIV1 Vif [2, 25, 32, 11]. Human A3G [33, 26].

Table S4: Versions and sources of coevolution methods benchmarked

	Method	Software package	Version	URL
Information-based	MI	infCalc	v0.1.2	https://github.com/aavilahe/infcalc
	VI			
	MI_j			
	MI_{Hmin}			
	MI_w	DCA	”2011/12”	http://dca.ucsd.edu/DCA/DCA.html
Direct	DI			
	DI_{256}	Code S1 in [3]	”2013”	http://doi.org/10.1371/journal.pcbi.1003176.s002
	DI_{32}			
	DI_{plm}	plmDCA	symmetric_v2	http://plmdca.csc.kth.se/
	PSICOV	PSICOV	V1.09	http://bioinfadmin.cs.ucl.ac.uk/downloads/PSICOV/
Phylogenetic	CMP_{cor}	CoMap	1.5.1b5	http://home.gna.org/comap/doc/html/index.html
	CMP_{chg}			
	CMP_{vol}			
	CMP_{pol}			
