# Supplementary figures and images for: Coevolutionary analyses require phylogenetically deep alignments and better null models to accurately detect inter-protein contacts within and between species
Source: BMC Bioinformatics. 2015 Aug 25;16:268. doi: 10.1186/s12859-015-0677-y (PMC4549020; doi:10.1186/s12859-015-0677-y)

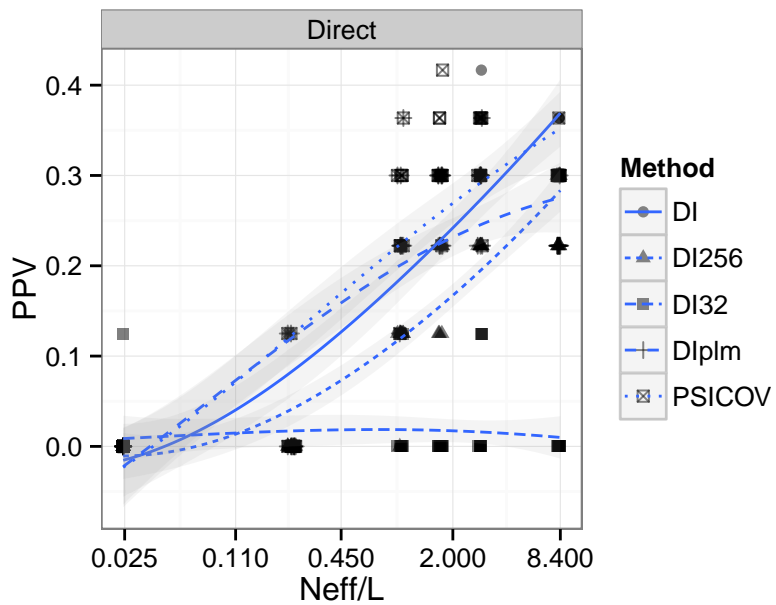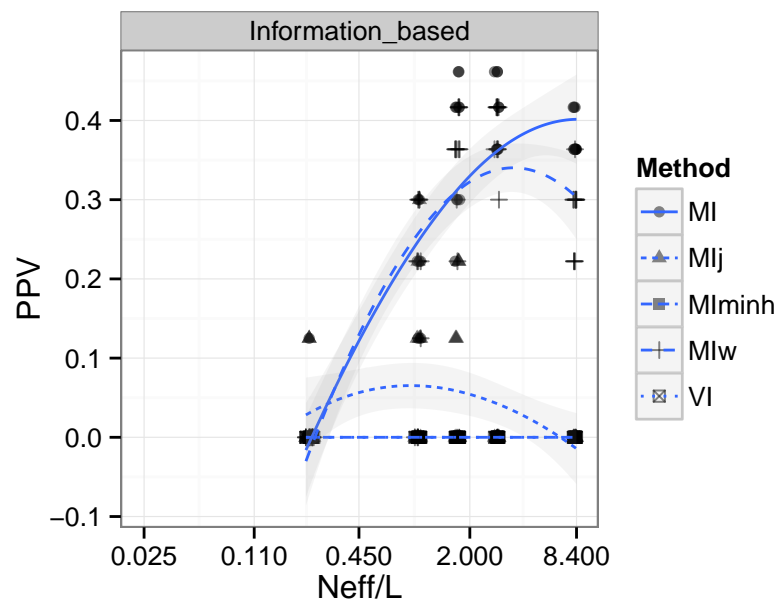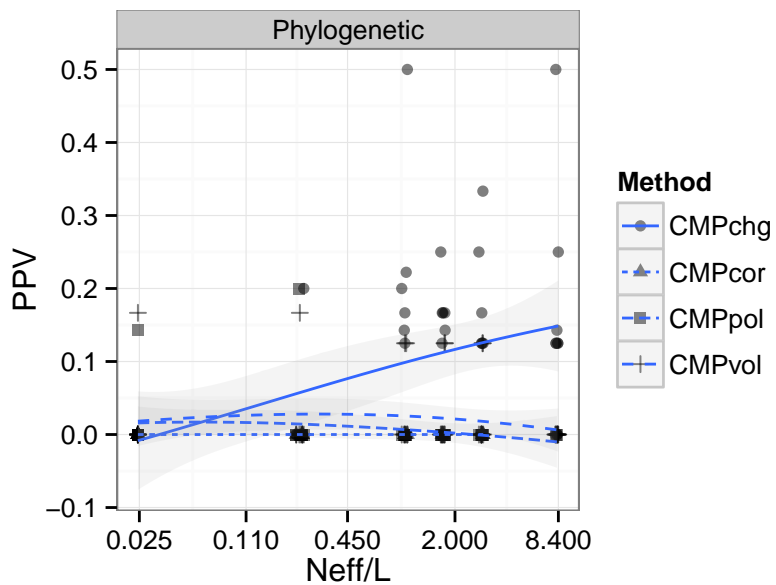

Supplement: Additional file 13 — Figure S1. HisKA-RR. Number of effective sequences (N eff) versus number of sequence (N) in the 60 sub-sampled HisKA-RR alignments. Dashed line indicates the diagonal. Blue line indicates a linear fit with 95 % confidence intervals in gray. Figure S2. Ovch32. Number of effective sequences (N eff) versus number of sequence (N) in the Ovch32 alignments. Dashed line indicates the diagonal. Blue line indicates a linear fit with 95 % confidence intervals in gray. Figure S3. Distribution of C β distances in HisKA-RR interaction (PDB: 3DGE). Figure S4. Distribution of C β distances in Ovch32 interactions [67] (See supplemental file for PDB accessions). Figure S5. Ovch32. Precision (PPV) versus Neff at FPR < 0.1 %. Blue lines indicate a loess fit to each method, 95 % confidence intervals are shown in gray. Figure S6. Ovch32. Power (TPR) versus Neff at FPR < 5 %. Blue lines indicate a loess fit to each method, 95 % confidence intervals are shown in gray. Figure S7. Ovch32. ϕ max versus Neff. Blue lines indicate a loess fit to each method, 95 % confidence intervals are shown in gray. Figure S8. HisKA-RR alt.. Power (TPR) vs Neff/L at FPR < 5 %. A stricter definition of positives, defined experimentally in [46–48] is used. Blue lines indicate a loess fit to each method, 95 % confidence intervals are shown in gray. Figure S9. HisKA-RR alt.. Power (TPR) vs Neff/L at FPR < 0.1 %. A stricter definition of positives, defined experimentally in [46–48] is used. Blue lines indicate a loess fit to each method, 95 % confidence intervals are shown in gray. Figure S10. HisKA-RR alt.. Precision (PPV) vs Neff/L at FPR < 0.1 %. A stricter definition of positives, defined experimentally in [46–48] is used. Blue lines indicate a loess fit to each method, 95 % confidence intervals are shown in gray. Figure S11. Ovch32. Power (TPR) at FPR < 5 % and Precision (PPV) at FPR < 0.1 % versus Neff/L. Blue lines indicate a loess fit to each method, 95 % confidence intervals are shown in gray. Figure S12 [file 12859_2015_677_MOESM13_ESM.zip › 12859_2015_677_add13/Fig_S10_PPV_Neff_per_col_at_0.001.pdf]

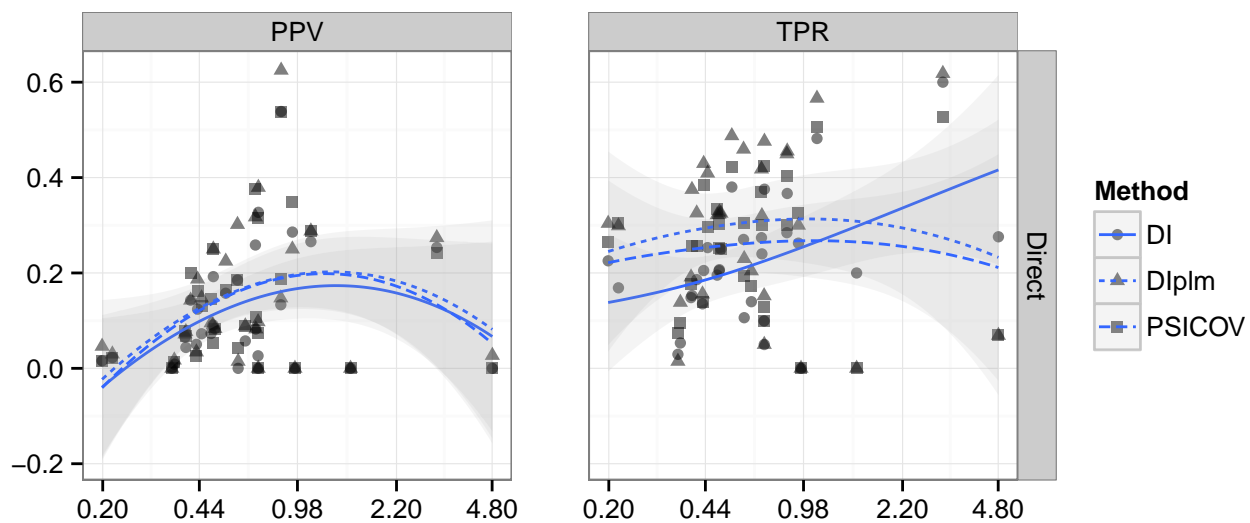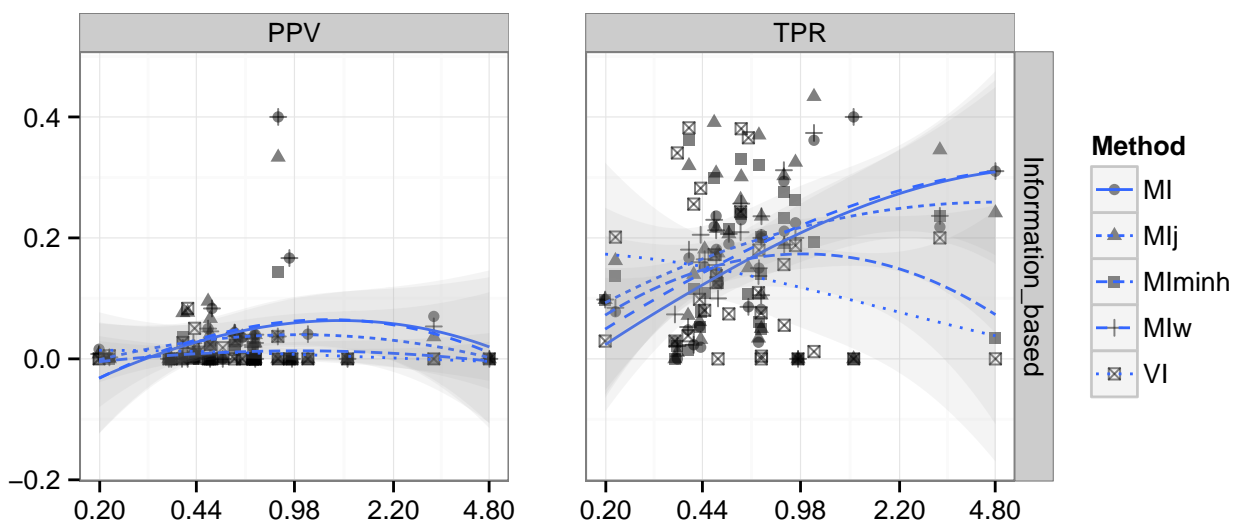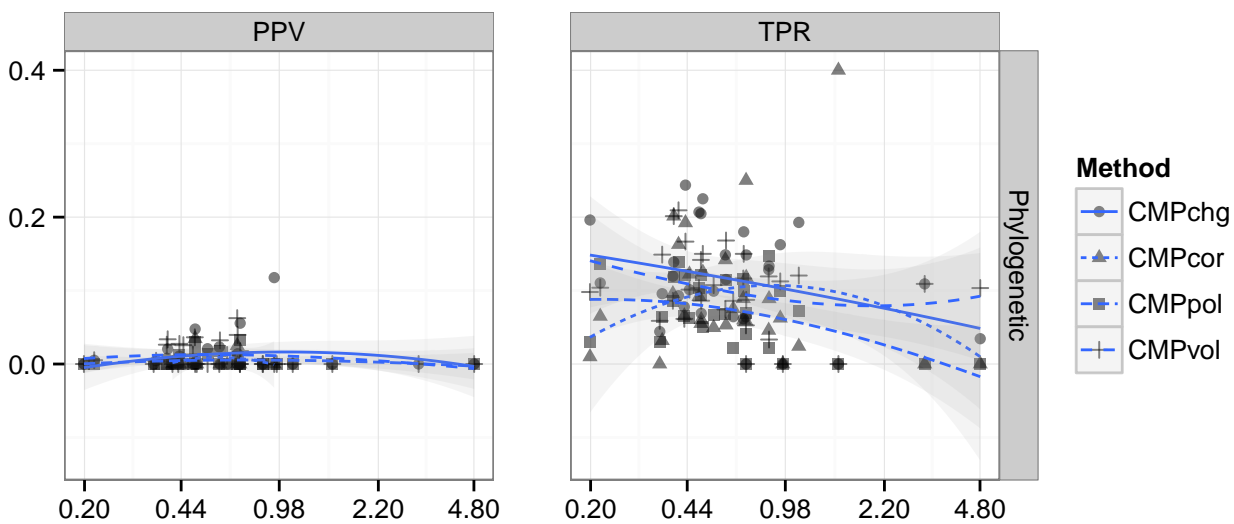

Neff/L

Supplement: Additional file 13 — Figure S1. HisKA-RR. Number of effective sequences (N eff) versus number of sequence (N) in the 60 sub-sampled HisKA-RR alignments. Dashed line indicates the diagonal. Blue line indicates a linear fit with 95 % confidence intervals in gray. Figure S2. Ovch32. Number of effective sequences (N eff) versus number of sequence (N) in the Ovch32 alignments. Dashed line indicates the diagonal. Blue line indicates a linear fit with 95 % confidence intervals in gray. Figure S3. Distribution of C β distances in HisKA-RR interaction (PDB: 3DGE). Figure S4. Distribution of C β distances in Ovch32 interactions [67] (See supplemental file for PDB accessions). Figure S5. Ovch32. Precision (PPV) versus Neff at FPR < 0.1 %. Blue lines indicate a loess fit to each method, 95 % confidence intervals are shown in gray. Figure S6. Ovch32. Power (TPR) versus Neff at FPR < 5 %. Blue lines indicate a loess fit to each method, 95 % confidence intervals are shown in gray. Figure S7. Ovch32. ϕ max versus Neff. Blue lines indicate a loess fit to each method, 95 % confidence intervals are shown in gray. Figure S8. HisKA-RR alt.. Power (TPR) vs Neff/L at FPR < 5 %. A stricter definition of positives, defined experimentally in [46–48] is used. Blue lines indicate a loess fit to each method, 95 % confidence intervals are shown in gray. Figure S9. HisKA-RR alt.. Power (TPR) vs Neff/L at FPR < 0.1 %. A stricter definition of positives, defined experimentally in [46–48] is used. Blue lines indicate a loess fit to each method, 95 % confidence intervals are shown in gray. Figure S10. HisKA-RR alt.. Precision (PPV) vs Neff/L at FPR < 0.1 %. A stricter definition of positives, defined experimentally in [46–48] is used. Blue lines indicate a loess fit to each method, 95 % confidence intervals are shown in gray. Figure S11. Ovch32. Power (TPR) at FPR < 5 % and Precision (PPV) at FPR < 0.1 % versus Neff/L. Blue lines indicate a loess fit to each method, 95 % confidence intervals are shown in gray. Figure S12 [file 12859_2015_677_MOESM13_ESM.zip › 12859_2015_677_add13/Fig_S11_TPR5_PPV01_Neff_per_col.pdf]

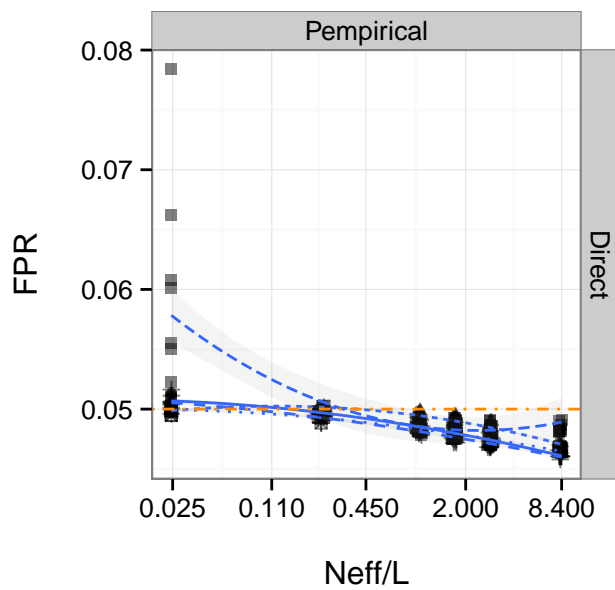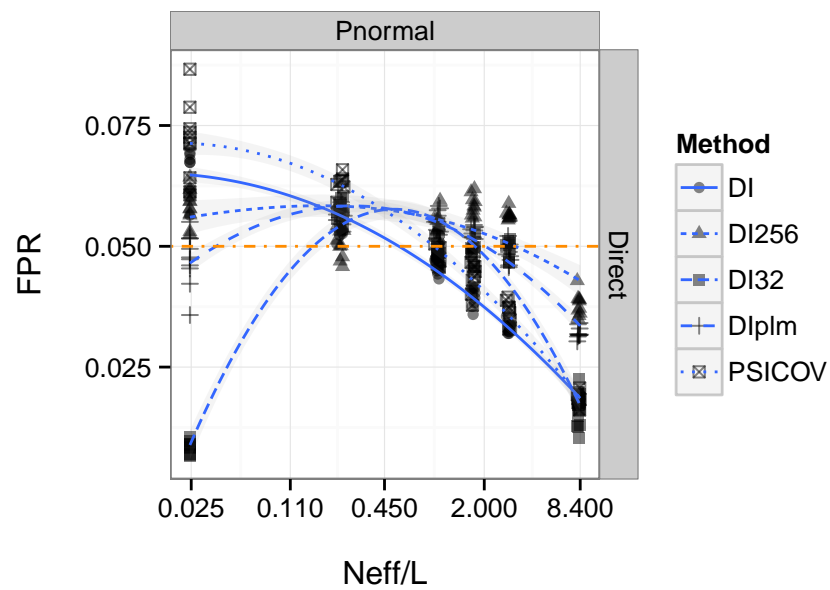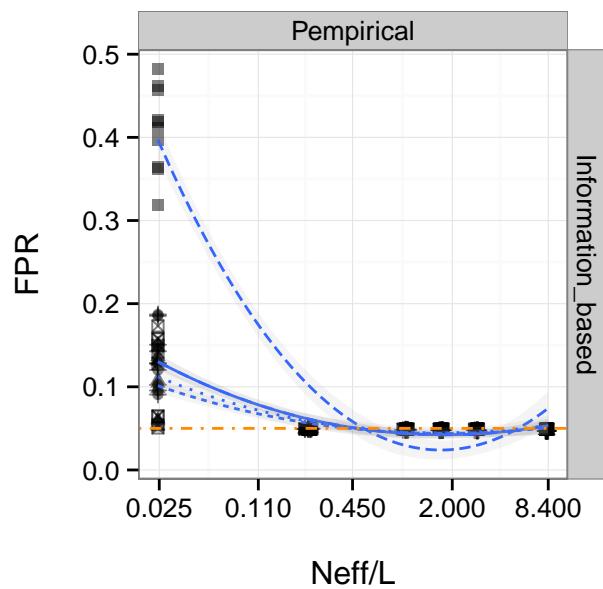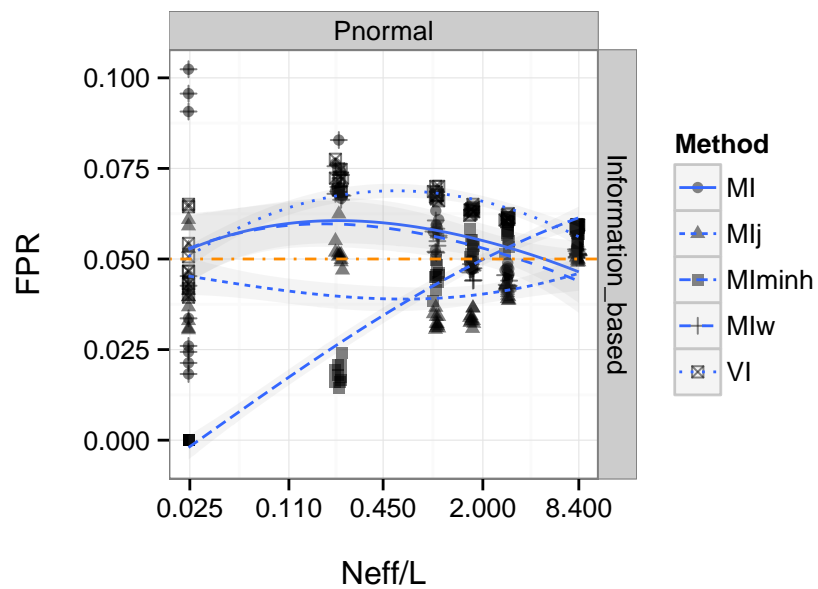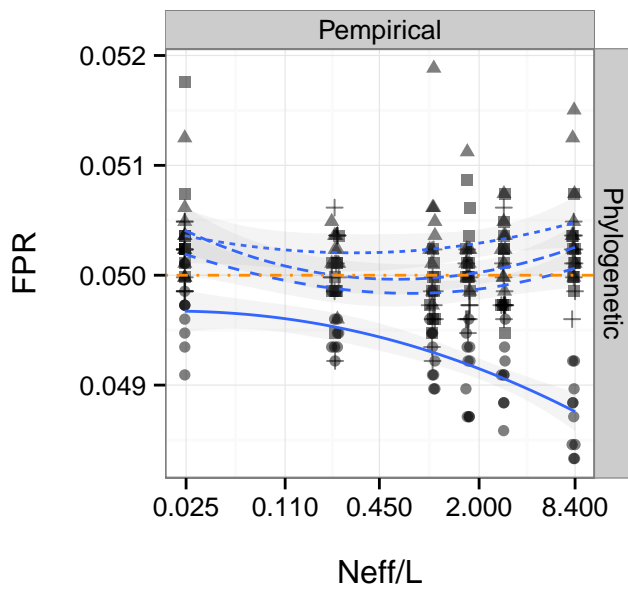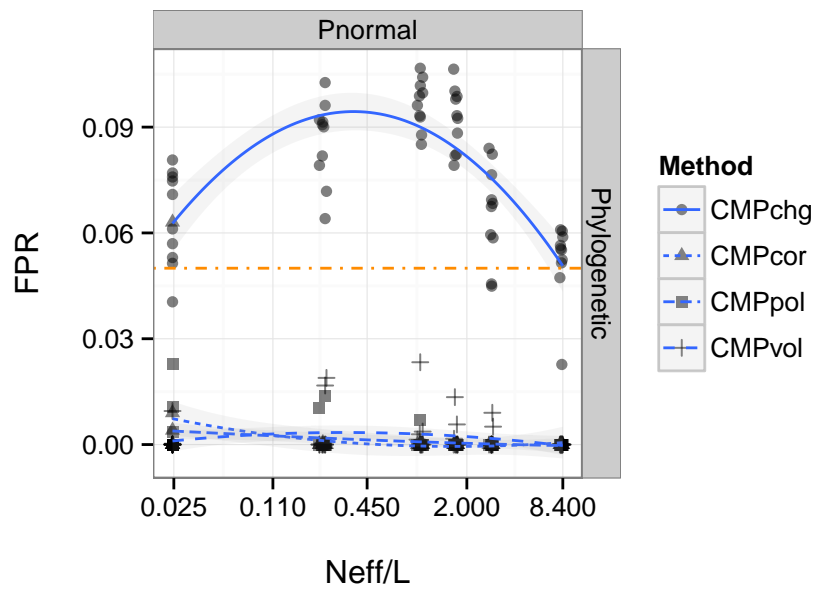

Supplement: Additional file 13 — Figure S1. HisKA-RR. Number of effective sequences (N eff) versus number of sequence (N) in the 60 sub-sampled HisKA-RR alignments. Dashed line indicates the diagonal. Blue line indicates a linear fit with 95 % confidence intervals in gray. Figure S2. Ovch32. Number of effective sequences (N eff) versus number of sequence (N) in the Ovch32 alignments. Dashed line indicates the diagonal. Blue line indicates a linear fit with 95 % confidence intervals in gray. Figure S3. Distribution of C β distances in HisKA-RR interaction (PDB: 3DGE). Figure S4. Distribution of C β distances in Ovch32 interactions [67] (See supplemental file for PDB accessions). Figure S5. Ovch32. Precision (PPV) versus Neff at FPR < 0.1 %. Blue lines indicate a loess fit to each method, 95 % confidence intervals are shown in gray. Figure S6. Ovch32. Power (TPR) versus Neff at FPR < 5 %. Blue lines indicate a loess fit to each method, 95 % confidence intervals are shown in gray. Figure S7. Ovch32. ϕ max versus Neff. Blue lines indicate a loess fit to each method, 95 % confidence intervals are shown in gray. Figure S8. HisKA-RR alt.. Power (TPR) vs Neff/L at FPR < 5 %. A stricter definition of positives, defined experimentally in [46–48] is used. Blue lines indicate a loess fit to each method, 95 % confidence intervals are shown in gray. Figure S9. HisKA-RR alt.. Power (TPR) vs Neff/L at FPR < 0.1 %. A stricter definition of positives, defined experimentally in [46–48] is used. Blue lines indicate a loess fit to each method, 95 % confidence intervals are shown in gray. Figure S10. HisKA-RR alt.. Precision (PPV) vs Neff/L at FPR < 0.1 %. A stricter definition of positives, defined experimentally in [46–48] is used. Blue lines indicate a loess fit to each method, 95 % confidence intervals are shown in gray. Figure S11. Ovch32. Power (TPR) at FPR < 5 % and Precision (PPV) at FPR < 0.1 % versus Neff/L. Blue lines indicate a loess fit to each method, 95 % confidence intervals are shown in gray. Figure S12 [file 12859_2015_677_MOESM13_ESM.zip › 12859_2015_677_add13/Fig_S12_FPR_0.05_Neff_per_col.pdf]

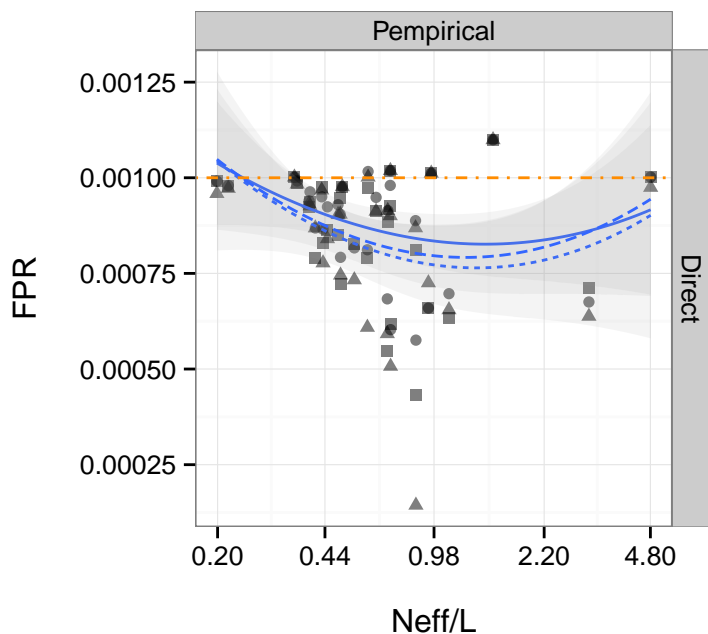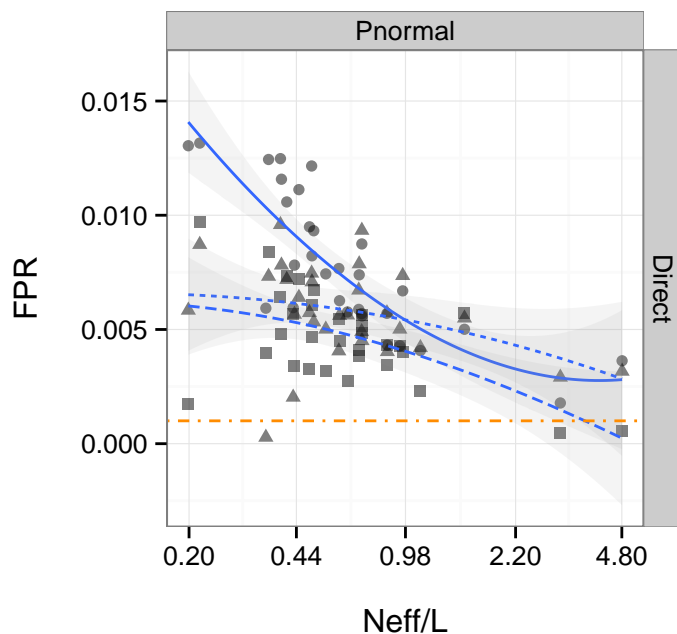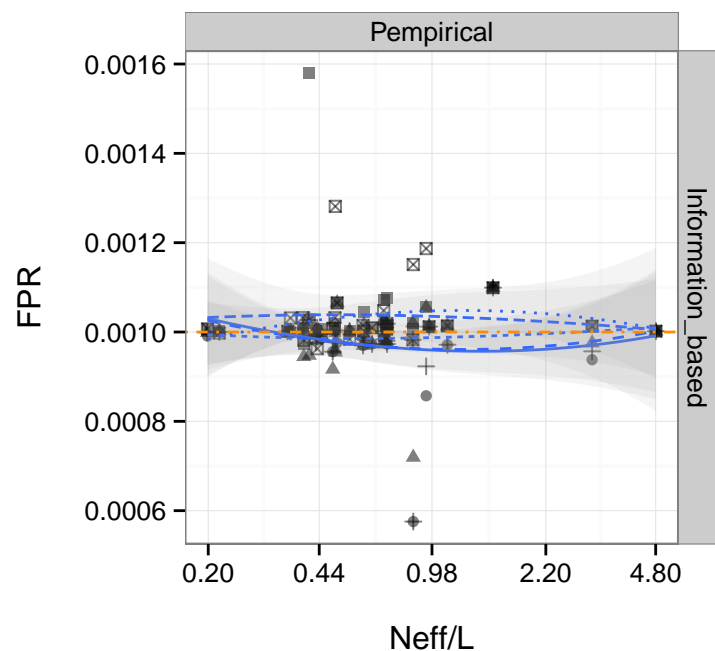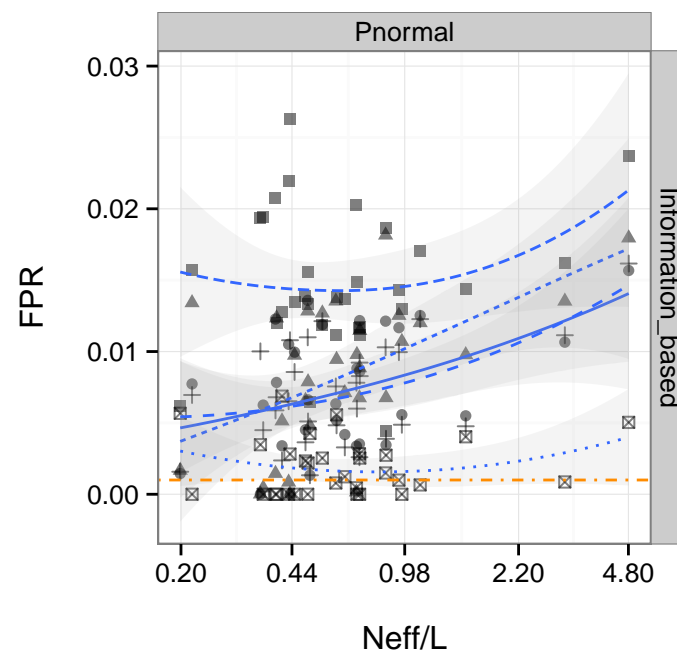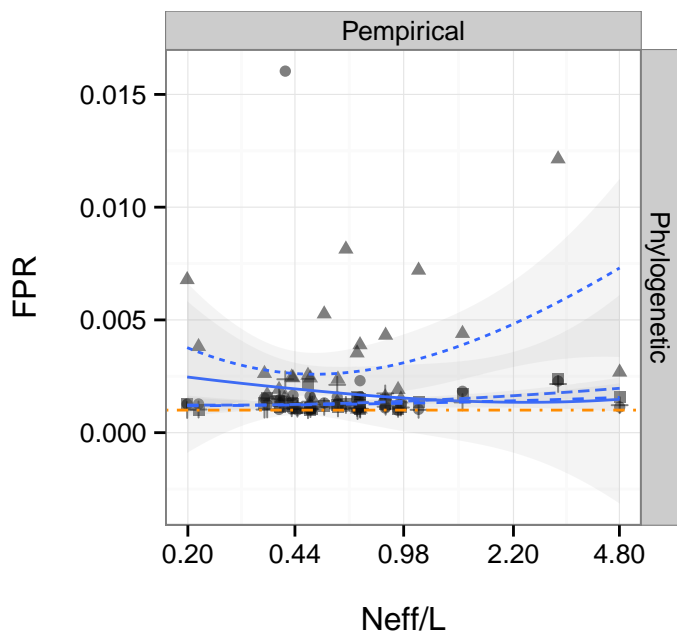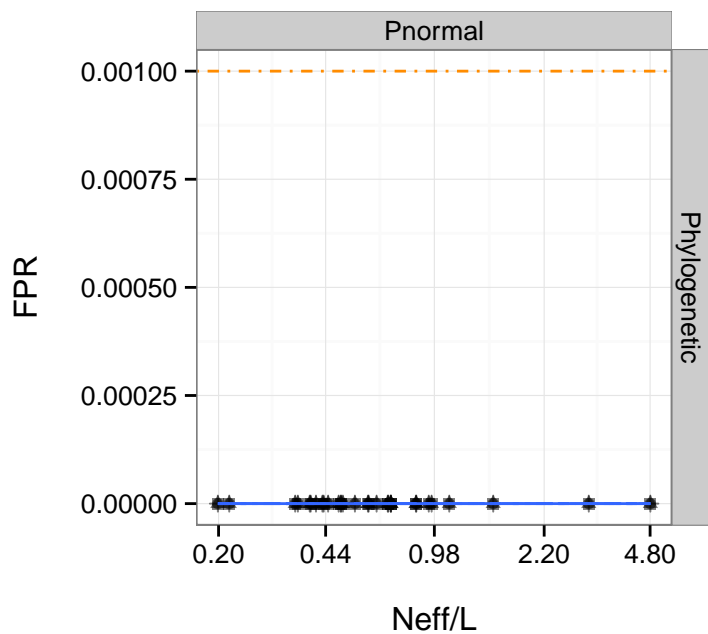

Supplement: Additional file 13 — Figure S1. HisKA-RR. Number of effective sequences (N eff) versus number of sequence (N) in the 60 sub-sampled HisKA-RR alignments. Dashed line indicates the diagonal. Blue line indicates a linear fit with 95 % confidence intervals in gray. Figure S2. Ovch32. Number of effective sequences (N eff) versus number of sequence (N) in the Ovch32 alignments. Dashed line indicates the diagonal. Blue line indicates a linear fit with 95 % confidence intervals in gray. Figure S3. Distribution of C β distances in HisKA-RR interaction (PDB: 3DGE). Figure S4. Distribution of C β distances in Ovch32 interactions [67] (See supplemental file for PDB accessions). Figure S5. Ovch32. Precision (PPV) versus Neff at FPR < 0.1 %. Blue lines indicate a loess fit to each method, 95 % confidence intervals are shown in gray. Figure S6. Ovch32. Power (TPR) versus Neff at FPR < 5 %. Blue lines indicate a loess fit to each method, 95 % confidence intervals are shown in gray. Figure S7. Ovch32. ϕ max versus Neff. Blue lines indicate a loess fit to each method, 95 % confidence intervals are shown in gray. Figure S8. HisKA-RR alt.. Power (TPR) vs Neff/L at FPR < 5 %. A stricter definition of positives, defined experimentally in [46–48] is used. Blue lines indicate a loess fit to each method, 95 % confidence intervals are shown in gray. Figure S9. HisKA-RR alt.. Power (TPR) vs Neff/L at FPR < 0.1 %. A stricter definition of positives, defined experimentally in [46–48] is used. Blue lines indicate a loess fit to each method, 95 % confidence intervals are shown in gray. Figure S10. HisKA-RR alt.. Precision (PPV) vs Neff/L at FPR < 0.1 %. A stricter definition of positives, defined experimentally in [46–48] is used. Blue lines indicate a loess fit to each method, 95 % confidence intervals are shown in gray. Figure S11. Ovch32. Power (TPR) at FPR < 5 % and Precision (PPV) at FPR < 0.1 % versus Neff/L. Blue lines indicate a loess fit to each method, 95 % confidence intervals are shown in gray. Figure S12 [file 12859_2015_677_MOESM13_ESM.zip › 12859_2015_677_add13/Fig_S13_FPR_0.001_Neff_per_col.pdf]

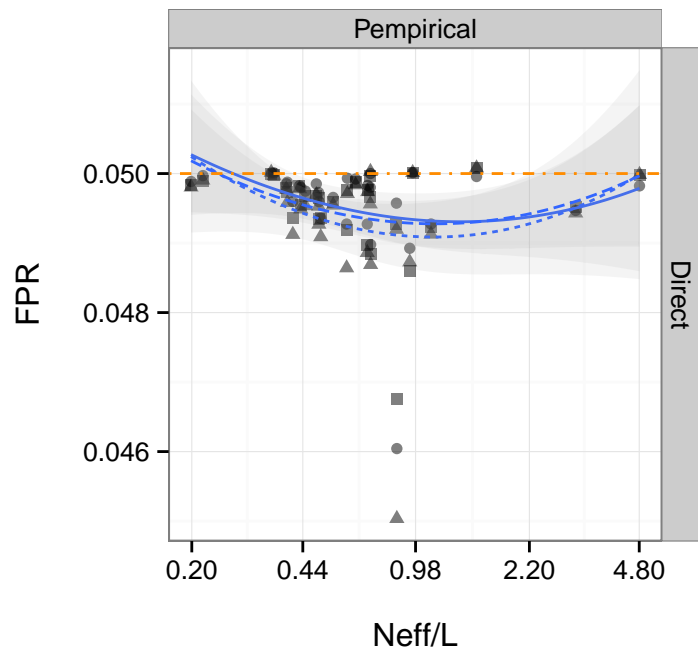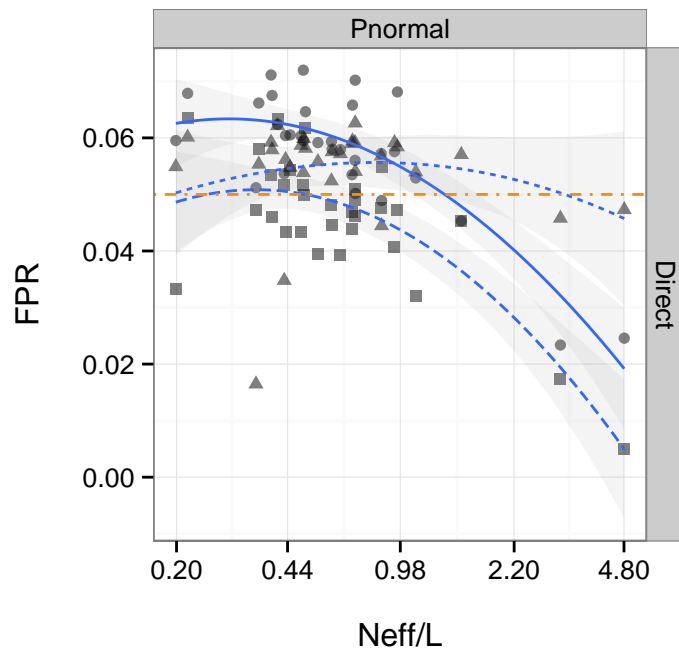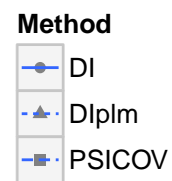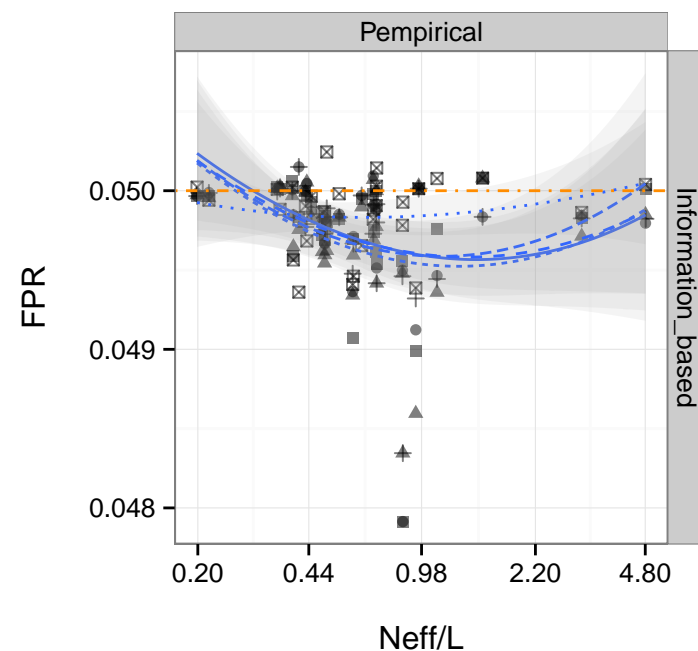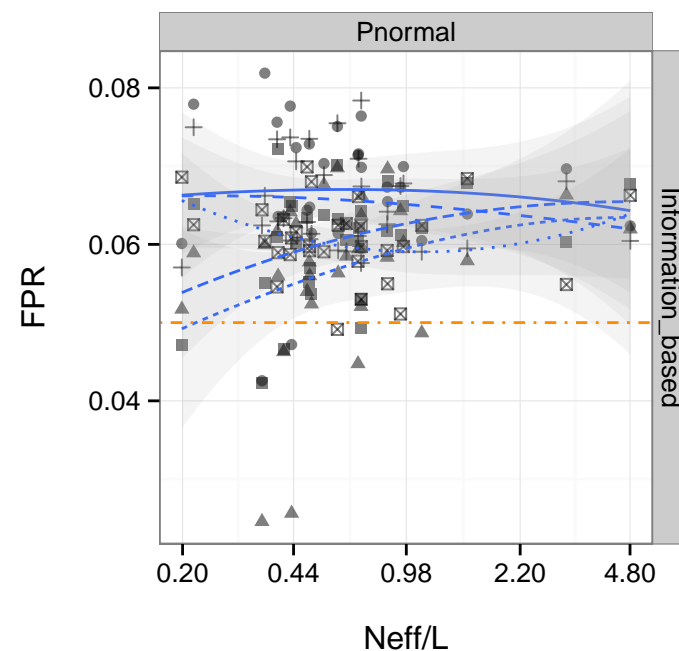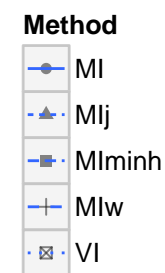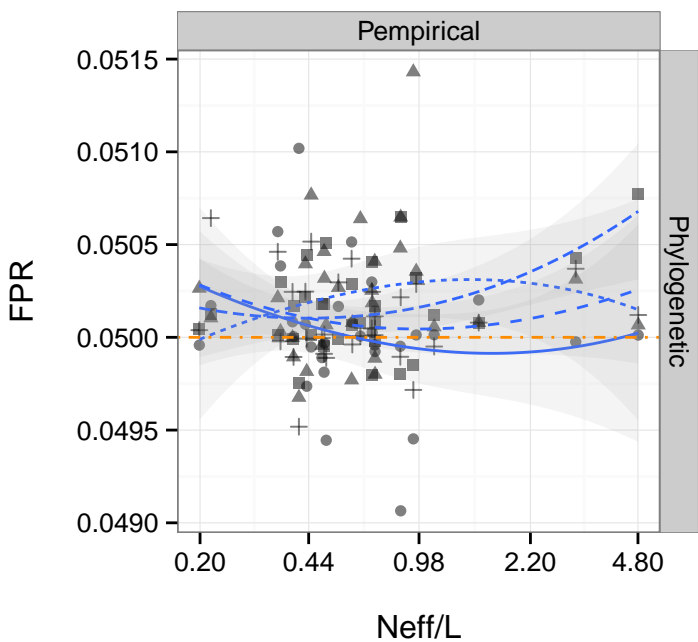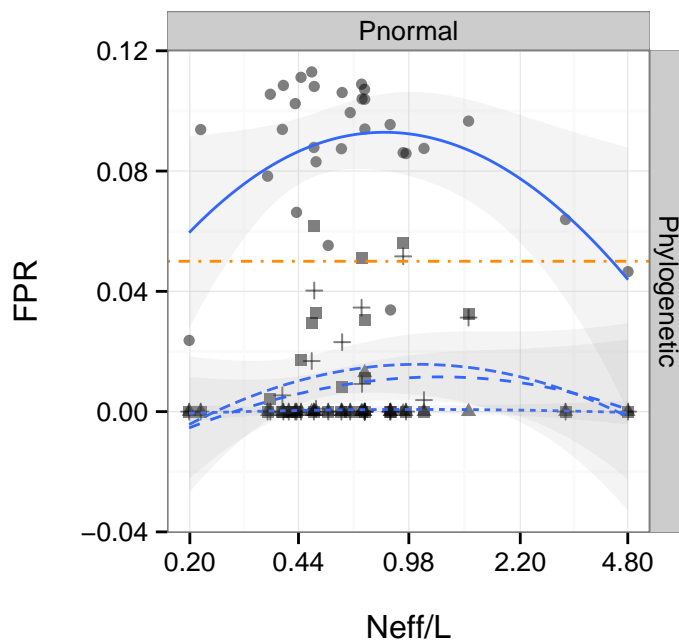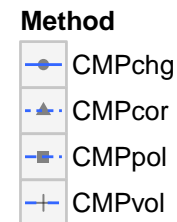

Supplement: Additional file 13 — Figure S1. HisKA-RR. Number of effective sequences (N eff) versus number of sequence (N) in the 60 sub-sampled HisKA-RR alignments. Dashed line indicates the diagonal. Blue line indicates a linear fit with 95 % confidence intervals in gray. Figure S2. Ovch32. Number of effective sequences (N eff) versus number of sequence (N) in the Ovch32 alignments. Dashed line indicates the diagonal. Blue line indicates a linear fit with 95 % confidence intervals in gray. Figure S3. Distribution of C β distances in HisKA-RR interaction (PDB: 3DGE). Figure S4. Distribution of C β distances in Ovch32 interactions [67] (See supplemental file for PDB accessions). Figure S5. Ovch32. Precision (PPV) versus Neff at FPR < 0.1 %. Blue lines indicate a loess fit to each method, 95 % confidence intervals are shown in gray. Figure S6. Ovch32. Power (TPR) versus Neff at FPR < 5 %. Blue lines indicate a loess fit to each method, 95 % confidence intervals are shown in gray. Figure S7. Ovch32. ϕ max versus Neff. Blue lines indicate a loess fit to each method, 95 % confidence intervals are shown in gray. Figure S8. HisKA-RR alt.. Power (TPR) vs Neff/L at FPR < 5 %. A stricter definition of positives, defined experimentally in [46–48] is used. Blue lines indicate a loess fit to each method, 95 % confidence intervals are shown in gray. Figure S9. HisKA-RR alt.. Power (TPR) vs Neff/L at FPR < 0.1 %. A stricter definition of positives, defined experimentally in [46–48] is used. Blue lines indicate a loess fit to each method, 95 % confidence intervals are shown in gray. Figure S10. HisKA-RR alt.. Precision (PPV) vs Neff/L at FPR < 0.1 %. A stricter definition of positives, defined experimentally in [46–48] is used. Blue lines indicate a loess fit to each method, 95 % confidence intervals are shown in gray. Figure S11. Ovch32. Power (TPR) at FPR < 5 % and Precision (PPV) at FPR < 0.1 % versus Neff/L. Blue lines indicate a loess fit to each method, 95 % confidence intervals are shown in gray. Figure S12 [file 12859_2015_677_MOESM13_ESM.zip › 12859_2015_677_add13/Fig_S14_FPR_0.05_Neff_per_col.pdf]

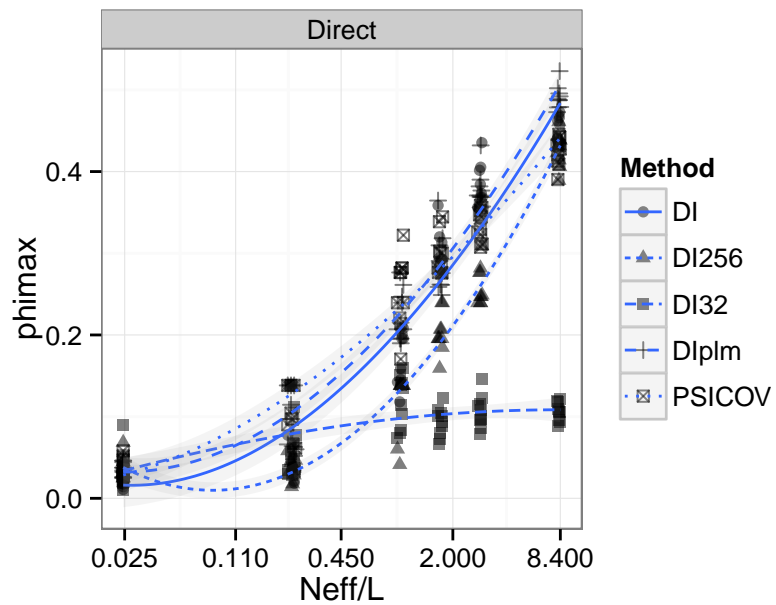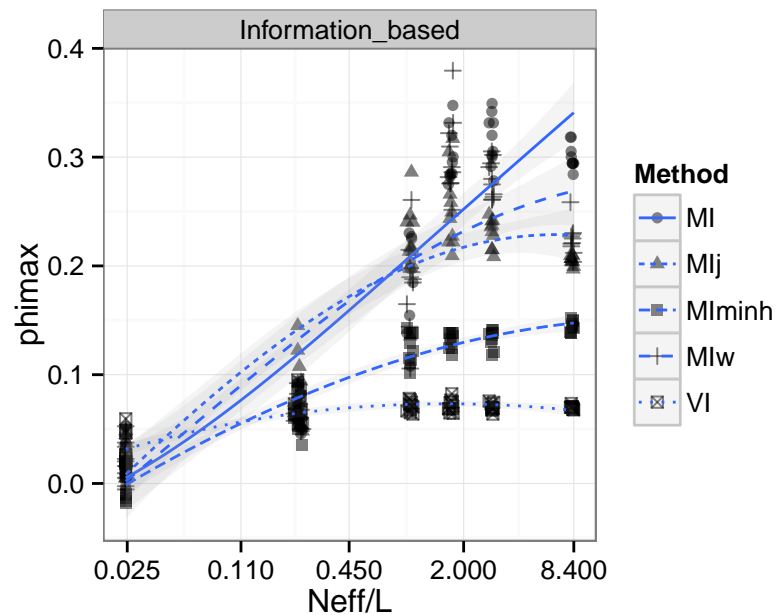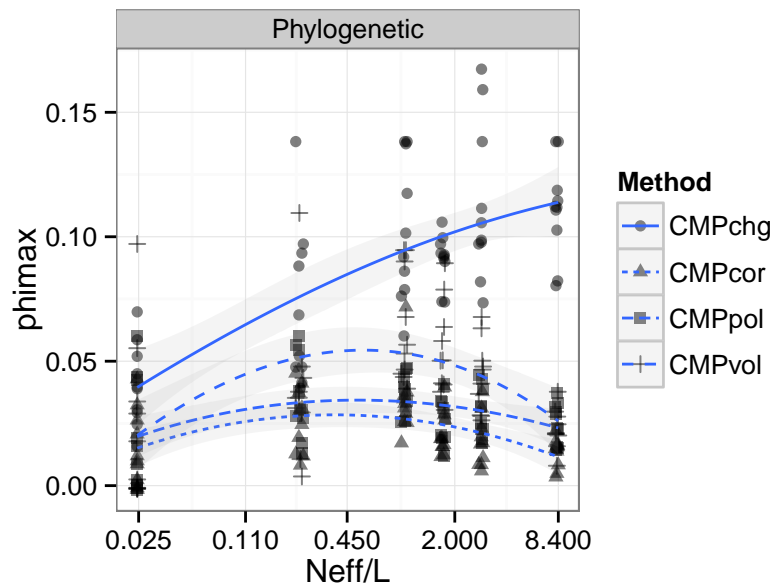

Supplement: Additional file 13 — Figure S1. HisKA-RR. Number of effective sequences (N eff) versus number of sequence (N) in the 60 sub-sampled HisKA-RR alignments. Dashed line indicates the diagonal. Blue line indicates a linear fit with 95 % confidence intervals in gray. Figure S2. Ovch32. Number of effective sequences (N eff) versus number of sequence (N) in the Ovch32 alignments. Dashed line indicates the diagonal. Blue line indicates a linear fit with 95 % confidence intervals in gray. Figure S3. Distribution of C β distances in HisKA-RR interaction (PDB: 3DGE). Figure S4. Distribution of C β distances in Ovch32 interactions [67] (See supplemental file for PDB accessions). Figure S5. Ovch32. Precision (PPV) versus Neff at FPR < 0.1 %. Blue lines indicate a loess fit to each method, 95 % confidence intervals are shown in gray. Figure S6. Ovch32. Power (TPR) versus Neff at FPR < 5 %. Blue lines indicate a loess fit to each method, 95 % confidence intervals are shown in gray. Figure S7. Ovch32. ϕ max versus Neff. Blue lines indicate a loess fit to each method, 95 % confidence intervals are shown in gray. Figure S8. HisKA-RR alt.. Power (TPR) vs Neff/L at FPR < 5 %. A stricter definition of positives, defined experimentally in [46–48] is used. Blue lines indicate a loess fit to each method, 95 % confidence intervals are shown in gray. Figure S9. HisKA-RR alt.. Power (TPR) vs Neff/L at FPR < 0.1 %. A stricter definition of positives, defined experimentally in [46–48] is used. Blue lines indicate a loess fit to each method, 95 % confidence intervals are shown in gray. Figure S10. HisKA-RR alt.. Precision (PPV) vs Neff/L at FPR < 0.1 %. A stricter definition of positives, defined experimentally in [46–48] is used. Blue lines indicate a loess fit to each method, 95 % confidence intervals are shown in gray. Figure S11. Ovch32. Power (TPR) at FPR < 5 % and Precision (PPV) at FPR < 0.1 % versus Neff/L. Blue lines indicate a loess fit to each method, 95 % confidence intervals are shown in gray. Figure S12 [file 12859_2015_677_MOESM13_ESM.zip › 12859_2015_677_add13/Fig_S15_phimax_Neff_per_col_at_NA.pdf]

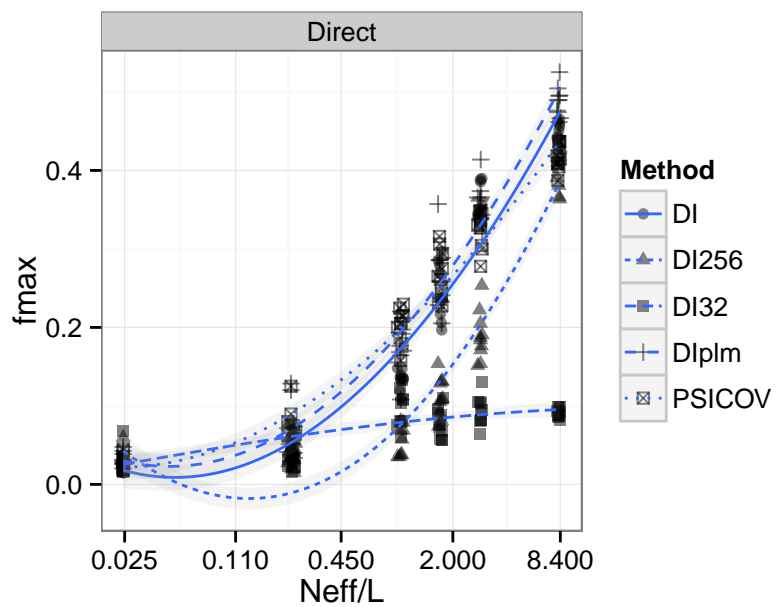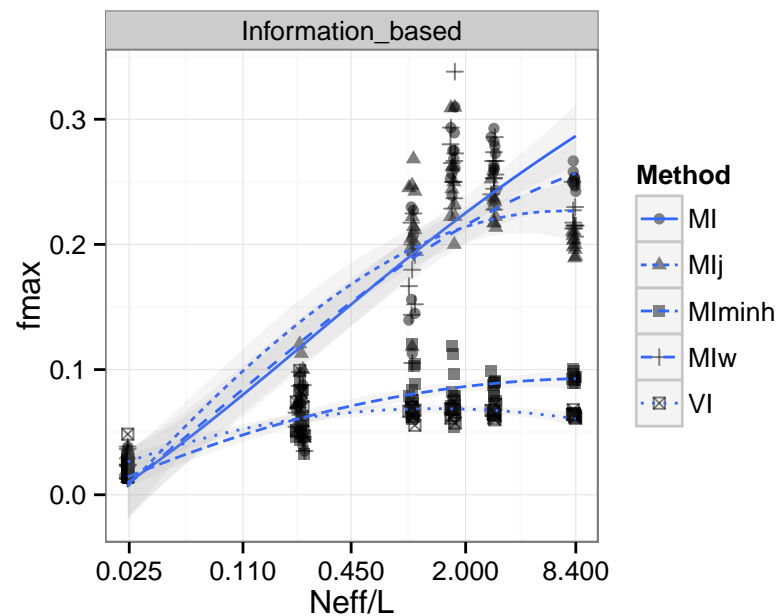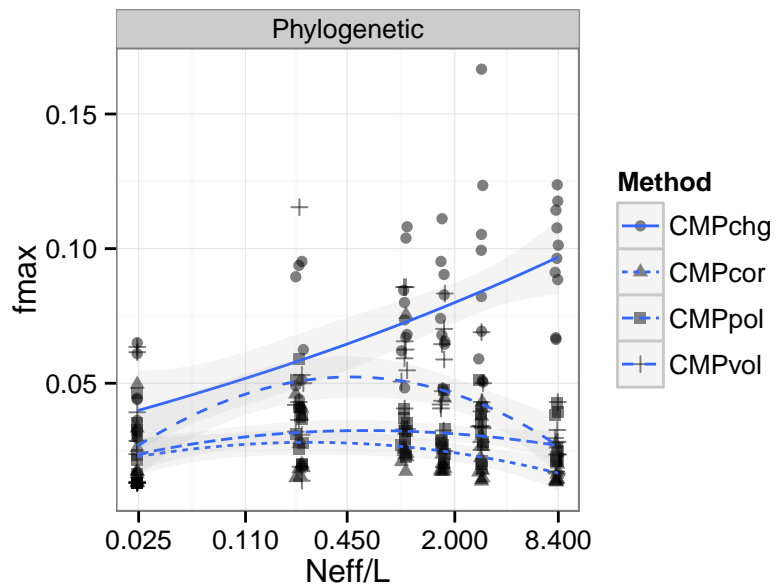

Supplement: Additional file 13 — Figure S1. HisKA-RR. Number of effective sequences (N eff) versus number of sequence (N) in the 60 sub-sampled HisKA-RR alignments. Dashed line indicates the diagonal. Blue line indicates a linear fit with 95 % confidence intervals in gray. Figure S2. Ovch32. Number of effective sequences (N eff) versus number of sequence (N) in the Ovch32 alignments. Dashed line indicates the diagonal. Blue line indicates a linear fit with 95 % confidence intervals in gray. Figure S3. Distribution of C β distances in HisKA-RR interaction (PDB: 3DGE). Figure S4. Distribution of C β distances in Ovch32 interactions [67] (See supplemental file for PDB accessions). Figure S5. Ovch32. Precision (PPV) versus Neff at FPR < 0.1 %. Blue lines indicate a loess fit to each method, 95 % confidence intervals are shown in gray. Figure S6. Ovch32. Power (TPR) versus Neff at FPR < 5 %. Blue lines indicate a loess fit to each method, 95 % confidence intervals are shown in gray. Figure S7. Ovch32. ϕ max versus Neff. Blue lines indicate a loess fit to each method, 95 % confidence intervals are shown in gray. Figure S8. HisKA-RR alt.. Power (TPR) vs Neff/L at FPR < 5 %. A stricter definition of positives, defined experimentally in [46–48] is used. Blue lines indicate a loess fit to each method, 95 % confidence intervals are shown in gray. Figure S9. HisKA-RR alt.. Power (TPR) vs Neff/L at FPR < 0.1 %. A stricter definition of positives, defined experimentally in [46–48] is used. Blue lines indicate a loess fit to each method, 95 % confidence intervals are shown in gray. Figure S10. HisKA-RR alt.. Precision (PPV) vs Neff/L at FPR < 0.1 %. A stricter definition of positives, defined experimentally in [46–48] is used. Blue lines indicate a loess fit to each method, 95 % confidence intervals are shown in gray. Figure S11. Ovch32. Power (TPR) at FPR < 5 % and Precision (PPV) at FPR < 0.1 % versus Neff/L. Blue lines indicate a loess fit to each method, 95 % confidence intervals are shown in gray. Figure S12 [file 12859_2015_677_MOESM13_ESM.zip › 12859_2015_677_add13/Fig_S16_fmax_Neff_per_col_at_NA.pdf]

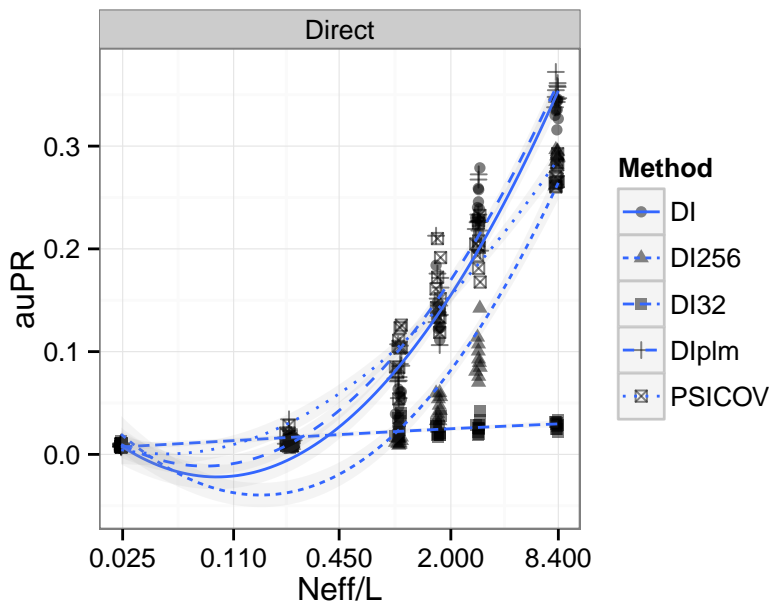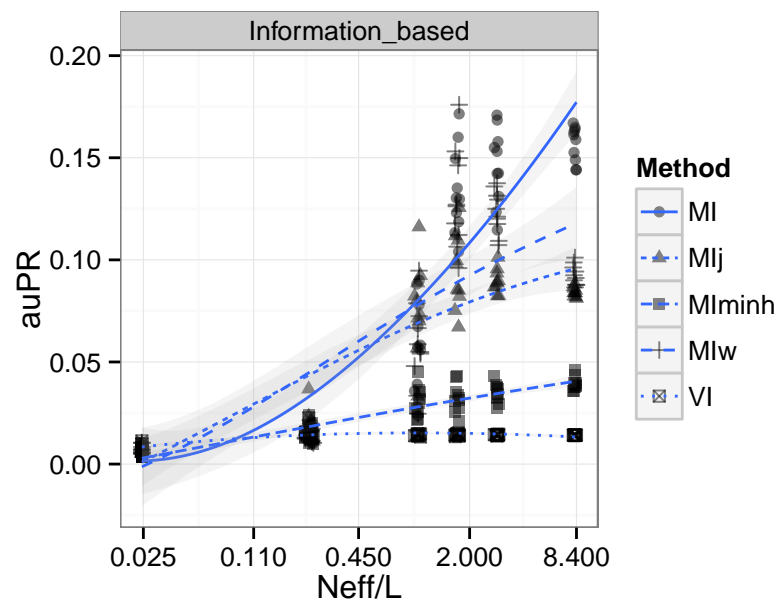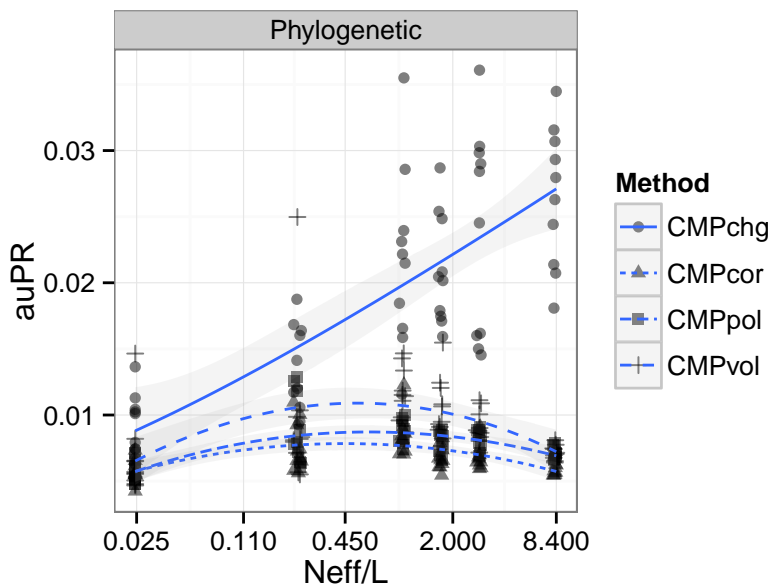

Supplement: Additional file 13 — Figure S1. HisKA-RR. Number of effective sequences (N eff) versus number of sequence (N) in the 60 sub-sampled HisKA-RR alignments. Dashed line indicates the diagonal. Blue line indicates a linear fit with 95 % confidence intervals in gray. Figure S2. Ovch32. Number of effective sequences (N eff) versus number of sequence (N) in the Ovch32 alignments. Dashed line indicates the diagonal. Blue line indicates a linear fit with 95 % confidence intervals in gray. Figure S3. Distribution of C β distances in HisKA-RR interaction (PDB: 3DGE). Figure S4. Distribution of C β distances in Ovch32 interactions [67] (See supplemental file for PDB accessions). Figure S5. Ovch32. Precision (PPV) versus Neff at FPR < 0.1 %. Blue lines indicate a loess fit to each method, 95 % confidence intervals are shown in gray. Figure S6. Ovch32. Power (TPR) versus Neff at FPR < 5 %. Blue lines indicate a loess fit to each method, 95 % confidence intervals are shown in gray. Figure S7. Ovch32. ϕ max versus Neff. Blue lines indicate a loess fit to each method, 95 % confidence intervals are shown in gray. Figure S8. HisKA-RR alt.. Power (TPR) vs Neff/L at FPR < 5 %. A stricter definition of positives, defined experimentally in [46–48] is used. Blue lines indicate a loess fit to each method, 95 % confidence intervals are shown in gray. Figure S9. HisKA-RR alt.. Power (TPR) vs Neff/L at FPR < 0.1 %. A stricter definition of positives, defined experimentally in [46–48] is used. Blue lines indicate a loess fit to each method, 95 % confidence intervals are shown in gray. Figure S10. HisKA-RR alt.. Precision (PPV) vs Neff/L at FPR < 0.1 %. A stricter definition of positives, defined experimentally in [46–48] is used. Blue lines indicate a loess fit to each method, 95 % confidence intervals are shown in gray. Figure S11. Ovch32. Power (TPR) at FPR < 5 % and Precision (PPV) at FPR < 0.1 % versus Neff/L. Blue lines indicate a loess fit to each method, 95 % confidence intervals are shown in gray. Figure S12 [file 12859_2015_677_MOESM13_ESM.zip › 12859_2015_677_add13/Fig_S17_auPR_Neff_per_col_at_NA.pdf]

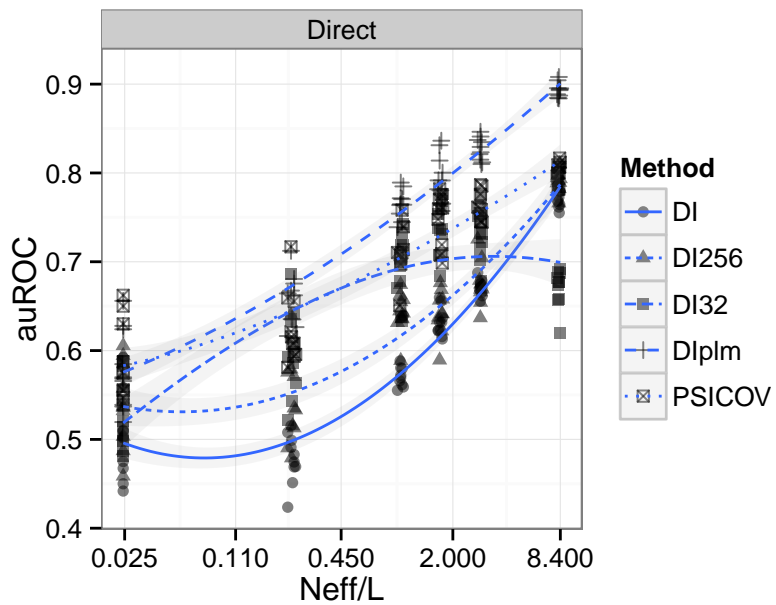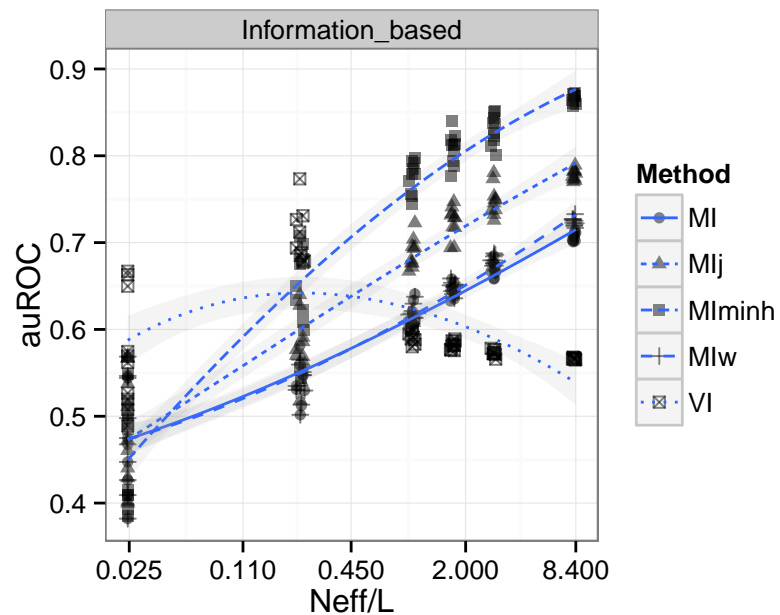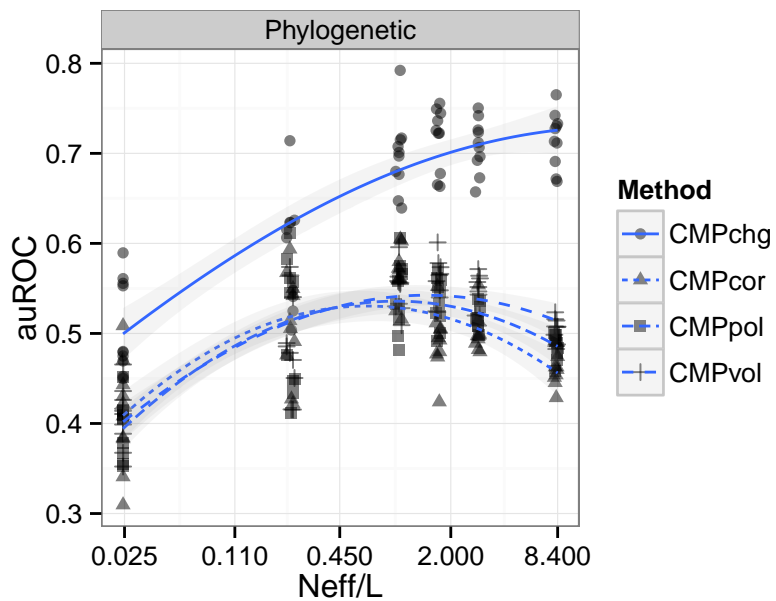

Supplement: Additional file 13 — Figure S1. HisKA-RR. Number of effective sequences (N eff) versus number of sequence (N) in the 60 sub-sampled HisKA-RR alignments. Dashed line indicates the diagonal. Blue line indicates a linear fit with 95 % confidence intervals in gray. Figure S2. Ovch32. Number of effective sequences (N eff) versus number of sequence (N) in the Ovch32 alignments. Dashed line indicates the diagonal. Blue line indicates a linear fit with 95 % confidence intervals in gray. Figure S3. Distribution of C β distances in HisKA-RR interaction (PDB: 3DGE). Figure S4. Distribution of C β distances in Ovch32 interactions [67] (See supplemental file for PDB accessions). Figure S5. Ovch32. Precision (PPV) versus Neff at FPR < 0.1 %. Blue lines indicate a loess fit to each method, 95 % confidence intervals are shown in gray. Figure S6. Ovch32. Power (TPR) versus Neff at FPR < 5 %. Blue lines indicate a loess fit to each method, 95 % confidence intervals are shown in gray. Figure S7. Ovch32. ϕ max versus Neff. Blue lines indicate a loess fit to each method, 95 % confidence intervals are shown in gray. Figure S8. HisKA-RR alt.. Power (TPR) vs Neff/L at FPR < 5 %. A stricter definition of positives, defined experimentally in [46–48] is used. Blue lines indicate a loess fit to each method, 95 % confidence intervals are shown in gray. Figure S9. HisKA-RR alt.. Power (TPR) vs Neff/L at FPR < 0.1 %. A stricter definition of positives, defined experimentally in [46–48] is used. Blue lines indicate a loess fit to each method, 95 % confidence intervals are shown in gray. Figure S10. HisKA-RR alt.. Precision (PPV) vs Neff/L at FPR < 0.1 %. A stricter definition of positives, defined experimentally in [46–48] is used. Blue lines indicate a loess fit to each method, 95 % confidence intervals are shown in gray. Figure S11. Ovch32. Power (TPR) at FPR < 5 % and Precision (PPV) at FPR < 0.1 % versus Neff/L. Blue lines indicate a loess fit to each method, 95 % confidence intervals are shown in gray. Figure S12 [file 12859_2015_677_MOESM13_ESM.zip › 12859_2015_677_add13/Fig_S18_auROC_Neff_per_col_at_NA.pdf]

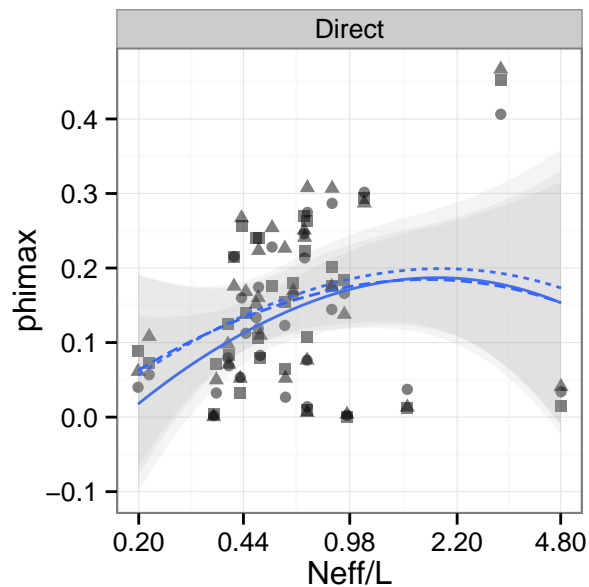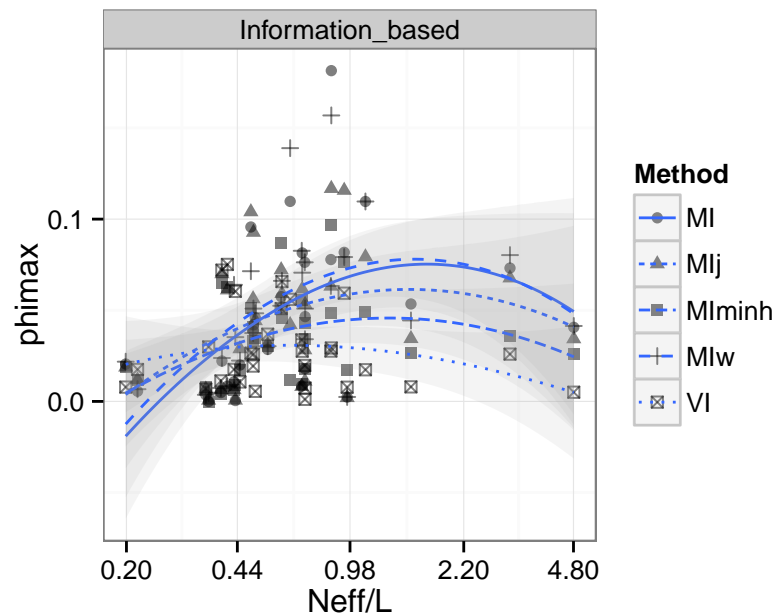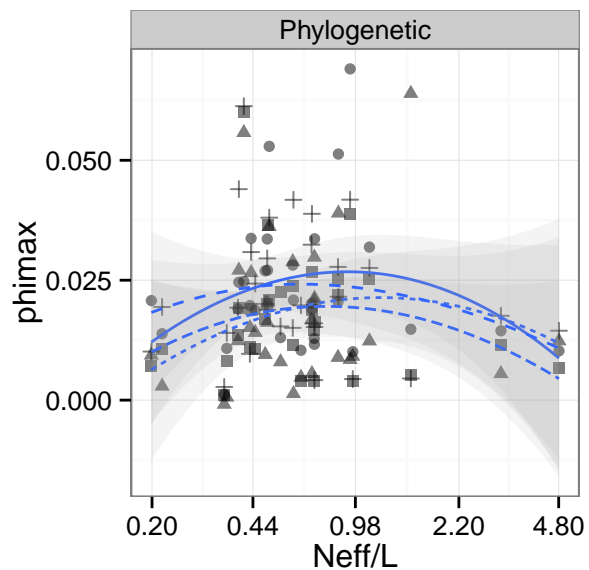

Supplement: Additional file 13 — Figure S1. HisKA-RR. Number of effective sequences (N eff) versus number of sequence (N) in the 60 sub-sampled HisKA-RR alignments. Dashed line indicates the diagonal. Blue line indicates a linear fit with 95 % confidence intervals in gray. Figure S2. Ovch32. Number of effective sequences (N eff) versus number of sequence (N) in the Ovch32 alignments. Dashed line indicates the diagonal. Blue line indicates a linear fit with 95 % confidence intervals in gray. Figure S3. Distribution of C β distances in HisKA-RR interaction (PDB: 3DGE). Figure S4. Distribution of C β distances in Ovch32 interactions [67] (See supplemental file for PDB accessions). Figure S5. Ovch32. Precision (PPV) versus Neff at FPR < 0.1 %. Blue lines indicate a loess fit to each method, 95 % confidence intervals are shown in gray. Figure S6. Ovch32. Power (TPR) versus Neff at FPR < 5 %. Blue lines indicate a loess fit to each method, 95 % confidence intervals are shown in gray. Figure S7. Ovch32. ϕ max versus Neff. Blue lines indicate a loess fit to each method, 95 % confidence intervals are shown in gray. Figure S8. HisKA-RR alt.. Power (TPR) vs Neff/L at FPR < 5 %. A stricter definition of positives, defined experimentally in [46–48] is used. Blue lines indicate a loess fit to each method, 95 % confidence intervals are shown in gray. Figure S9. HisKA-RR alt.. Power (TPR) vs Neff/L at FPR < 0.1 %. A stricter definition of positives, defined experimentally in [46–48] is used. Blue lines indicate a loess fit to each method, 95 % confidence intervals are shown in gray. Figure S10. HisKA-RR alt.. Precision (PPV) vs Neff/L at FPR < 0.1 %. A stricter definition of positives, defined experimentally in [46–48] is used. Blue lines indicate a loess fit to each method, 95 % confidence intervals are shown in gray. Figure S11. Ovch32. Power (TPR) at FPR < 5 % and Precision (PPV) at FPR < 0.1 % versus Neff/L. Blue lines indicate a loess fit to each method, 95 % confidence intervals are shown in gray. Figure S12 [file 12859_2015_677_MOESM13_ESM.zip › 12859_2015_677_add13/Fig_S19_phimax_Neff_per_col_at_NA.pdf]

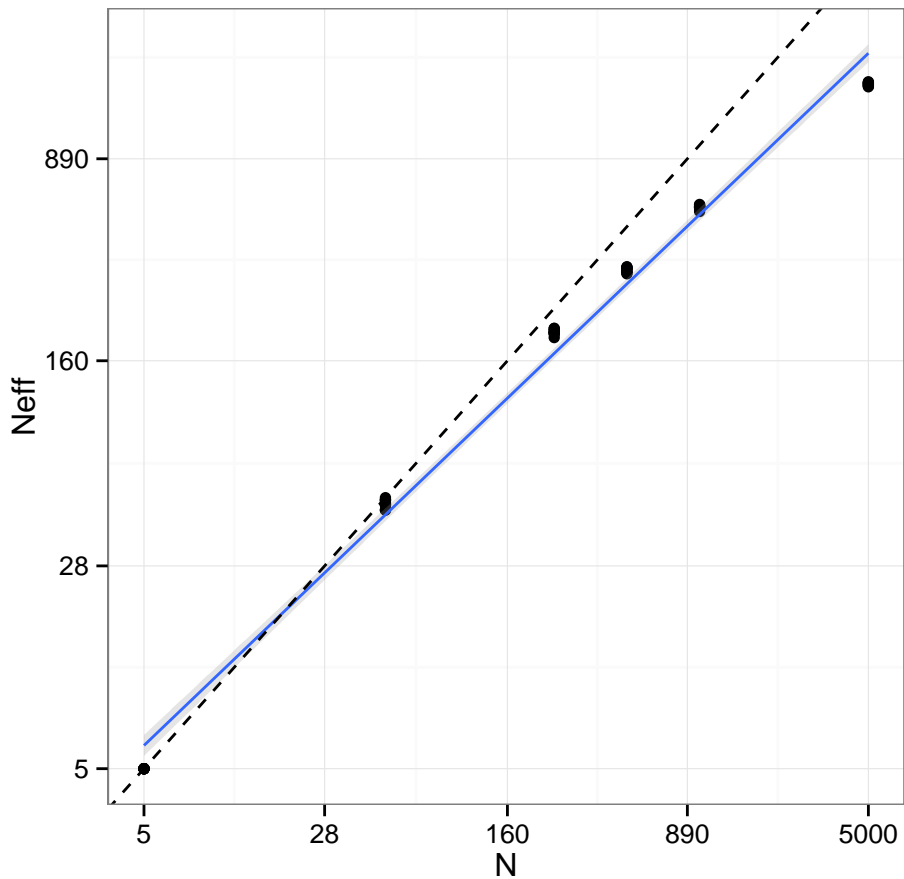

Supplement: Additional file 13 — Figure S1. HisKA-RR. Number of effective sequences (N eff) versus number of sequence (N) in the 60 sub-sampled HisKA-RR alignments. Dashed line indicates the diagonal. Blue line indicates a linear fit with 95 % confidence intervals in gray. Figure S2. Ovch32. Number of effective sequences (N eff) versus number of sequence (N) in the Ovch32 alignments. Dashed line indicates the diagonal. Blue line indicates a linear fit with 95 % confidence intervals in gray. Figure S3. Distribution of C β distances in HisKA-RR interaction (PDB: 3DGE). Figure S4. Distribution of C β distances in Ovch32 interactions [67] (See supplemental file for PDB accessions). Figure S5. Ovch32. Precision (PPV) versus Neff at FPR < 0.1 %. Blue lines indicate a loess fit to each method, 95 % confidence intervals are shown in gray. Figure S6. Ovch32. Power (TPR) versus Neff at FPR < 5 %. Blue lines indicate a loess fit to each method, 95 % confidence intervals are shown in gray. Figure S7. Ovch32. ϕ max versus Neff. Blue lines indicate a loess fit to each method, 95 % confidence intervals are shown in gray. Figure S8. HisKA-RR alt.. Power (TPR) vs Neff/L at FPR < 5 %. A stricter definition of positives, defined experimentally in [46–48] is used. Blue lines indicate a loess fit to each method, 95 % confidence intervals are shown in gray. Figure S9. HisKA-RR alt.. Power (TPR) vs Neff/L at FPR < 0.1 %. A stricter definition of positives, defined experimentally in [46–48] is used. Blue lines indicate a loess fit to each method, 95 % confidence intervals are shown in gray. Figure S10. HisKA-RR alt.. Precision (PPV) vs Neff/L at FPR < 0.1 %. A stricter definition of positives, defined experimentally in [46–48] is used. Blue lines indicate a loess fit to each method, 95 % confidence intervals are shown in gray. Figure S11. Ovch32. Power (TPR) at FPR < 5 % and Precision (PPV) at FPR < 0.1 % versus Neff/L. Blue lines indicate a loess fit to each method, 95 % confidence intervals are shown in gray. Figure S12 [file 12859_2015_677_MOESM13_ESM.zip › 12859_2015_677_add13/Fig_S1_nneff.pdf]

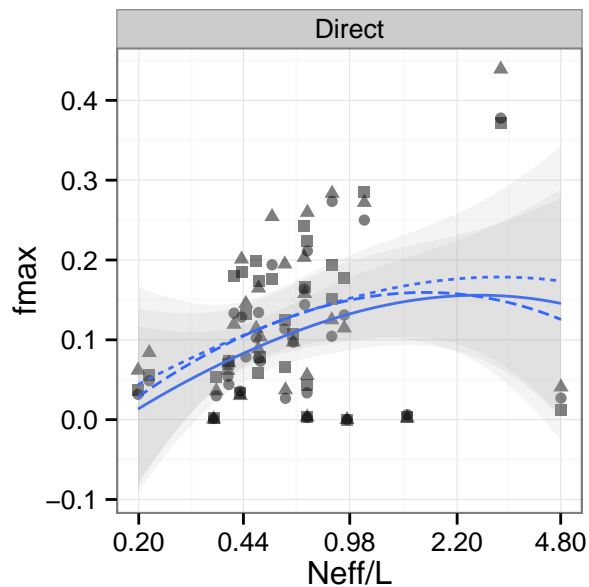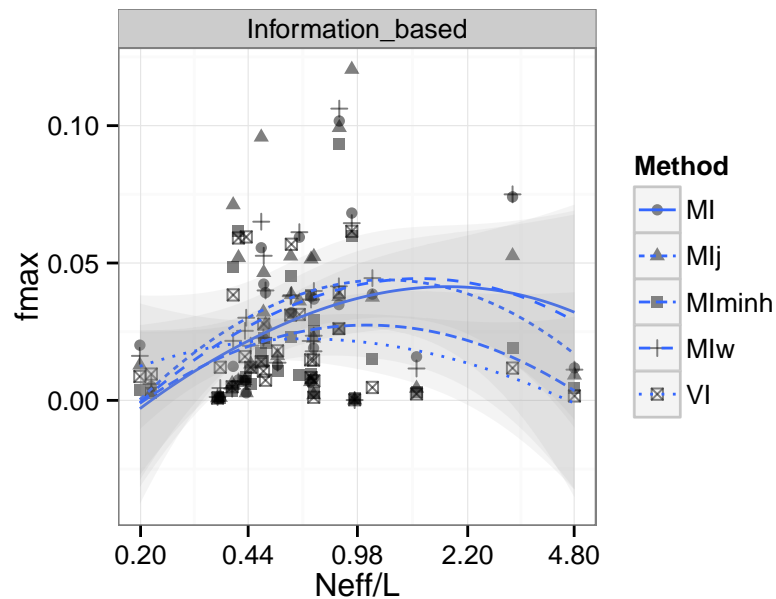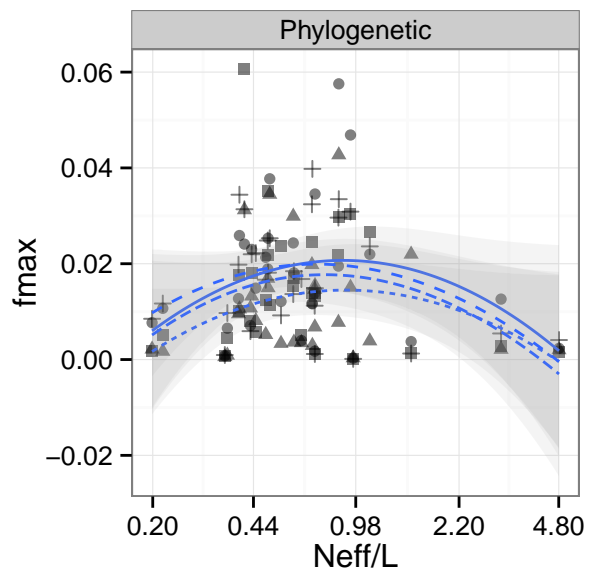

Supplement: Additional file 13 — Figure S1. HisKA-RR. Number of effective sequences (N eff) versus number of sequence (N) in the 60 sub-sampled HisKA-RR alignments. Dashed line indicates the diagonal. Blue line indicates a linear fit with 95 % confidence intervals in gray. Figure S2. Ovch32. Number of effective sequences (N eff) versus number of sequence (N) in the Ovch32 alignments. Dashed line indicates the diagonal. Blue line indicates a linear fit with 95 % confidence intervals in gray. Figure S3. Distribution of C β distances in HisKA-RR interaction (PDB: 3DGE). Figure S4. Distribution of C β distances in Ovch32 interactions [67] (See supplemental file for PDB accessions). Figure S5. Ovch32. Precision (PPV) versus Neff at FPR < 0.1 %. Blue lines indicate a loess fit to each method, 95 % confidence intervals are shown in gray. Figure S6. Ovch32. Power (TPR) versus Neff at FPR < 5 %. Blue lines indicate a loess fit to each method, 95 % confidence intervals are shown in gray. Figure S7. Ovch32. ϕ max versus Neff. Blue lines indicate a loess fit to each method, 95 % confidence intervals are shown in gray. Figure S8. HisKA-RR alt.. Power (TPR) vs Neff/L at FPR < 5 %. A stricter definition of positives, defined experimentally in [46–48] is used. Blue lines indicate a loess fit to each method, 95 % confidence intervals are shown in gray. Figure S9. HisKA-RR alt.. Power (TPR) vs Neff/L at FPR < 0.1 %. A stricter definition of positives, defined experimentally in [46–48] is used. Blue lines indicate a loess fit to each method, 95 % confidence intervals are shown in gray. Figure S10. HisKA-RR alt.. Precision (PPV) vs Neff/L at FPR < 0.1 %. A stricter definition of positives, defined experimentally in [46–48] is used. Blue lines indicate a loess fit to each method, 95 % confidence intervals are shown in gray. Figure S11. Ovch32. Power (TPR) at FPR < 5 % and Precision (PPV) at FPR < 0.1 % versus Neff/L. Blue lines indicate a loess fit to each method, 95 % confidence intervals are shown in gray. Figure S12 [file 12859_2015_677_MOESM13_ESM.zip › 12859_2015_677_add13/Fig_S20_fmax_Neff_per_col_at_NA.pdf]

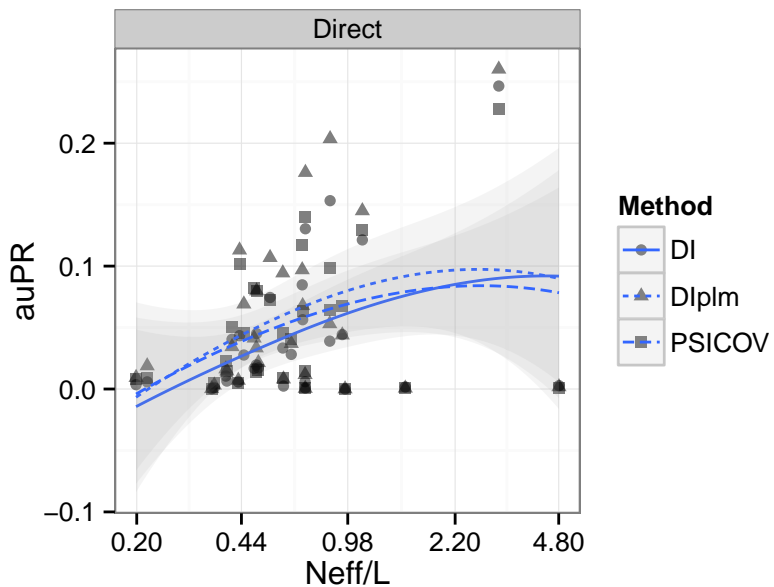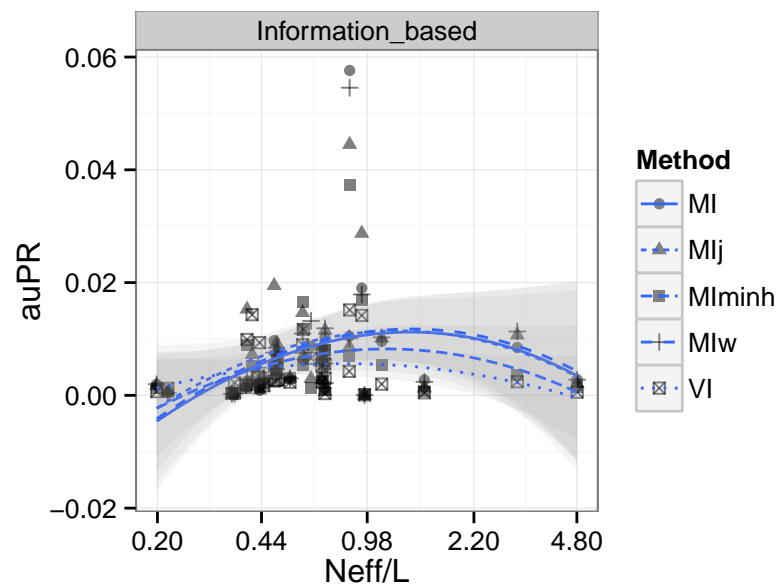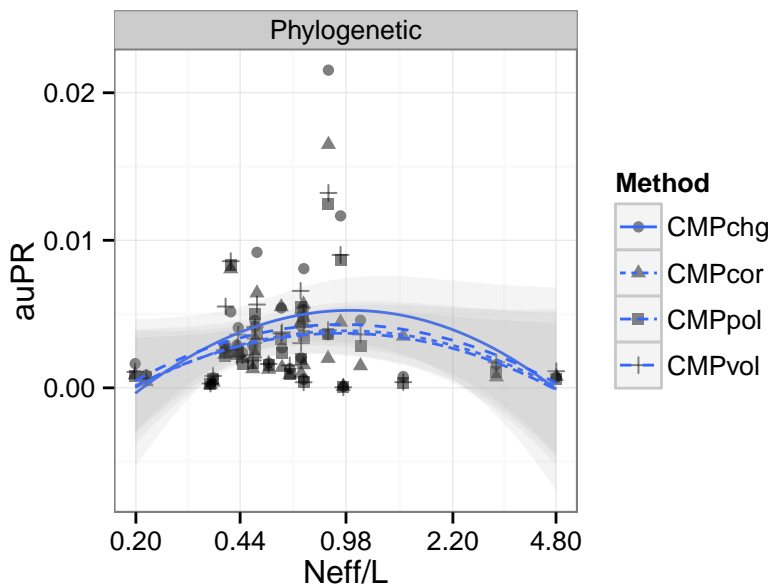

Supplement: Additional file 13 — Figure S1. HisKA-RR. Number of effective sequences (N eff) versus number of sequence (N) in the 60 sub-sampled HisKA-RR alignments. Dashed line indicates the diagonal. Blue line indicates a linear fit with 95 % confidence intervals in gray. Figure S2. Ovch32. Number of effective sequences (N eff) versus number of sequence (N) in the Ovch32 alignments. Dashed line indicates the diagonal. Blue line indicates a linear fit with 95 % confidence intervals in gray. Figure S3. Distribution of C β distances in HisKA-RR interaction (PDB: 3DGE). Figure S4. Distribution of C β distances in Ovch32 interactions [67] (See supplemental file for PDB accessions). Figure S5. Ovch32. Precision (PPV) versus Neff at FPR < 0.1 %. Blue lines indicate a loess fit to each method, 95 % confidence intervals are shown in gray. Figure S6. Ovch32. Power (TPR) versus Neff at FPR < 5 %. Blue lines indicate a loess fit to each method, 95 % confidence intervals are shown in gray. Figure S7. Ovch32. ϕ max versus Neff. Blue lines indicate a loess fit to each method, 95 % confidence intervals are shown in gray. Figure S8. HisKA-RR alt.. Power (TPR) vs Neff/L at FPR < 5 %. A stricter definition of positives, defined experimentally in [46–48] is used. Blue lines indicate a loess fit to each method, 95 % confidence intervals are shown in gray. Figure S9. HisKA-RR alt.. Power (TPR) vs Neff/L at FPR < 0.1 %. A stricter definition of positives, defined experimentally in [46–48] is used. Blue lines indicate a loess fit to each method, 95 % confidence intervals are shown in gray. Figure S10. HisKA-RR alt.. Precision (PPV) vs Neff/L at FPR < 0.1 %. A stricter definition of positives, defined experimentally in [46–48] is used. Blue lines indicate a loess fit to each method, 95 % confidence intervals are shown in gray. Figure S11. Ovch32. Power (TPR) at FPR < 5 % and Precision (PPV) at FPR < 0.1 % versus Neff/L. Blue lines indicate a loess fit to each method, 95 % confidence intervals are shown in gray. Figure S12 [file 12859_2015_677_MOESM13_ESM.zip › 12859_2015_677_add13/Fig_S21_auPR_Neff_per_col_at_NA.pdf]

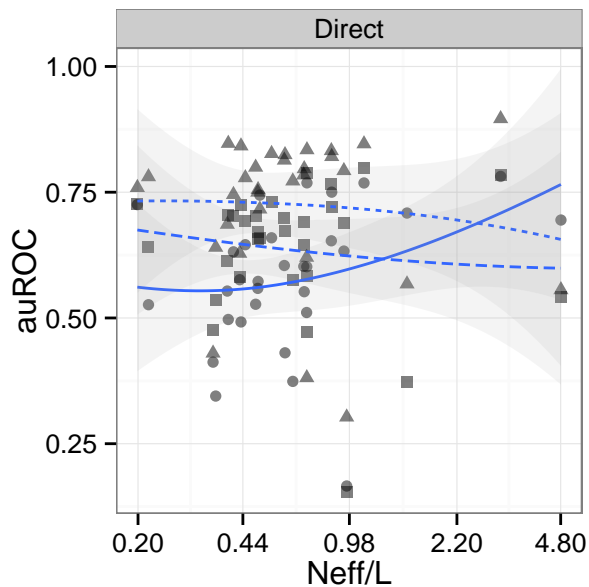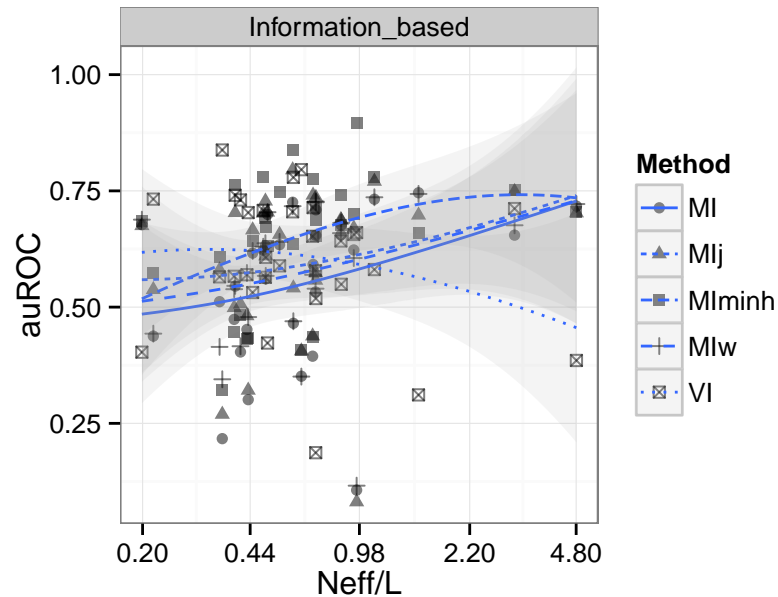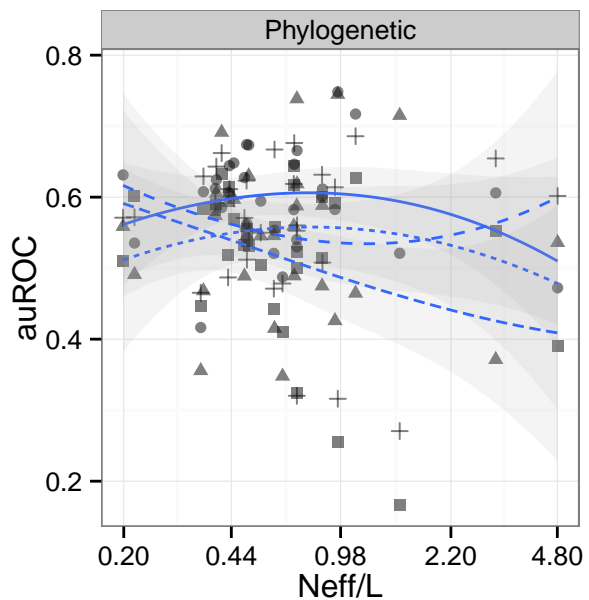

Supplement: Additional file 13 — Figure S1. HisKA-RR. Number of effective sequences (N eff) versus number of sequence (N) in the 60 sub-sampled HisKA-RR alignments. Dashed line indicates the diagonal. Blue line indicates a linear fit with 95 % confidence intervals in gray. Figure S2. Ovch32. Number of effective sequences (N eff) versus number of sequence (N) in the Ovch32 alignments. Dashed line indicates the diagonal. Blue line indicates a linear fit with 95 % confidence intervals in gray. Figure S3. Distribution of C β distances in HisKA-RR interaction (PDB: 3DGE). Figure S4. Distribution of C β distances in Ovch32 interactions [67] (See supplemental file for PDB accessions). Figure S5. Ovch32. Precision (PPV) versus Neff at FPR < 0.1 %. Blue lines indicate a loess fit to each method, 95 % confidence intervals are shown in gray. Figure S6. Ovch32. Power (TPR) versus Neff at FPR < 5 %. Blue lines indicate a loess fit to each method, 95 % confidence intervals are shown in gray. Figure S7. Ovch32. ϕ max versus Neff. Blue lines indicate a loess fit to each method, 95 % confidence intervals are shown in gray. Figure S8. HisKA-RR alt.. Power (TPR) vs Neff/L at FPR < 5 %. A stricter definition of positives, defined experimentally in [46–48] is used. Blue lines indicate a loess fit to each method, 95 % confidence intervals are shown in gray. Figure S9. HisKA-RR alt.. Power (TPR) vs Neff/L at FPR < 0.1 %. A stricter definition of positives, defined experimentally in [46–48] is used. Blue lines indicate a loess fit to each method, 95 % confidence intervals are shown in gray. Figure S10. HisKA-RR alt.. Precision (PPV) vs Neff/L at FPR < 0.1 %. A stricter definition of positives, defined experimentally in [46–48] is used. Blue lines indicate a loess fit to each method, 95 % confidence intervals are shown in gray. Figure S11. Ovch32. Power (TPR) at FPR < 5 % and Precision (PPV) at FPR < 0.1 % versus Neff/L. Blue lines indicate a loess fit to each method, 95 % confidence intervals are shown in gray. Figure S12 [file 12859_2015_677_MOESM13_ESM.zip › 12859_2015_677_add13/Fig_S22_auROC_Neff_per_col_at_NA.pdf]

Left and Right entropy ranges [nats]

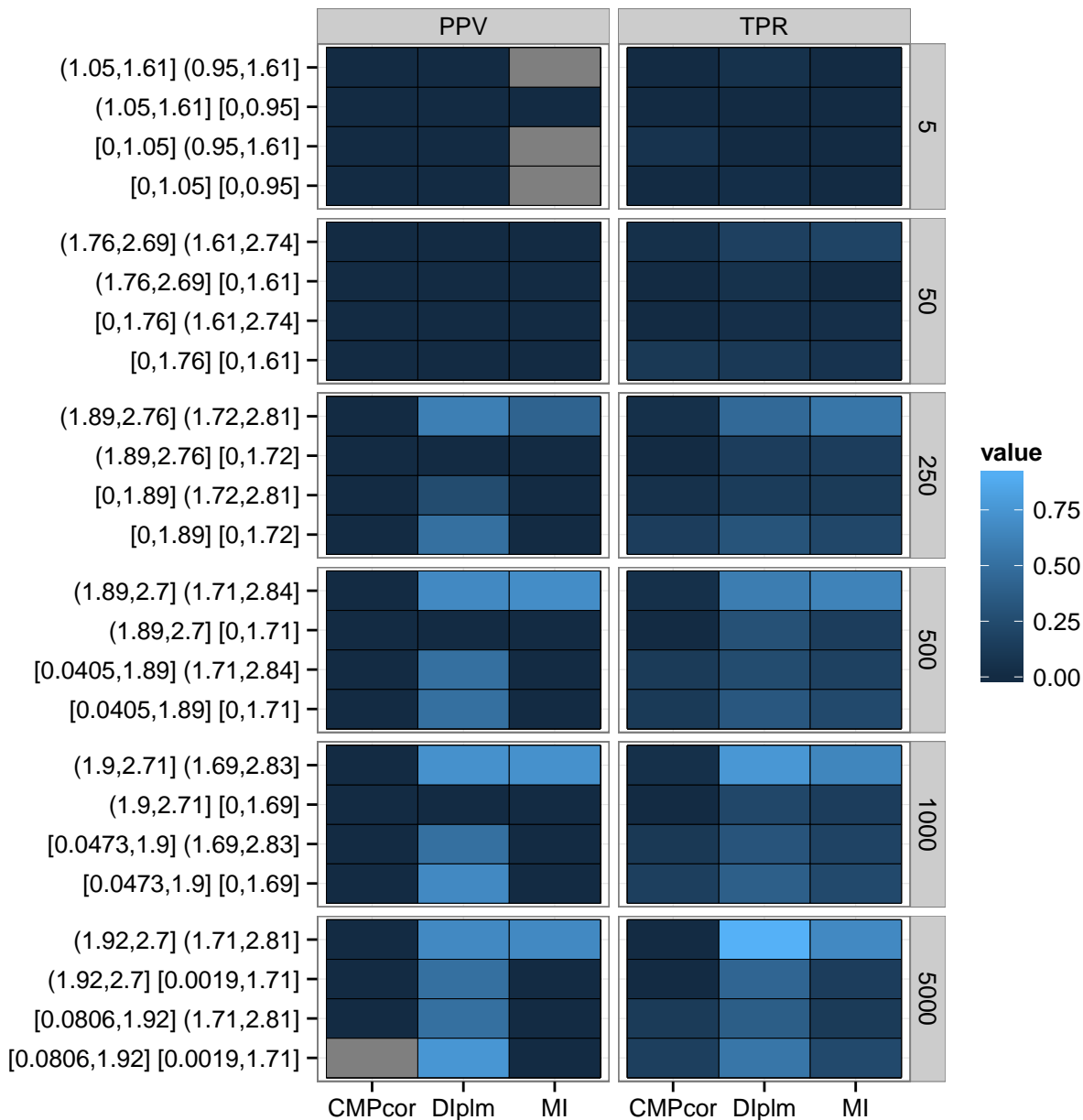

Method

Supplement: Additional file 13 — Figure S1. HisKA-RR. Number of effective sequences (N eff) versus number of sequence (N) in the 60 sub-sampled HisKA-RR alignments. Dashed line indicates the diagonal. Blue line indicates a linear fit with 95 % confidence intervals in gray. Figure S2. Ovch32. Number of effective sequences (N eff) versus number of sequence (N) in the Ovch32 alignments. Dashed line indicates the diagonal. Blue line indicates a linear fit with 95 % confidence intervals in gray. Figure S3. Distribution of C β distances in HisKA-RR interaction (PDB: 3DGE). Figure S4. Distribution of C β distances in Ovch32 interactions [67] (See supplemental file for PDB accessions). Figure S5. Ovch32. Precision (PPV) versus Neff at FPR < 0.1 %. Blue lines indicate a loess fit to each method, 95 % confidence intervals are shown in gray. Figure S6. Ovch32. Power (TPR) versus Neff at FPR < 5 %. Blue lines indicate a loess fit to each method, 95 % confidence intervals are shown in gray. Figure S7. Ovch32. ϕ max versus Neff. Blue lines indicate a loess fit to each method, 95 % confidence intervals are shown in gray. Figure S8. HisKA-RR alt.. Power (TPR) vs Neff/L at FPR < 5 %. A stricter definition of positives, defined experimentally in [46–48] is used. Blue lines indicate a loess fit to each method, 95 % confidence intervals are shown in gray. Figure S9. HisKA-RR alt.. Power (TPR) vs Neff/L at FPR < 0.1 %. A stricter definition of positives, defined experimentally in [46–48] is used. Blue lines indicate a loess fit to each method, 95 % confidence intervals are shown in gray. Figure S10. HisKA-RR alt.. Precision (PPV) vs Neff/L at FPR < 0.1 %. A stricter definition of positives, defined experimentally in [46–48] is used. Blue lines indicate a loess fit to each method, 95 % confidence intervals are shown in gray. Figure S11. Ovch32. Power (TPR) at FPR < 5 % and Precision (PPV) at FPR < 0.1 % versus Neff/L. Blue lines indicate a loess fit to each method, 95 % confidence intervals are shown in gray. Figure S12 [file 12859_2015_677_MOESM13_ESM.zip › 12859_2015_677_add13/Fig_S23_select_perf_by_ratecats.pdf]

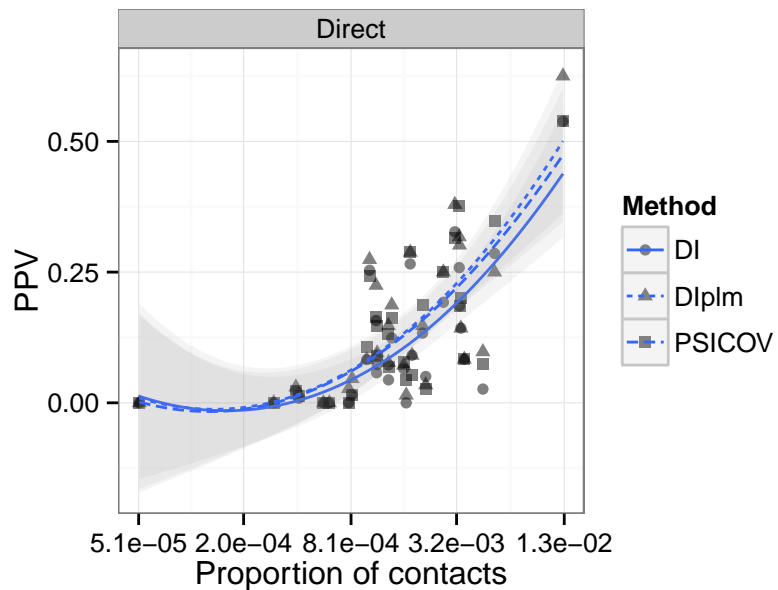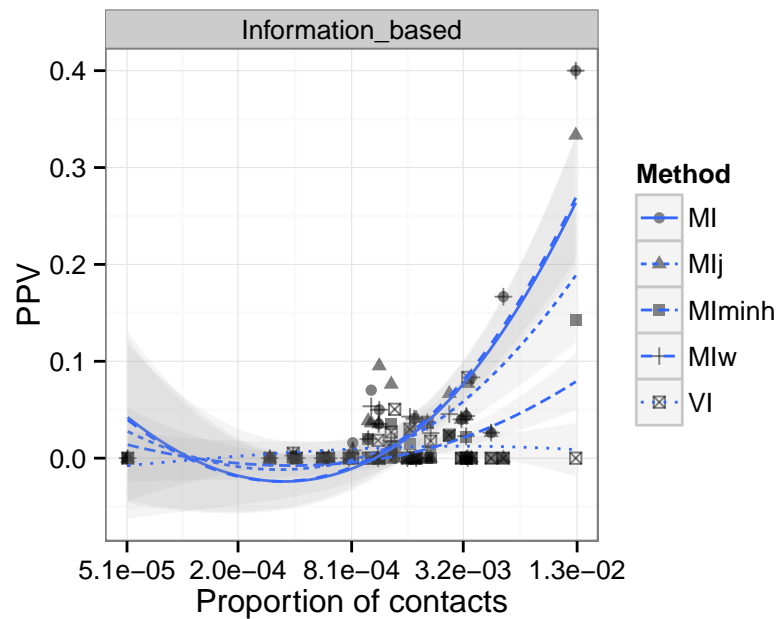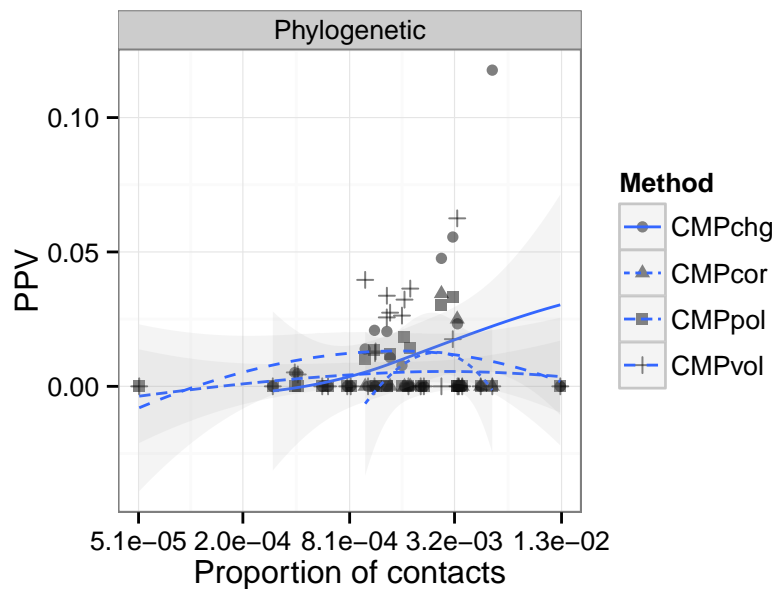

Supplement: Additional file 13 — Figure S1. HisKA-RR. Number of effective sequences (N eff) versus number of sequence (N) in the 60 sub-sampled HisKA-RR alignments. Dashed line indicates the diagonal. Blue line indicates a linear fit with 95 % confidence intervals in gray. Figure S2. Ovch32. Number of effective sequences (N eff) versus number of sequence (N) in the Ovch32 alignments. Dashed line indicates the diagonal. Blue line indicates a linear fit with 95 % confidence intervals in gray. Figure S3. Distribution of C β distances in HisKA-RR interaction (PDB: 3DGE). Figure S4. Distribution of C β distances in Ovch32 interactions [67] (See supplemental file for PDB accessions). Figure S5. Ovch32. Precision (PPV) versus Neff at FPR < 0.1 %. Blue lines indicate a loess fit to each method, 95 % confidence intervals are shown in gray. Figure S6. Ovch32. Power (TPR) versus Neff at FPR < 5 %. Blue lines indicate a loess fit to each method, 95 % confidence intervals are shown in gray. Figure S7. Ovch32. ϕ max versus Neff. Blue lines indicate a loess fit to each method, 95 % confidence intervals are shown in gray. Figure S8. HisKA-RR alt.. Power (TPR) vs Neff/L at FPR < 5 %. A stricter definition of positives, defined experimentally in [46–48] is used. Blue lines indicate a loess fit to each method, 95 % confidence intervals are shown in gray. Figure S9. HisKA-RR alt.. Power (TPR) vs Neff/L at FPR < 0.1 %. A stricter definition of positives, defined experimentally in [46–48] is used. Blue lines indicate a loess fit to each method, 95 % confidence intervals are shown in gray. Figure S10. HisKA-RR alt.. Precision (PPV) vs Neff/L at FPR < 0.1 %. A stricter definition of positives, defined experimentally in [46–48] is used. Blue lines indicate a loess fit to each method, 95 % confidence intervals are shown in gray. Figure S11. Ovch32. Power (TPR) at FPR < 5 % and Precision (PPV) at FPR < 0.1 % versus Neff/L. Blue lines indicate a loess fit to each method, 95 % confidence intervals are shown in gray. Figure S12 [file 12859_2015_677_MOESM13_ESM.zip › 12859_2015_677_add13/Fig_S24_PPV_Prop_Contacts_at_0.001.pdf]

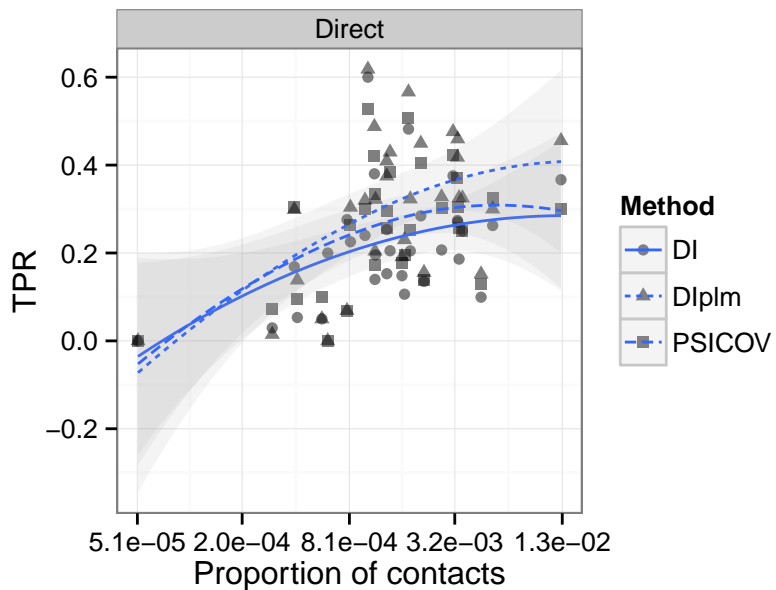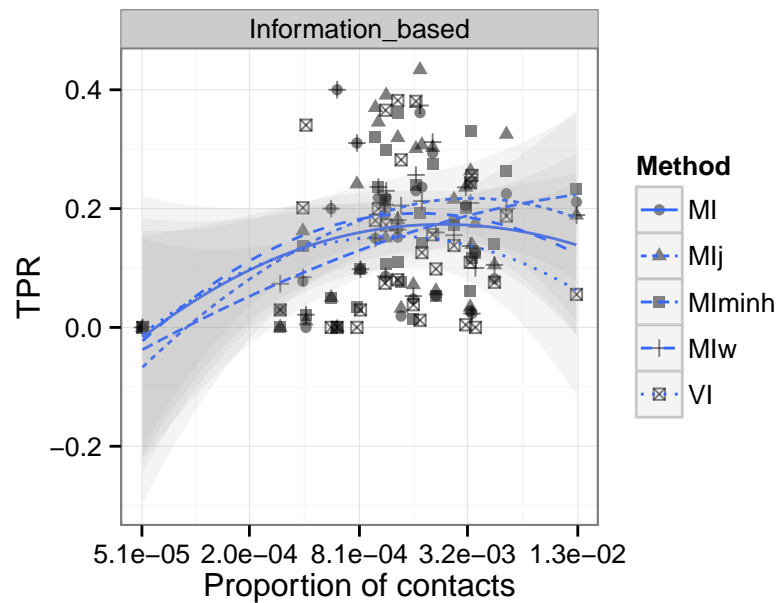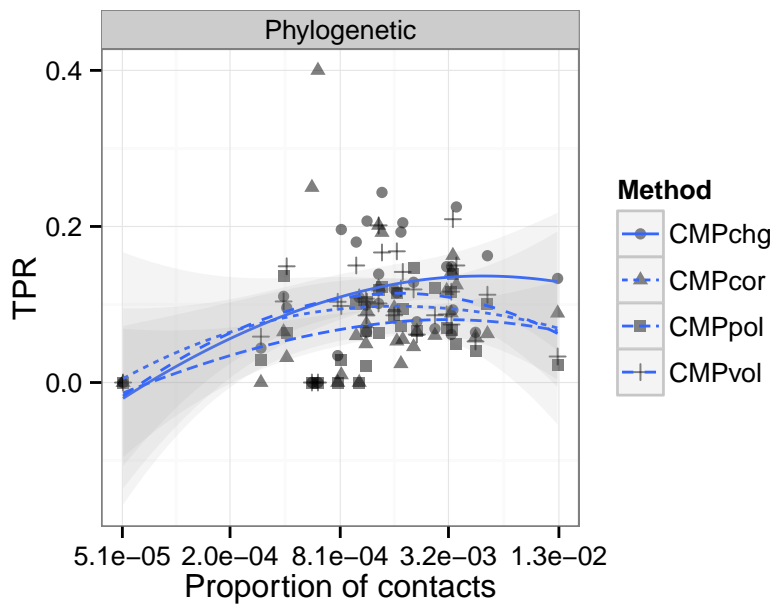

Supplement: Additional file 13 — Figure S1. HisKA-RR. Number of effective sequences (N eff) versus number of sequence (N) in the 60 sub-sampled HisKA-RR alignments. Dashed line indicates the diagonal. Blue line indicates a linear fit with 95 % confidence intervals in gray. Figure S2. Ovch32. Number of effective sequences (N eff) versus number of sequence (N) in the Ovch32 alignments. Dashed line indicates the diagonal. Blue line indicates a linear fit with 95 % confidence intervals in gray. Figure S3. Distribution of C β distances in HisKA-RR interaction (PDB: 3DGE). Figure S4. Distribution of C β distances in Ovch32 interactions [67] (See supplemental file for PDB accessions). Figure S5. Ovch32. Precision (PPV) versus Neff at FPR < 0.1 %. Blue lines indicate a loess fit to each method, 95 % confidence intervals are shown in gray. Figure S6. Ovch32. Power (TPR) versus Neff at FPR < 5 %. Blue lines indicate a loess fit to each method, 95 % confidence intervals are shown in gray. Figure S7. Ovch32. ϕ max versus Neff. Blue lines indicate a loess fit to each method, 95 % confidence intervals are shown in gray. Figure S8. HisKA-RR alt.. Power (TPR) vs Neff/L at FPR < 5 %. A stricter definition of positives, defined experimentally in [46–48] is used. Blue lines indicate a loess fit to each method, 95 % confidence intervals are shown in gray. Figure S9. HisKA-RR alt.. Power (TPR) vs Neff/L at FPR < 0.1 %. A stricter definition of positives, defined experimentally in [46–48] is used. Blue lines indicate a loess fit to each method, 95 % confidence intervals are shown in gray. Figure S10. HisKA-RR alt.. Precision (PPV) vs Neff/L at FPR < 0.1 %. A stricter definition of positives, defined experimentally in [46–48] is used. Blue lines indicate a loess fit to each method, 95 % confidence intervals are shown in gray. Figure S11. Ovch32. Power (TPR) at FPR < 5 % and Precision (PPV) at FPR < 0.1 % versus Neff/L. Blue lines indicate a loess fit to each method, 95 % confidence intervals are shown in gray. Figure S12 [file 12859_2015_677_MOESM13_ESM.zip › 12859_2015_677_add13/Fig_S25_TPR_Prop_Contacts_at_0.05.pdf]

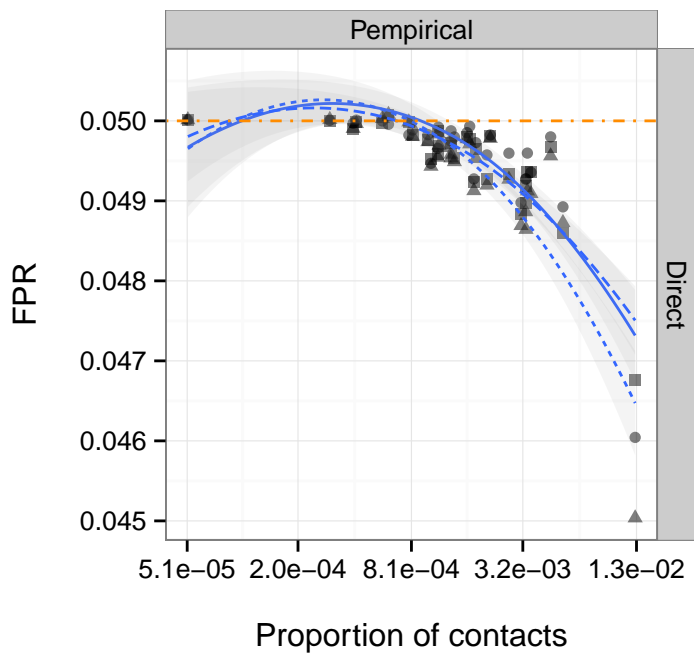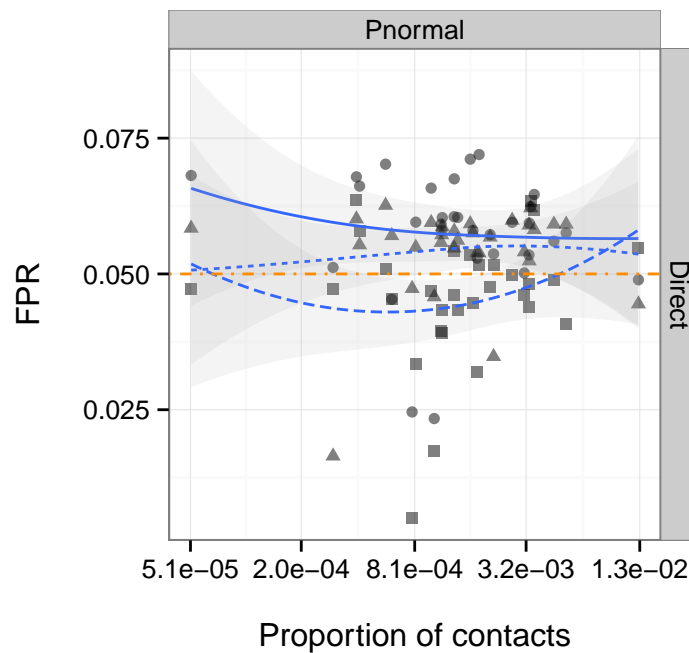

**Method**

- DI
- DIplm
- PSICOV

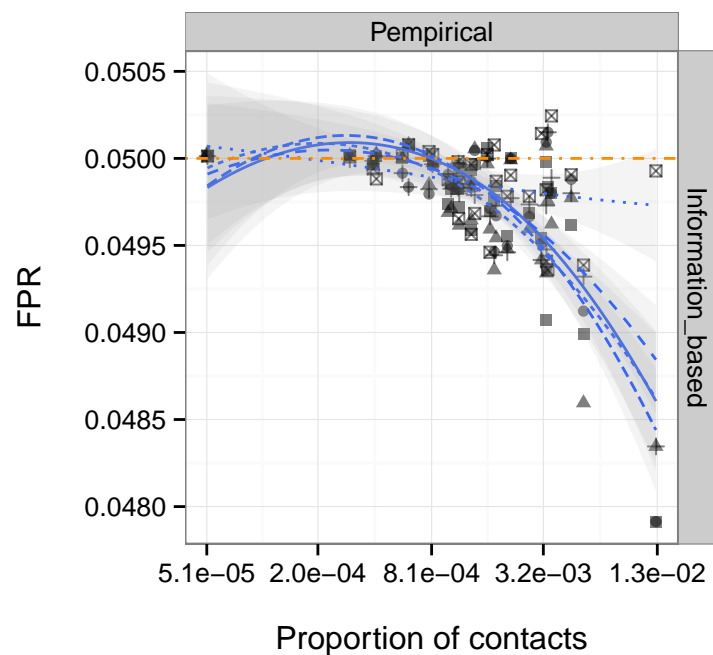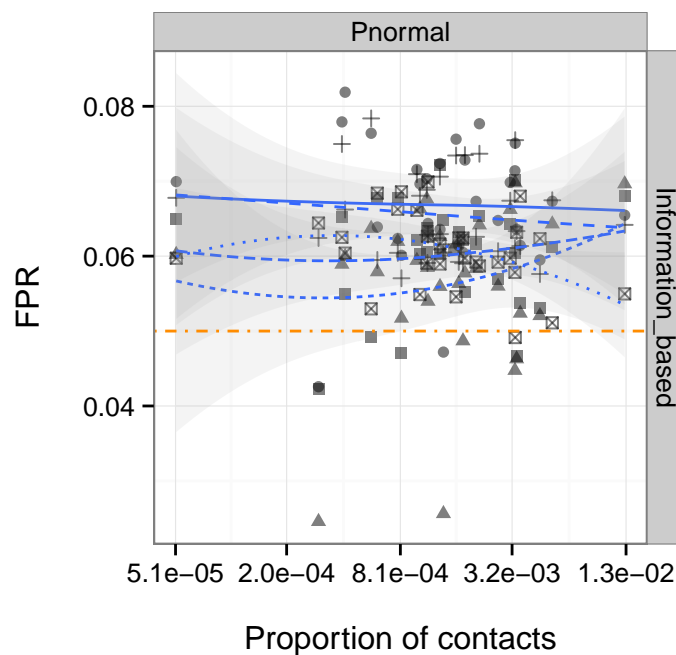

**Method**

- MI
- MIj
- MIminh
- MIw
- VI

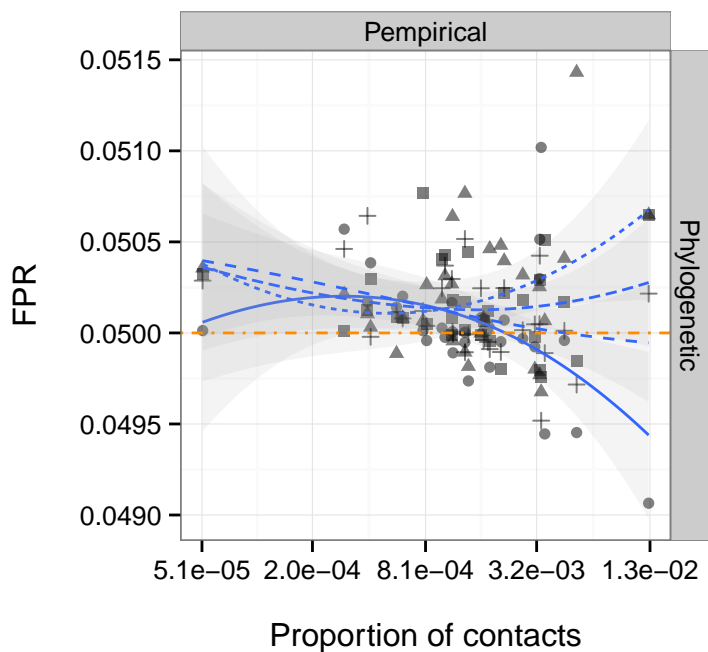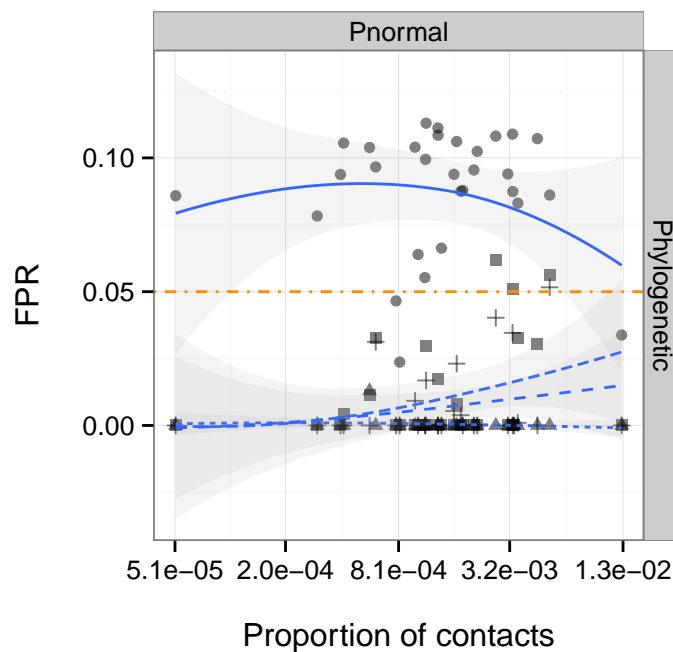

**Method**

- CMPchg
- CMPcor
- CMPpol
- CMPvol

Supplement: Additional file 13 — Figure S1. HisKA-RR. Number of effective sequences (N eff) versus number of sequence (N) in the 60 sub-sampled HisKA-RR alignments. Dashed line indicates the diagonal. Blue line indicates a linear fit with 95 % confidence intervals in gray. Figure S2. Ovch32. Number of effective sequences (N eff) versus number of sequence (N) in the Ovch32 alignments. Dashed line indicates the diagonal. Blue line indicates a linear fit with 95 % confidence intervals in gray. Figure S3. Distribution of C β distances in HisKA-RR interaction (PDB: 3DGE). Figure S4. Distribution of C β distances in Ovch32 interactions [67] (See supplemental file for PDB accessions). Figure S5. Ovch32. Precision (PPV) versus Neff at FPR < 0.1 %. Blue lines indicate a loess fit to each method, 95 % confidence intervals are shown in gray. Figure S6. Ovch32. Power (TPR) versus Neff at FPR < 5 %. Blue lines indicate a loess fit to each method, 95 % confidence intervals are shown in gray. Figure S7. Ovch32. ϕ max versus Neff. Blue lines indicate a loess fit to each method, 95 % confidence intervals are shown in gray. Figure S8. HisKA-RR alt.. Power (TPR) vs Neff/L at FPR < 5 %. A stricter definition of positives, defined experimentally in [46–48] is used. Blue lines indicate a loess fit to each method, 95 % confidence intervals are shown in gray. Figure S9. HisKA-RR alt.. Power (TPR) vs Neff/L at FPR < 0.1 %. A stricter definition of positives, defined experimentally in [46–48] is used. Blue lines indicate a loess fit to each method, 95 % confidence intervals are shown in gray. Figure S10. HisKA-RR alt.. Precision (PPV) vs Neff/L at FPR < 0.1 %. A stricter definition of positives, defined experimentally in [46–48] is used. Blue lines indicate a loess fit to each method, 95 % confidence intervals are shown in gray. Figure S11. Ovch32. Power (TPR) at FPR < 5 % and Precision (PPV) at FPR < 0.1 % versus Neff/L. Blue lines indicate a loess fit to each method, 95 % confidence intervals are shown in gray. Figure S12 [file 12859_2015_677_MOESM13_ESM.zip › 12859_2015_677_add13/Fig_S26_FPR_0.05_Prop_Contacts.pdf]

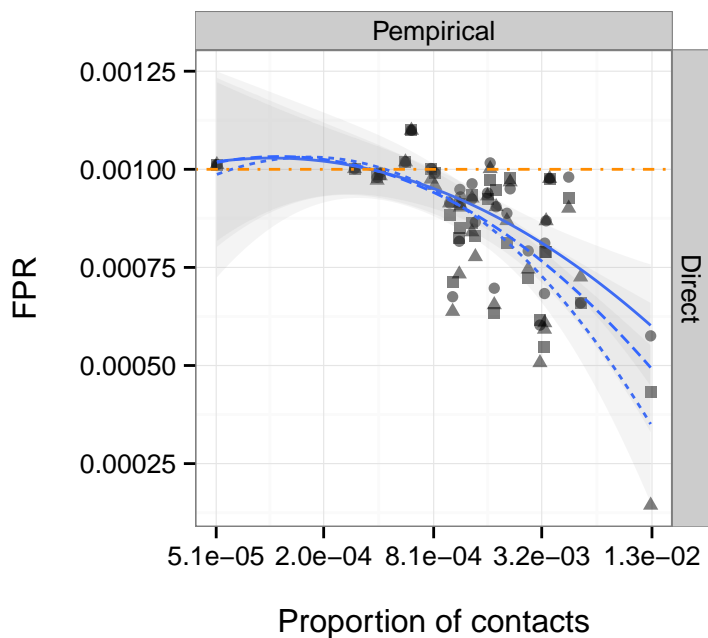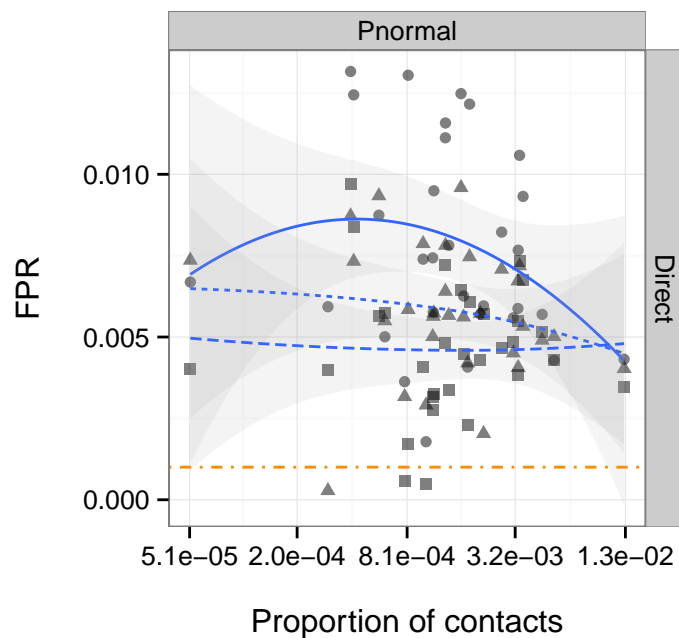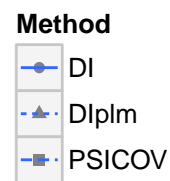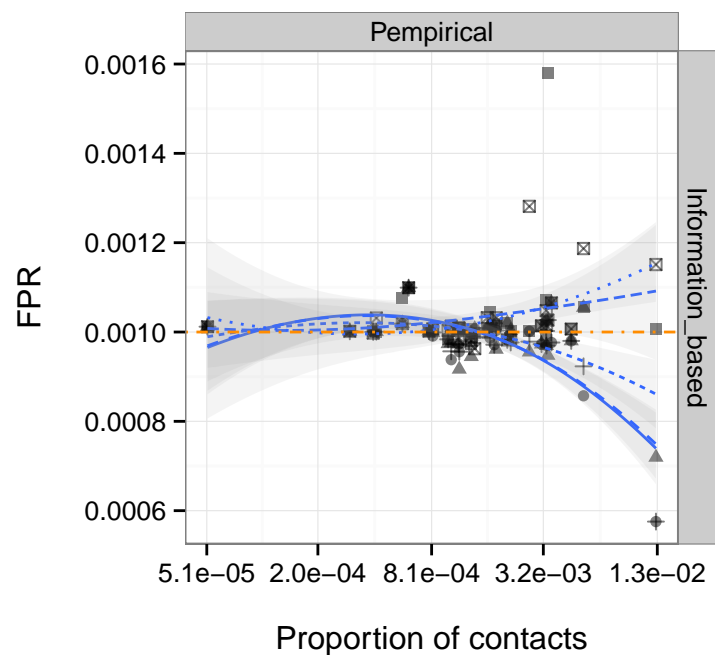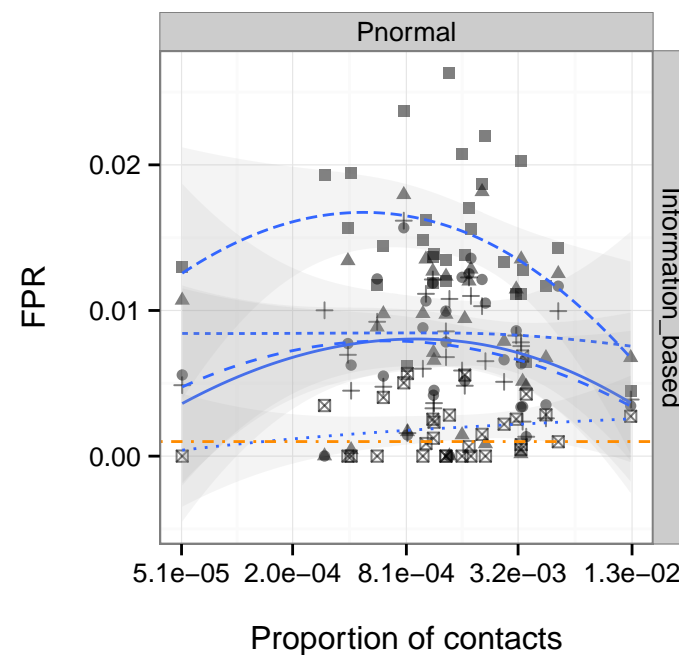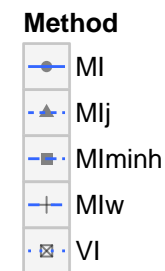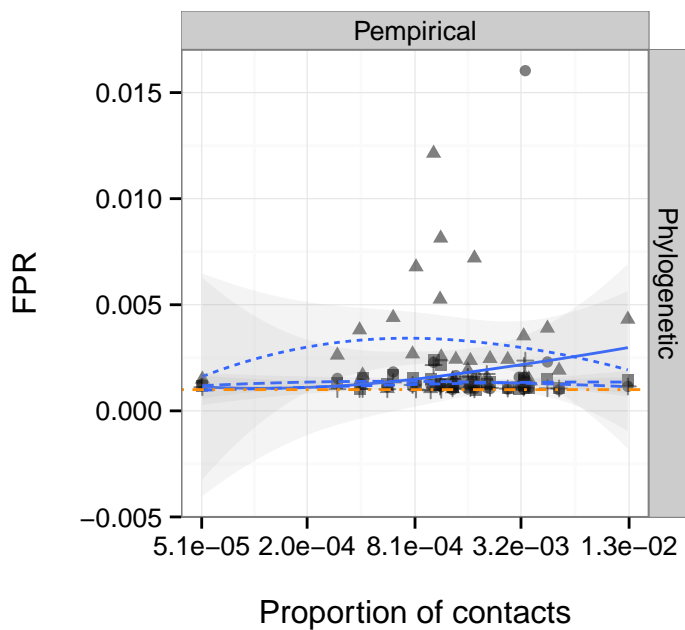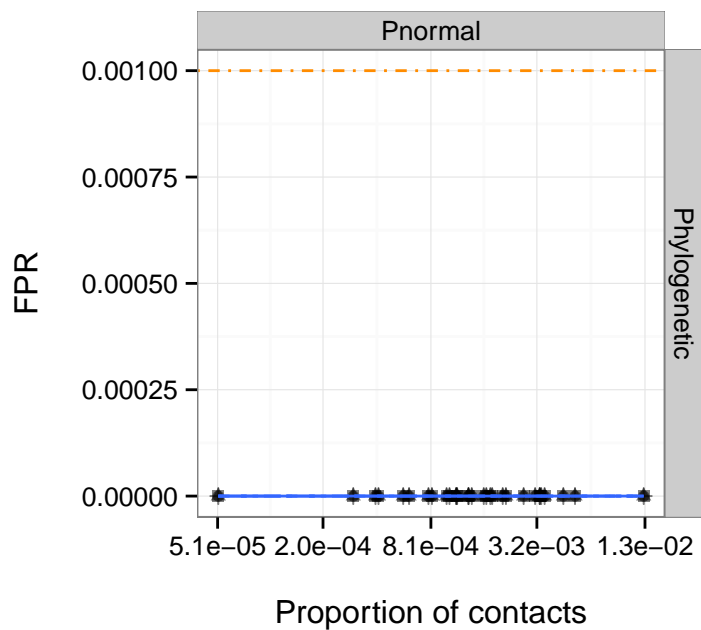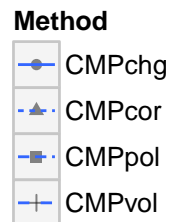

Supplement: Additional file 13 — Figure S1. HisKA-RR. Number of effective sequences (N eff) versus number of sequence (N) in the 60 sub-sampled HisKA-RR alignments. Dashed line indicates the diagonal. Blue line indicates a linear fit with 95 % confidence intervals in gray. Figure S2. Ovch32. Number of effective sequences (N eff) versus number of sequence (N) in the Ovch32 alignments. Dashed line indicates the diagonal. Blue line indicates a linear fit with 95 % confidence intervals in gray. Figure S3. Distribution of C β distances in HisKA-RR interaction (PDB: 3DGE). Figure S4. Distribution of C β distances in Ovch32 interactions [67] (See supplemental file for PDB accessions). Figure S5. Ovch32. Precision (PPV) versus Neff at FPR < 0.1 %. Blue lines indicate a loess fit to each method, 95 % confidence intervals are shown in gray. Figure S6. Ovch32. Power (TPR) versus Neff at FPR < 5 %. Blue lines indicate a loess fit to each method, 95 % confidence intervals are shown in gray. Figure S7. Ovch32. ϕ max versus Neff. Blue lines indicate a loess fit to each method, 95 % confidence intervals are shown in gray. Figure S8. HisKA-RR alt.. Power (TPR) vs Neff/L at FPR < 5 %. A stricter definition of positives, defined experimentally in [46–48] is used. Blue lines indicate a loess fit to each method, 95 % confidence intervals are shown in gray. Figure S9. HisKA-RR alt.. Power (TPR) vs Neff/L at FPR < 0.1 %. A stricter definition of positives, defined experimentally in [46–48] is used. Blue lines indicate a loess fit to each method, 95 % confidence intervals are shown in gray. Figure S10. HisKA-RR alt.. Precision (PPV) vs Neff/L at FPR < 0.1 %. A stricter definition of positives, defined experimentally in [46–48] is used. Blue lines indicate a loess fit to each method, 95 % confidence intervals are shown in gray. Figure S11. Ovch32. Power (TPR) at FPR < 5 % and Precision (PPV) at FPR < 0.1 % versus Neff/L. Blue lines indicate a loess fit to each method, 95 % confidence intervals are shown in gray. Figure S12 [file 12859_2015_677_MOESM13_ESM.zip › 12859_2015_677_add13/Fig_S27_FPR_0.001_Prop_Contacts.pdf]

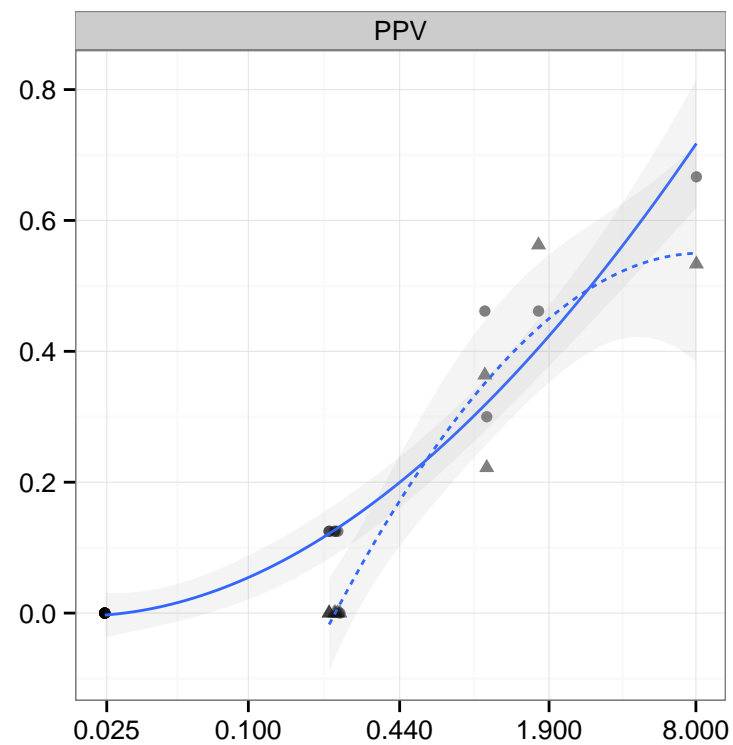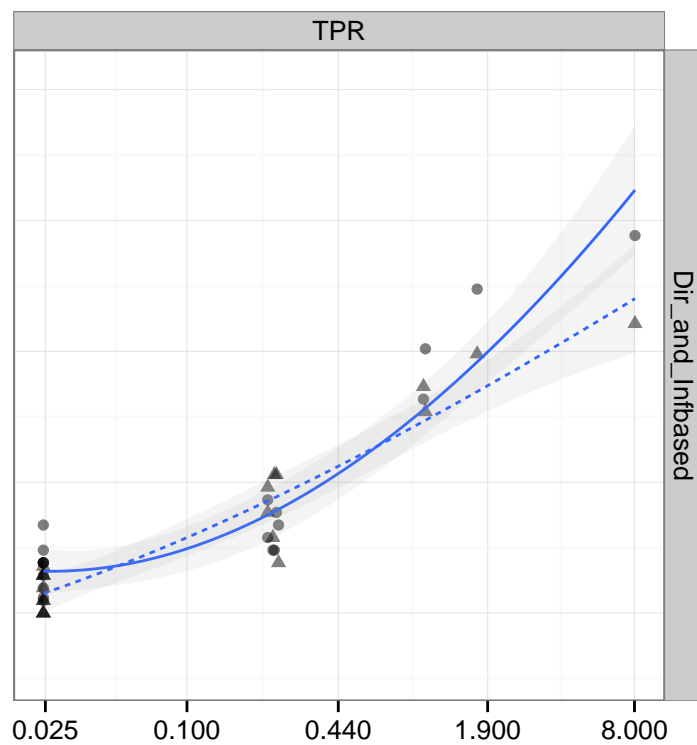

**Method**

- DPlm
- MI

Dir\_and\_Inbaised

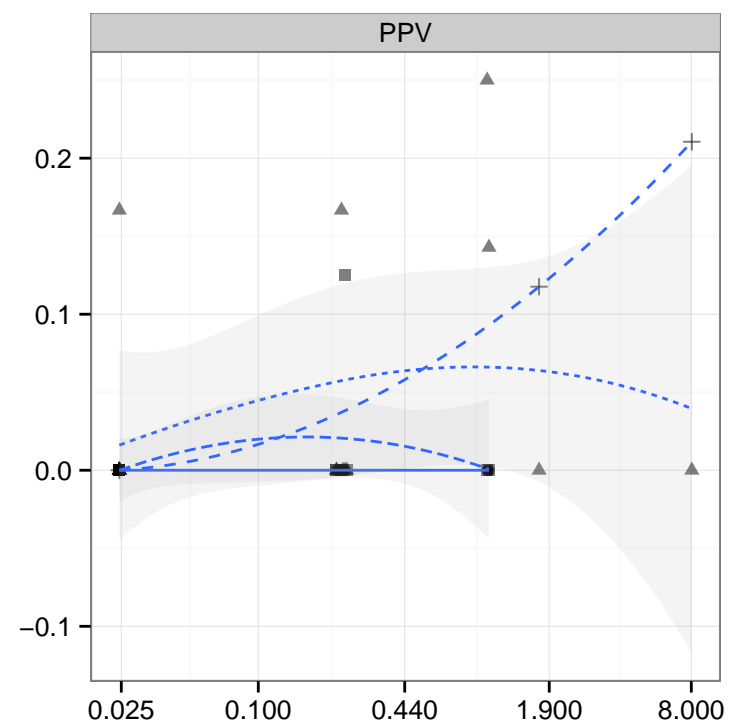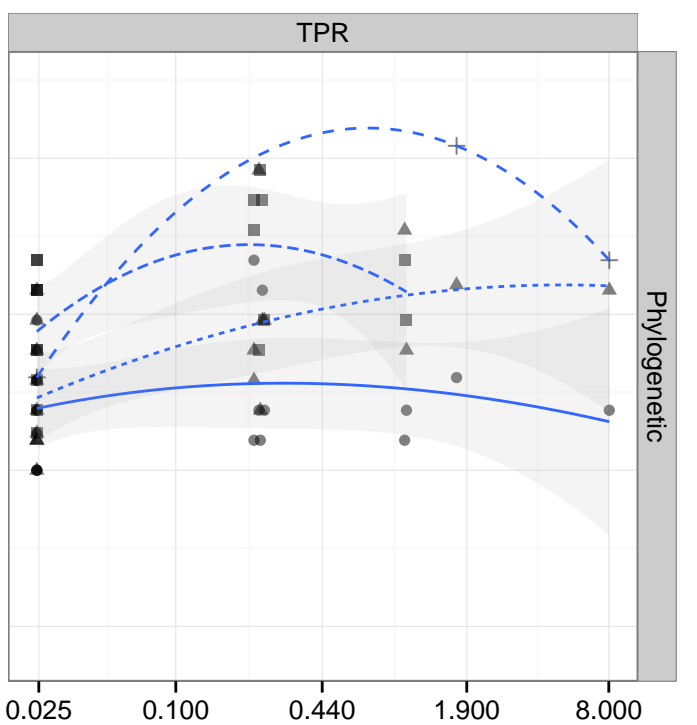

**Method**

- CMPcor
- CMPvol
- CTMP
- Spider

Phylogenetic

Neff/L

Supplement: Additional file 13 — Figure S1. HisKA-RR. Number of effective sequences (N eff) versus number of sequence (N) in the 60 sub-sampled HisKA-RR alignments. Dashed line indicates the diagonal. Blue line indicates a linear fit with 95 % confidence intervals in gray. Figure S2. Ovch32. Number of effective sequences (N eff) versus number of sequence (N) in the Ovch32 alignments. Dashed line indicates the diagonal. Blue line indicates a linear fit with 95 % confidence intervals in gray. Figure S3. Distribution of C β distances in HisKA-RR interaction (PDB: 3DGE). Figure S4. Distribution of C β distances in Ovch32 interactions [67] (See supplemental file for PDB accessions). Figure S5. Ovch32. Precision (PPV) versus Neff at FPR < 0.1 %. Blue lines indicate a loess fit to each method, 95 % confidence intervals are shown in gray. Figure S6. Ovch32. Power (TPR) versus Neff at FPR < 5 %. Blue lines indicate a loess fit to each method, 95 % confidence intervals are shown in gray. Figure S7. Ovch32. ϕ max versus Neff. Blue lines indicate a loess fit to each method, 95 % confidence intervals are shown in gray. Figure S8. HisKA-RR alt.. Power (TPR) vs Neff/L at FPR < 5 %. A stricter definition of positives, defined experimentally in [46–48] is used. Blue lines indicate a loess fit to each method, 95 % confidence intervals are shown in gray. Figure S9. HisKA-RR alt.. Power (TPR) vs Neff/L at FPR < 0.1 %. A stricter definition of positives, defined experimentally in [46–48] is used. Blue lines indicate a loess fit to each method, 95 % confidence intervals are shown in gray. Figure S10. HisKA-RR alt.. Precision (PPV) vs Neff/L at FPR < 0.1 %. A stricter definition of positives, defined experimentally in [46–48] is used. Blue lines indicate a loess fit to each method, 95 % confidence intervals are shown in gray. Figure S11. Ovch32. Power (TPR) at FPR < 5 % and Precision (PPV) at FPR < 0.1 % versus Neff/L. Blue lines indicate a loess fit to each method, 95 % confidence intervals are shown in gray. Figure S12 [file 12859_2015_677_MOESM13_ESM.zip › 12859_2015_677_add13/Fig_S28_TPR5_PPV01_Neff_per_col.pdf]

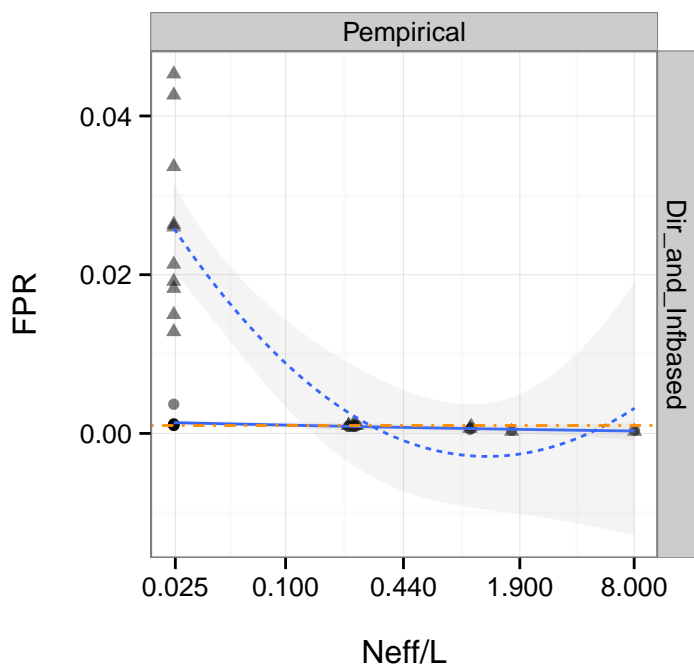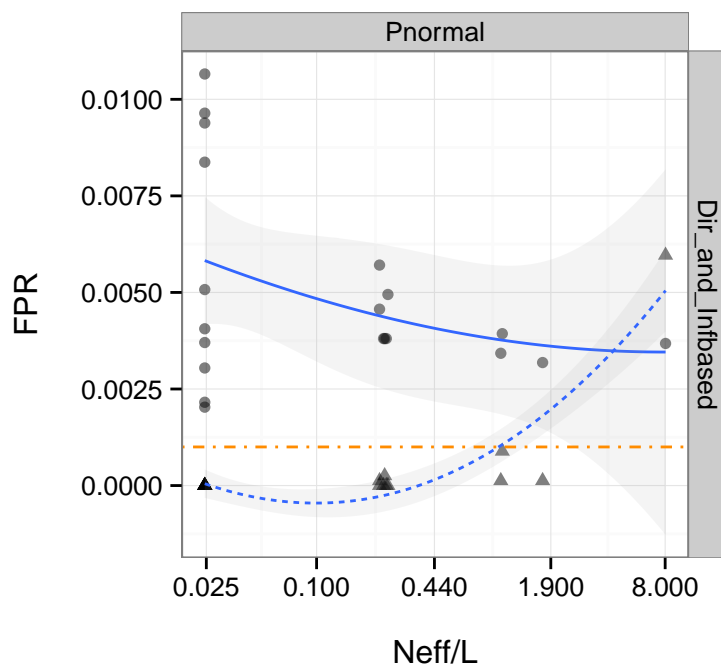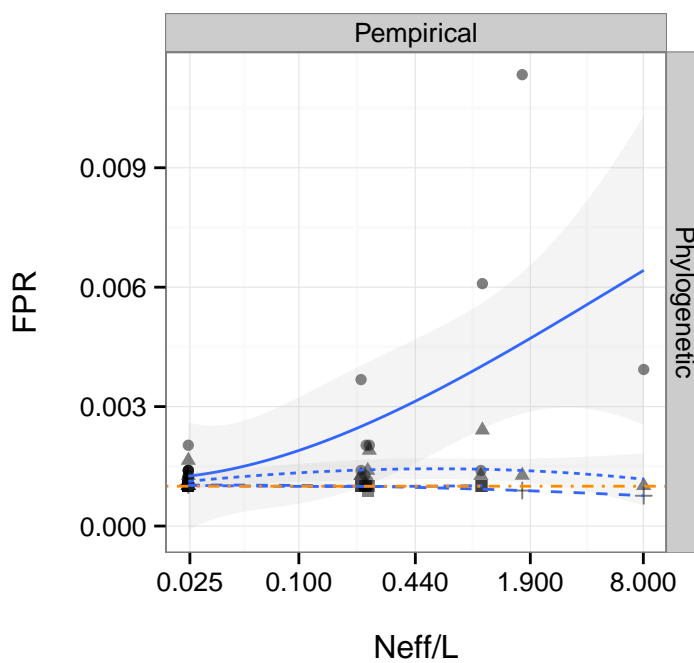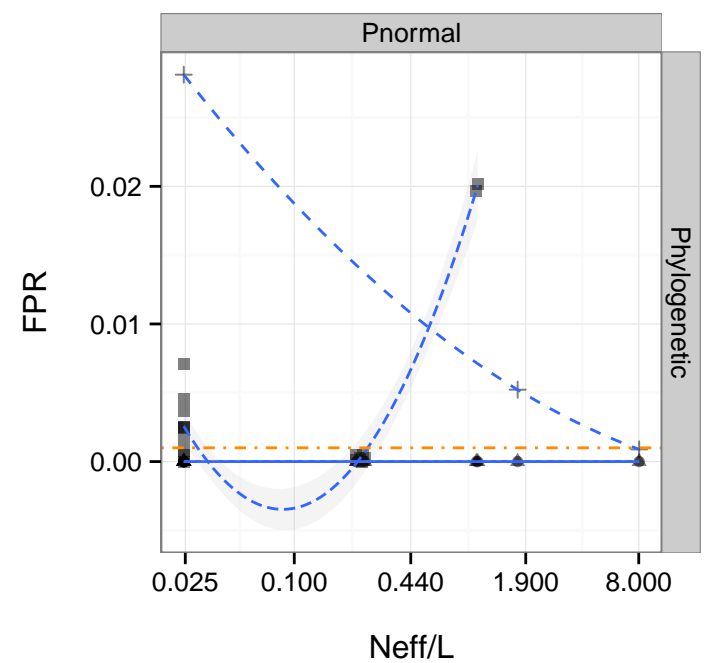

Supplement: Additional file 13 — Figure S1. HisKA-RR. Number of effective sequences (N eff) versus number of sequence (N) in the 60 sub-sampled HisKA-RR alignments. Dashed line indicates the diagonal. Blue line indicates a linear fit with 95 % confidence intervals in gray. Figure S2. Ovch32. Number of effective sequences (N eff) versus number of sequence (N) in the Ovch32 alignments. Dashed line indicates the diagonal. Blue line indicates a linear fit with 95 % confidence intervals in gray. Figure S3. Distribution of C β distances in HisKA-RR interaction (PDB: 3DGE). Figure S4. Distribution of C β distances in Ovch32 interactions [67] (See supplemental file for PDB accessions). Figure S5. Ovch32. Precision (PPV) versus Neff at FPR < 0.1 %. Blue lines indicate a loess fit to each method, 95 % confidence intervals are shown in gray. Figure S6. Ovch32. Power (TPR) versus Neff at FPR < 5 %. Blue lines indicate a loess fit to each method, 95 % confidence intervals are shown in gray. Figure S7. Ovch32. ϕ max versus Neff. Blue lines indicate a loess fit to each method, 95 % confidence intervals are shown in gray. Figure S8. HisKA-RR alt.. Power (TPR) vs Neff/L at FPR < 5 %. A stricter definition of positives, defined experimentally in [46–48] is used. Blue lines indicate a loess fit to each method, 95 % confidence intervals are shown in gray. Figure S9. HisKA-RR alt.. Power (TPR) vs Neff/L at FPR < 0.1 %. A stricter definition of positives, defined experimentally in [46–48] is used. Blue lines indicate a loess fit to each method, 95 % confidence intervals are shown in gray. Figure S10. HisKA-RR alt.. Precision (PPV) vs Neff/L at FPR < 0.1 %. A stricter definition of positives, defined experimentally in [46–48] is used. Blue lines indicate a loess fit to each method, 95 % confidence intervals are shown in gray. Figure S11. Ovch32. Power (TPR) at FPR < 5 % and Precision (PPV) at FPR < 0.1 % versus Neff/L. Blue lines indicate a loess fit to each method, 95 % confidence intervals are shown in gray. Figure S12 [file 12859_2015_677_MOESM13_ESM.zip › 12859_2015_677_add13/Fig_S29_FPR_0.001_Neff_per_col.pdf]

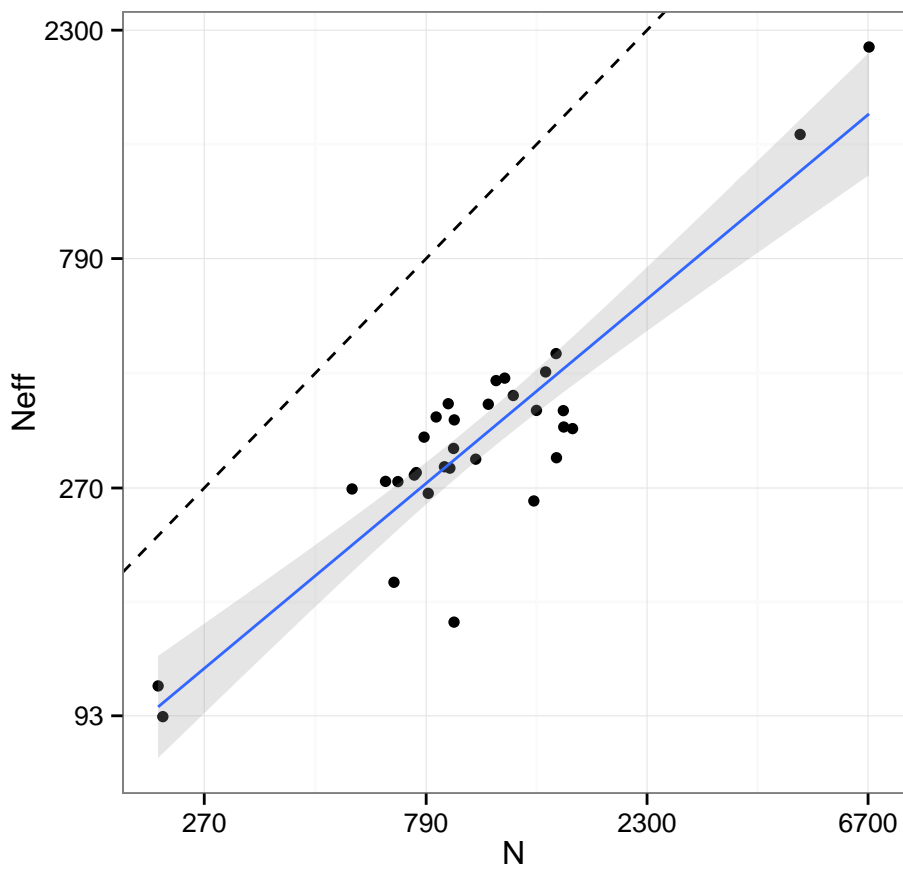

Supplement: Additional file 13 — Figure S1. HisKA-RR. Number of effective sequences (N eff) versus number of sequence (N) in the 60 sub-sampled HisKA-RR alignments. Dashed line indicates the diagonal. Blue line indicates a linear fit with 95 % confidence intervals in gray. Figure S2. Ovch32. Number of effective sequences (N eff) versus number of sequence (N) in the Ovch32 alignments. Dashed line indicates the diagonal. Blue line indicates a linear fit with 95 % confidence intervals in gray. Figure S3. Distribution of C β distances in HisKA-RR interaction (PDB: 3DGE). Figure S4. Distribution of C β distances in Ovch32 interactions [67] (See supplemental file for PDB accessions). Figure S5. Ovch32. Precision (PPV) versus Neff at FPR < 0.1 %. Blue lines indicate a loess fit to each method, 95 % confidence intervals are shown in gray. Figure S6. Ovch32. Power (TPR) versus Neff at FPR < 5 %. Blue lines indicate a loess fit to each method, 95 % confidence intervals are shown in gray. Figure S7. Ovch32. ϕ max versus Neff. Blue lines indicate a loess fit to each method, 95 % confidence intervals are shown in gray. Figure S8. HisKA-RR alt.. Power (TPR) vs Neff/L at FPR < 5 %. A stricter definition of positives, defined experimentally in [46–48] is used. Blue lines indicate a loess fit to each method, 95 % confidence intervals are shown in gray. Figure S9. HisKA-RR alt.. Power (TPR) vs Neff/L at FPR < 0.1 %. A stricter definition of positives, defined experimentally in [46–48] is used. Blue lines indicate a loess fit to each method, 95 % confidence intervals are shown in gray. Figure S10. HisKA-RR alt.. Precision (PPV) vs Neff/L at FPR < 0.1 %. A stricter definition of positives, defined experimentally in [46–48] is used. Blue lines indicate a loess fit to each method, 95 % confidence intervals are shown in gray. Figure S11. Ovch32. Power (TPR) at FPR < 5 % and Precision (PPV) at FPR < 0.1 % versus Neff/L. Blue lines indicate a loess fit to each method, 95 % confidence intervals are shown in gray. Figure S12 [file 12859_2015_677_MOESM13_ESM.zip › 12859_2015_677_add13/Fig_S2_nneff.pdf]

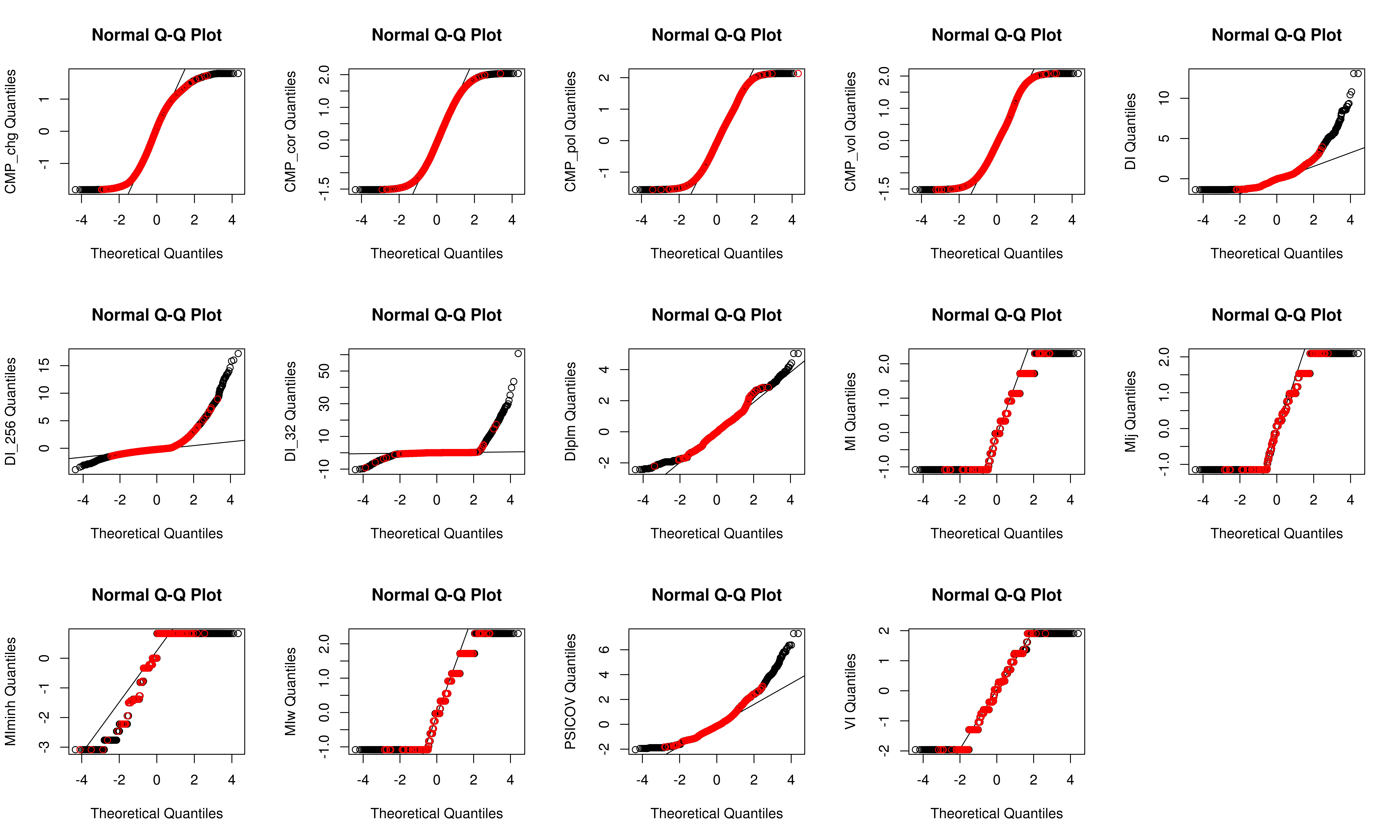

Supplement: Additional file 13 — Figure S1. HisKA-RR. Number of effective sequences (N eff) versus number of sequence (N) in the 60 sub-sampled HisKA-RR alignments. Dashed line indicates the diagonal. Blue line indicates a linear fit with 95 % confidence intervals in gray. Figure S2. Ovch32. Number of effective sequences (N eff) versus number of sequence (N) in the Ovch32 alignments. Dashed line indicates the diagonal. Blue line indicates a linear fit with 95 % confidence intervals in gray. Figure S3. Distribution of C β distances in HisKA-RR interaction (PDB: 3DGE). Figure S4. Distribution of C β distances in Ovch32 interactions [67] (See supplemental file for PDB accessions). Figure S5. Ovch32. Precision (PPV) versus Neff at FPR < 0.1 %. Blue lines indicate a loess fit to each method, 95 % confidence intervals are shown in gray. Figure S6. Ovch32. Power (TPR) versus Neff at FPR < 5 %. Blue lines indicate a loess fit to each method, 95 % confidence intervals are shown in gray. Figure S7. Ovch32. ϕ max versus Neff. Blue lines indicate a loess fit to each method, 95 % confidence intervals are shown in gray. Figure S8. HisKA-RR alt.. Power (TPR) vs Neff/L at FPR < 5 %. A stricter definition of positives, defined experimentally in [46–48] is used. Blue lines indicate a loess fit to each method, 95 % confidence intervals are shown in gray. Figure S9. HisKA-RR alt.. Power (TPR) vs Neff/L at FPR < 0.1 %. A stricter definition of positives, defined experimentally in [46–48] is used. Blue lines indicate a loess fit to each method, 95 % confidence intervals are shown in gray. Figure S10. HisKA-RR alt.. Precision (PPV) vs Neff/L at FPR < 0.1 %. A stricter definition of positives, defined experimentally in [46–48] is used. Blue lines indicate a loess fit to each method, 95 % confidence intervals are shown in gray. Figure S11. Ovch32. Power (TPR) at FPR < 5 % and Precision (PPV) at FPR < 0.1 % versus Neff/L. Blue lines indicate a loess fit to each method, 95 % confidence intervals are shown in gray. Figure S12 [file 12859_2015_677_MOESM13_ESM.zip › 12859_2015_677_add13/Fig_S30_QQ_5_all.png]

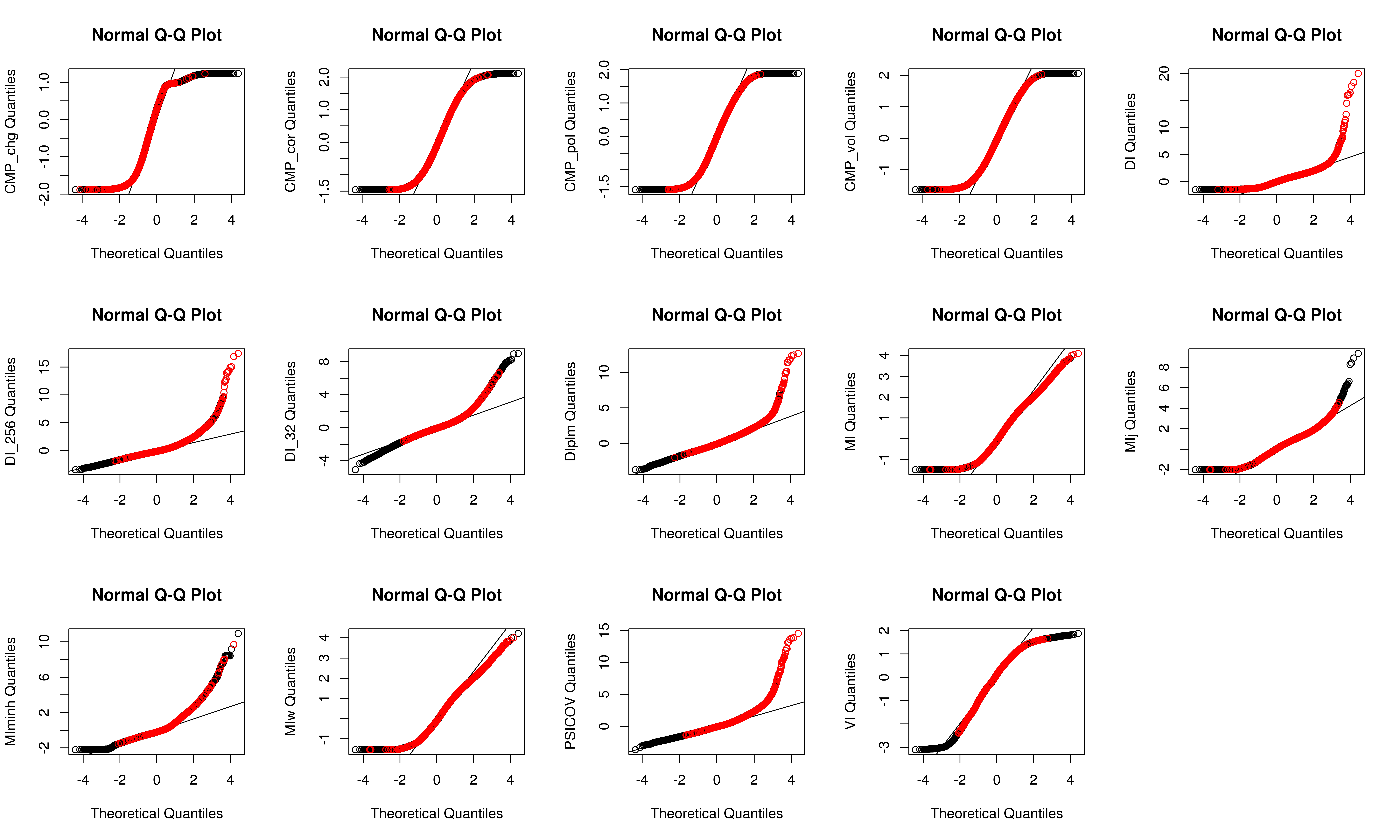

Supplement: Additional file 13 — Figure S1. HisKA-RR. Number of effective sequences (N eff) versus number of sequence (N) in the 60 sub-sampled HisKA-RR alignments. Dashed line indicates the diagonal. Blue line indicates a linear fit with 95 % confidence intervals in gray. Figure S2. Ovch32. Number of effective sequences (N eff) versus number of sequence (N) in the Ovch32 alignments. Dashed line indicates the diagonal. Blue line indicates a linear fit with 95 % confidence intervals in gray. Figure S3. Distribution of C β distances in HisKA-RR interaction (PDB: 3DGE). Figure S4. Distribution of C β distances in Ovch32 interactions [67] (See supplemental file for PDB accessions). Figure S5. Ovch32. Precision (PPV) versus Neff at FPR < 0.1 %. Blue lines indicate a loess fit to each method, 95 % confidence intervals are shown in gray. Figure S6. Ovch32. Power (TPR) versus Neff at FPR < 5 %. Blue lines indicate a loess fit to each method, 95 % confidence intervals are shown in gray. Figure S7. Ovch32. ϕ max versus Neff. Blue lines indicate a loess fit to each method, 95 % confidence intervals are shown in gray. Figure S8. HisKA-RR alt.. Power (TPR) vs Neff/L at FPR < 5 %. A stricter definition of positives, defined experimentally in [46–48] is used. Blue lines indicate a loess fit to each method, 95 % confidence intervals are shown in gray. Figure S9. HisKA-RR alt.. Power (TPR) vs Neff/L at FPR < 0.1 %. A stricter definition of positives, defined experimentally in [46–48] is used. Blue lines indicate a loess fit to each method, 95 % confidence intervals are shown in gray. Figure S10. HisKA-RR alt.. Precision (PPV) vs Neff/L at FPR < 0.1 %. A stricter definition of positives, defined experimentally in [46–48] is used. Blue lines indicate a loess fit to each method, 95 % confidence intervals are shown in gray. Figure S11. Ovch32. Power (TPR) at FPR < 5 % and Precision (PPV) at FPR < 0.1 % versus Neff/L. Blue lines indicate a loess fit to each method, 95 % confidence intervals are shown in gray. Figure S12 [file 12859_2015_677_MOESM13_ESM.zip › 12859_2015_677_add13/Fig_S31_QQ_500_all.png]

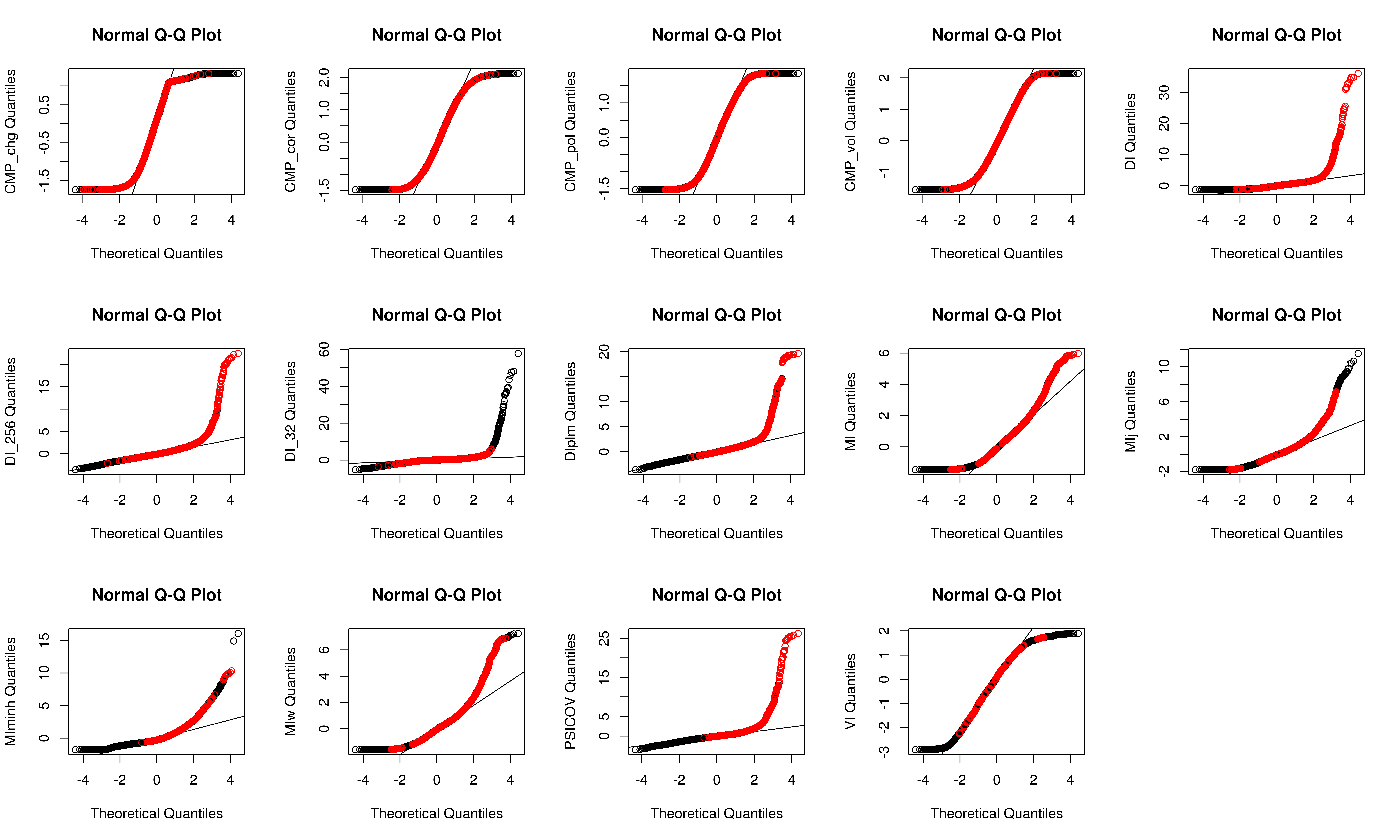

Supplement: Additional file 13 — Figure S1. HisKA-RR. Number of effective sequences (N eff) versus number of sequence (N) in the 60 sub-sampled HisKA-RR alignments. Dashed line indicates the diagonal. Blue line indicates a linear fit with 95 % confidence intervals in gray. Figure S2. Ovch32. Number of effective sequences (N eff) versus number of sequence (N) in the Ovch32 alignments. Dashed line indicates the diagonal. Blue line indicates a linear fit with 95 % confidence intervals in gray. Figure S3. Distribution of C β distances in HisKA-RR interaction (PDB: 3DGE). Figure S4. Distribution of C β distances in Ovch32 interactions [67] (See supplemental file for PDB accessions). Figure S5. Ovch32. Precision (PPV) versus Neff at FPR < 0.1 %. Blue lines indicate a loess fit to each method, 95 % confidence intervals are shown in gray. Figure S6. Ovch32. Power (TPR) versus Neff at FPR < 5 %. Blue lines indicate a loess fit to each method, 95 % confidence intervals are shown in gray. Figure S7. Ovch32. ϕ max versus Neff. Blue lines indicate a loess fit to each method, 95 % confidence intervals are shown in gray. Figure S8. HisKA-RR alt.. Power (TPR) vs Neff/L at FPR < 5 %. A stricter definition of positives, defined experimentally in [46–48] is used. Blue lines indicate a loess fit to each method, 95 % confidence intervals are shown in gray. Figure S9. HisKA-RR alt.. Power (TPR) vs Neff/L at FPR < 0.1 %. A stricter definition of positives, defined experimentally in [46–48] is used. Blue lines indicate a loess fit to each method, 95 % confidence intervals are shown in gray. Figure S10. HisKA-RR alt.. Precision (PPV) vs Neff/L at FPR < 0.1 %. A stricter definition of positives, defined experimentally in [46–48] is used. Blue lines indicate a loess fit to each method, 95 % confidence intervals are shown in gray. Figure S11. Ovch32. Power (TPR) at FPR < 5 % and Precision (PPV) at FPR < 0.1 % versus Neff/L. Blue lines indicate a loess fit to each method, 95 % confidence intervals are shown in gray. Figure S12 [file 12859_2015_677_MOESM13_ESM.zip › 12859_2015_677_add13/Fig_S32_QQ_5000_all.png]

# Bootstrap null on "well-simulated" column pairs

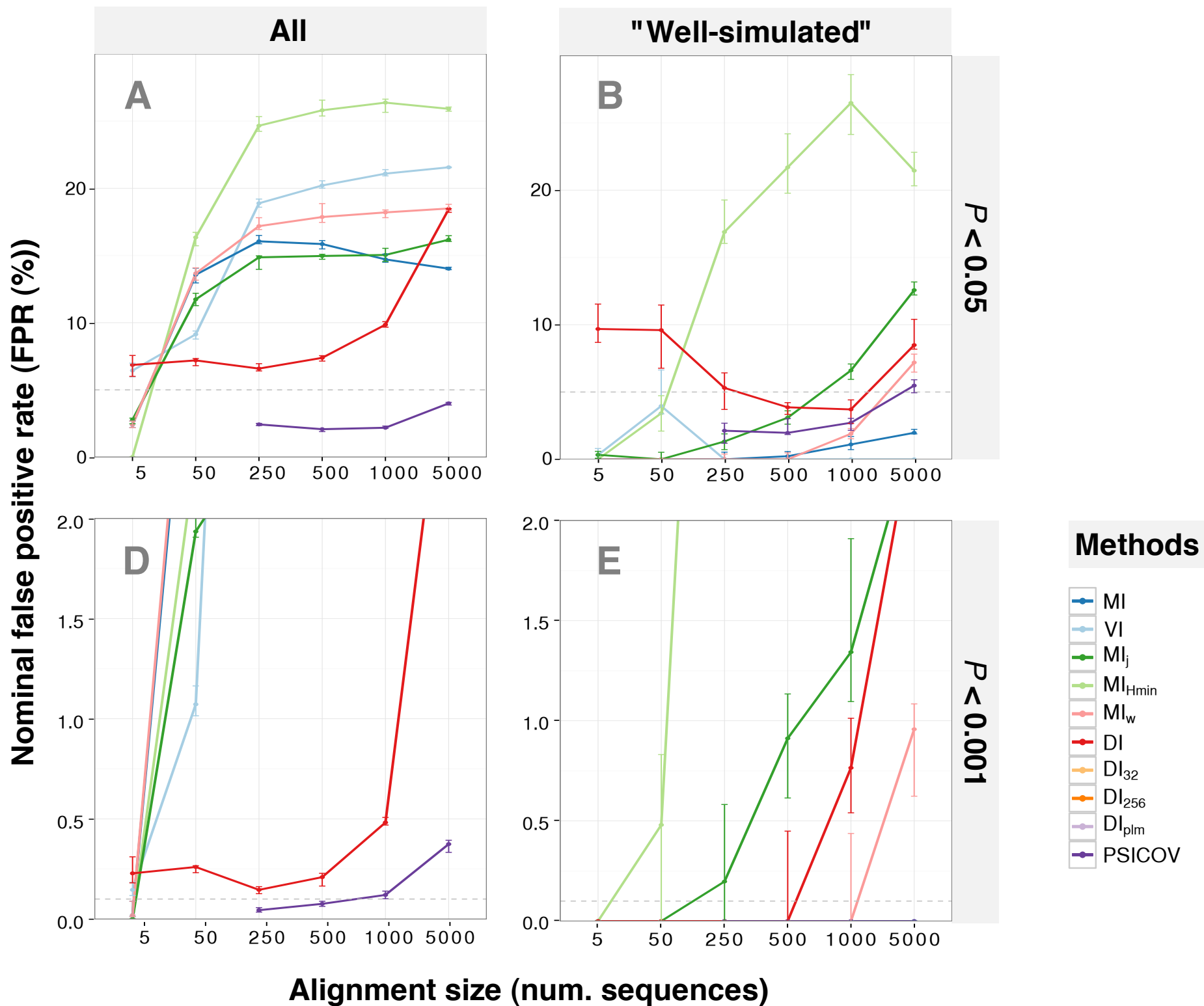

Supplement: Additional file 13 — Figure S1. HisKA-RR. Number of effective sequences (N eff) versus number of sequence (N) in the 60 sub-sampled HisKA-RR alignments. Dashed line indicates the diagonal. Blue line indicates a linear fit with 95 % confidence intervals in gray. Figure S2. Ovch32. Number of effective sequences (N eff) versus number of sequence (N) in the Ovch32 alignments. Dashed line indicates the diagonal. Blue line indicates a linear fit with 95 % confidence intervals in gray. Figure S3. Distribution of C β distances in HisKA-RR interaction (PDB: 3DGE). Figure S4. Distribution of C β distances in Ovch32 interactions [67] (See supplemental file for PDB accessions). Figure S5. Ovch32. Precision (PPV) versus Neff at FPR < 0.1 %. Blue lines indicate a loess fit to each method, 95 % confidence intervals are shown in gray. Figure S6. Ovch32. Power (TPR) versus Neff at FPR < 5 %. Blue lines indicate a loess fit to each method, 95 % confidence intervals are shown in gray. Figure S7. Ovch32. ϕ max versus Neff. Blue lines indicate a loess fit to each method, 95 % confidence intervals are shown in gray. Figure S8. HisKA-RR alt.. Power (TPR) vs Neff/L at FPR < 5 %. A stricter definition of positives, defined experimentally in [46–48] is used. Blue lines indicate a loess fit to each method, 95 % confidence intervals are shown in gray. Figure S9. HisKA-RR alt.. Power (TPR) vs Neff/L at FPR < 0.1 %. A stricter definition of positives, defined experimentally in [46–48] is used. Blue lines indicate a loess fit to each method, 95 % confidence intervals are shown in gray. Figure S10. HisKA-RR alt.. Precision (PPV) vs Neff/L at FPR < 0.1 %. A stricter definition of positives, defined experimentally in [46–48] is used. Blue lines indicate a loess fit to each method, 95 % confidence intervals are shown in gray. Figure S11. Ovch32. Power (TPR) at FPR < 5 % and Precision (PPV) at FPR < 0.1 % versus Neff/L. Blue lines indicate a loess fit to each method, 95 % confidence intervals are shown in gray. Figure S12 [file 12859_2015_677_MOESM13_ESM.zip › 12859_2015_677_add13/Fig_S33_FigureS8_GoodPboot_FPR_at_alpha.pdf]

# PPV-optimized performance

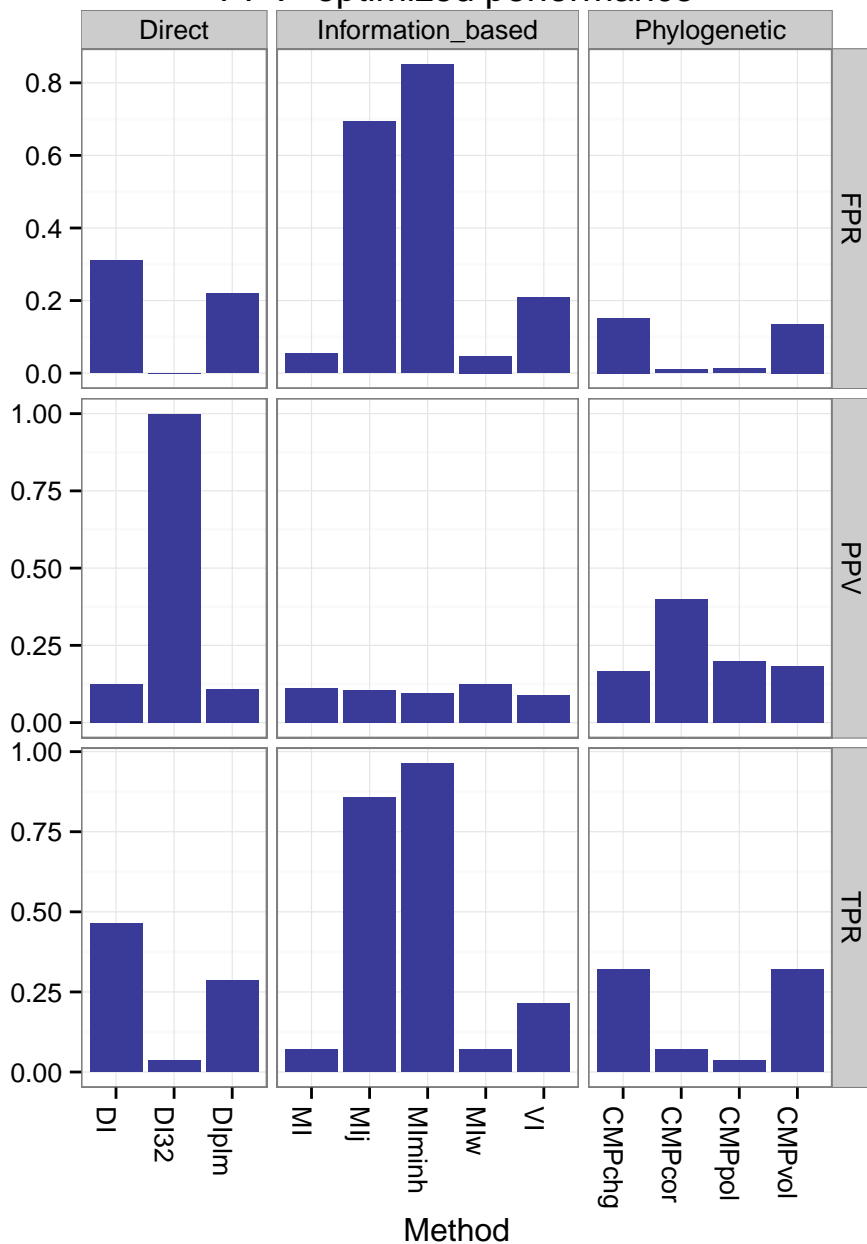

Supplement: Additional file 13 — Figure S1. HisKA-RR. Number of effective sequences (N eff) versus number of sequence (N) in the 60 sub-sampled HisKA-RR alignments. Dashed line indicates the diagonal. Blue line indicates a linear fit with 95 % confidence intervals in gray. Figure S2. Ovch32. Number of effective sequences (N eff) versus number of sequence (N) in the Ovch32 alignments. Dashed line indicates the diagonal. Blue line indicates a linear fit with 95 % confidence intervals in gray. Figure S3. Distribution of C β distances in HisKA-RR interaction (PDB: 3DGE). Figure S4. Distribution of C β distances in Ovch32 interactions [67] (See supplemental file for PDB accessions). Figure S5. Ovch32. Precision (PPV) versus Neff at FPR < 0.1 %. Blue lines indicate a loess fit to each method, 95 % confidence intervals are shown in gray. Figure S6. Ovch32. Power (TPR) versus Neff at FPR < 5 %. Blue lines indicate a loess fit to each method, 95 % confidence intervals are shown in gray. Figure S7. Ovch32. ϕ max versus Neff. Blue lines indicate a loess fit to each method, 95 % confidence intervals are shown in gray. Figure S8. HisKA-RR alt.. Power (TPR) vs Neff/L at FPR < 5 %. A stricter definition of positives, defined experimentally in [46–48] is used. Blue lines indicate a loess fit to each method, 95 % confidence intervals are shown in gray. Figure S9. HisKA-RR alt.. Power (TPR) vs Neff/L at FPR < 0.1 %. A stricter definition of positives, defined experimentally in [46–48] is used. Blue lines indicate a loess fit to each method, 95 % confidence intervals are shown in gray. Figure S10. HisKA-RR alt.. Precision (PPV) vs Neff/L at FPR < 0.1 %. A stricter definition of positives, defined experimentally in [46–48] is used. Blue lines indicate a loess fit to each method, 95 % confidence intervals are shown in gray. Figure S11. Ovch32. Power (TPR) at FPR < 5 % and Precision (PPV) at FPR < 0.1 % versus Neff/L. Blue lines indicate a loess fit to each method, 95 % confidence intervals are shown in gray. Figure S12 [file 12859_2015_677_MOESM13_ESM.zip › 12859_2015_677_add13/Fig_S34_vif.r_flattab__Label_relaxed__perf_NA__ppvOpt.pdf]

# PPV-optimized performance

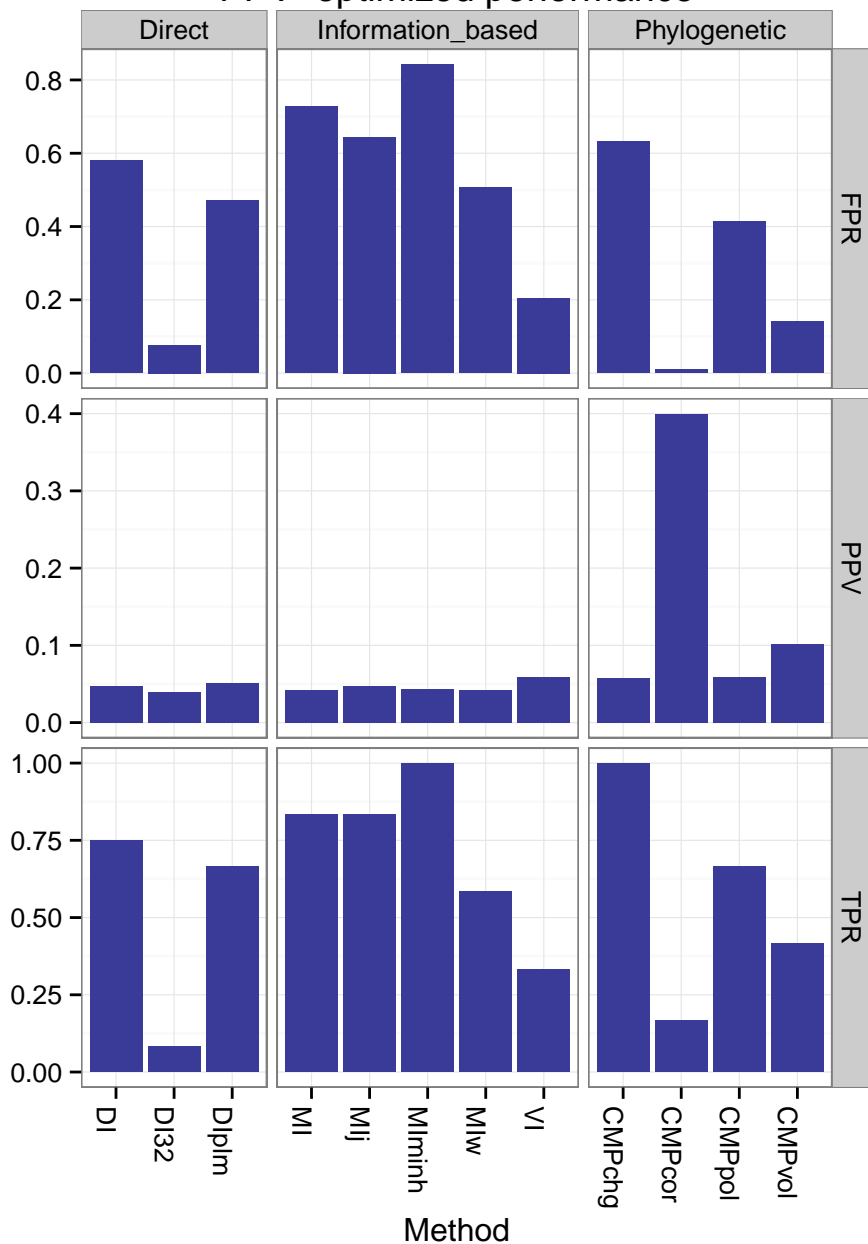

Supplement: Additional file 13 — Figure S1. HisKA-RR. Number of effective sequences (N eff) versus number of sequence (N) in the 60 sub-sampled HisKA-RR alignments. Dashed line indicates the diagonal. Blue line indicates a linear fit with 95 % confidence intervals in gray. Figure S2. Ovch32. Number of effective sequences (N eff) versus number of sequence (N) in the Ovch32 alignments. Dashed line indicates the diagonal. Blue line indicates a linear fit with 95 % confidence intervals in gray. Figure S3. Distribution of C β distances in HisKA-RR interaction (PDB: 3DGE). Figure S4. Distribution of C β distances in Ovch32 interactions [67] (See supplemental file for PDB accessions). Figure S5. Ovch32. Precision (PPV) versus Neff at FPR < 0.1 %. Blue lines indicate a loess fit to each method, 95 % confidence intervals are shown in gray. Figure S6. Ovch32. Power (TPR) versus Neff at FPR < 5 %. Blue lines indicate a loess fit to each method, 95 % confidence intervals are shown in gray. Figure S7. Ovch32. ϕ max versus Neff. Blue lines indicate a loess fit to each method, 95 % confidence intervals are shown in gray. Figure S8. HisKA-RR alt.. Power (TPR) vs Neff/L at FPR < 5 %. A stricter definition of positives, defined experimentally in [46–48] is used. Blue lines indicate a loess fit to each method, 95 % confidence intervals are shown in gray. Figure S9. HisKA-RR alt.. Power (TPR) vs Neff/L at FPR < 0.1 %. A stricter definition of positives, defined experimentally in [46–48] is used. Blue lines indicate a loess fit to each method, 95 % confidence intervals are shown in gray. Figure S10. HisKA-RR alt.. Precision (PPV) vs Neff/L at FPR < 0.1 %. A stricter definition of positives, defined experimentally in [46–48] is used. Blue lines indicate a loess fit to each method, 95 % confidence intervals are shown in gray. Figure S11. Ovch32. Power (TPR) at FPR < 5 % and Precision (PPV) at FPR < 0.1 % versus Neff/L. Blue lines indicate a loess fit to each method, 95 % confidence intervals are shown in gray. Figure S12 [file 12859_2015_677_MOESM13_ESM.zip › 12859_2015_677_add13/Fig_S35_vif.r_flattab__Label_strict__perf_NA__ppvOpt.pdf]

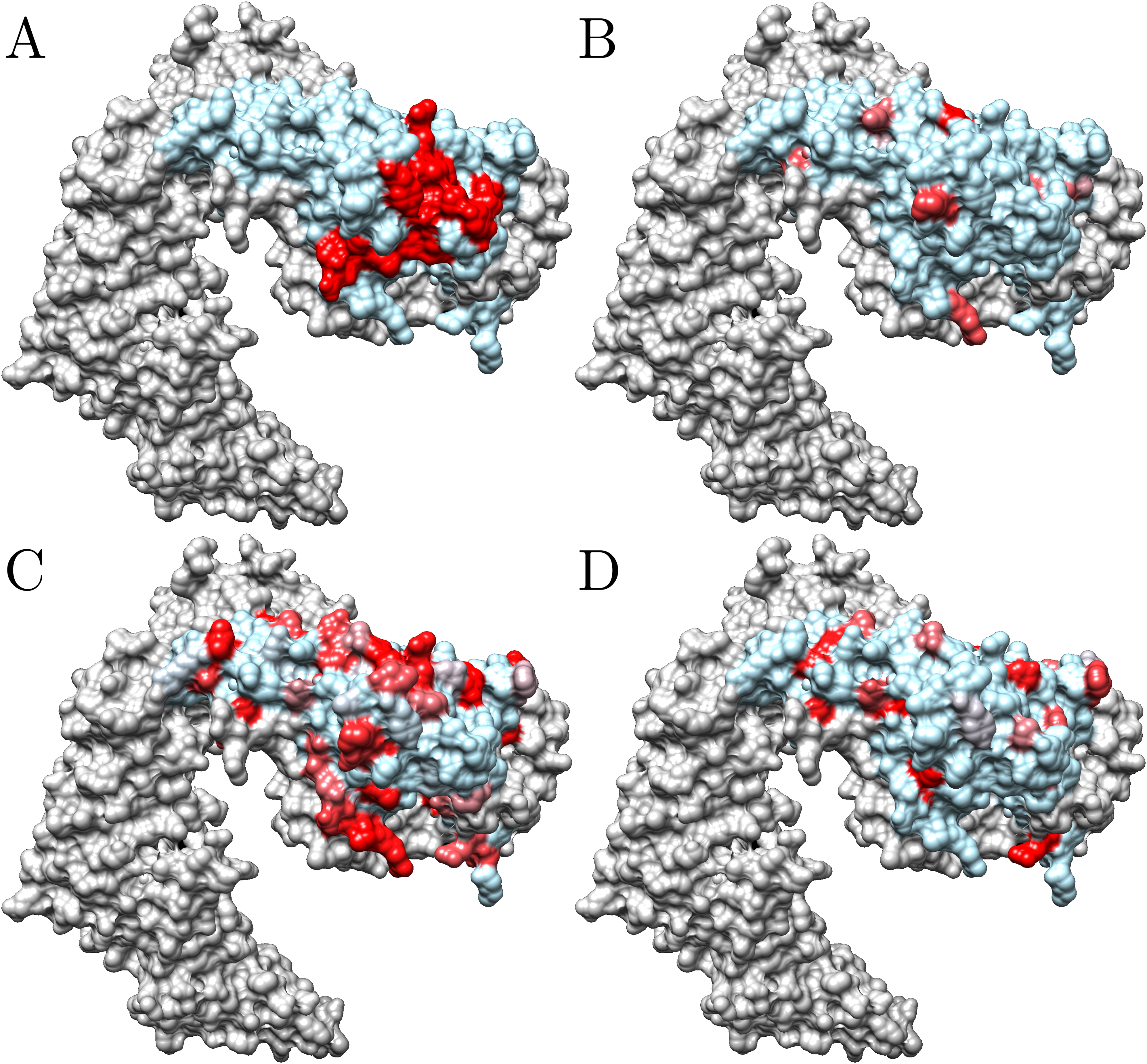

Supplement: Additional file 13 — Figure S1. HisKA-RR. Number of effective sequences (N eff) versus number of sequence (N) in the 60 sub-sampled HisKA-RR alignments. Dashed line indicates the diagonal. Blue line indicates a linear fit with 95 % confidence intervals in gray. Figure S2. Ovch32. Number of effective sequences (N eff) versus number of sequence (N) in the Ovch32 alignments. Dashed line indicates the diagonal. Blue line indicates a linear fit with 95 % confidence intervals in gray. Figure S3. Distribution of C β distances in HisKA-RR interaction (PDB: 3DGE). Figure S4. Distribution of C β distances in Ovch32 interactions [67] (See supplemental file for PDB accessions). Figure S5. Ovch32. Precision (PPV) versus Neff at FPR < 0.1 %. Blue lines indicate a loess fit to each method, 95 % confidence intervals are shown in gray. Figure S6. Ovch32. Power (TPR) versus Neff at FPR < 5 %. Blue lines indicate a loess fit to each method, 95 % confidence intervals are shown in gray. Figure S7. Ovch32. ϕ max versus Neff. Blue lines indicate a loess fit to each method, 95 % confidence intervals are shown in gray. Figure S8. HisKA-RR alt.. Power (TPR) vs Neff/L at FPR < 5 %. A stricter definition of positives, defined experimentally in [46–48] is used. Blue lines indicate a loess fit to each method, 95 % confidence intervals are shown in gray. Figure S9. HisKA-RR alt.. Power (TPR) vs Neff/L at FPR < 0.1 %. A stricter definition of positives, defined experimentally in [46–48] is used. Blue lines indicate a loess fit to each method, 95 % confidence intervals are shown in gray. Figure S10. HisKA-RR alt.. Precision (PPV) vs Neff/L at FPR < 0.1 %. A stricter definition of positives, defined experimentally in [46–48] is used. Blue lines indicate a loess fit to each method, 95 % confidence intervals are shown in gray. Figure S11. Ovch32. Power (TPR) at FPR < 5 % and Precision (PPV) at FPR < 0.1 % versus Neff/L. Blue lines indicate a loess fit to each method, 95 % confidence intervals are shown in gray. Figure S12 [file 12859_2015_677_MOESM13_ESM.zip › 12859_2015_677_add13/Fig_S36_vif_multipanel.png]

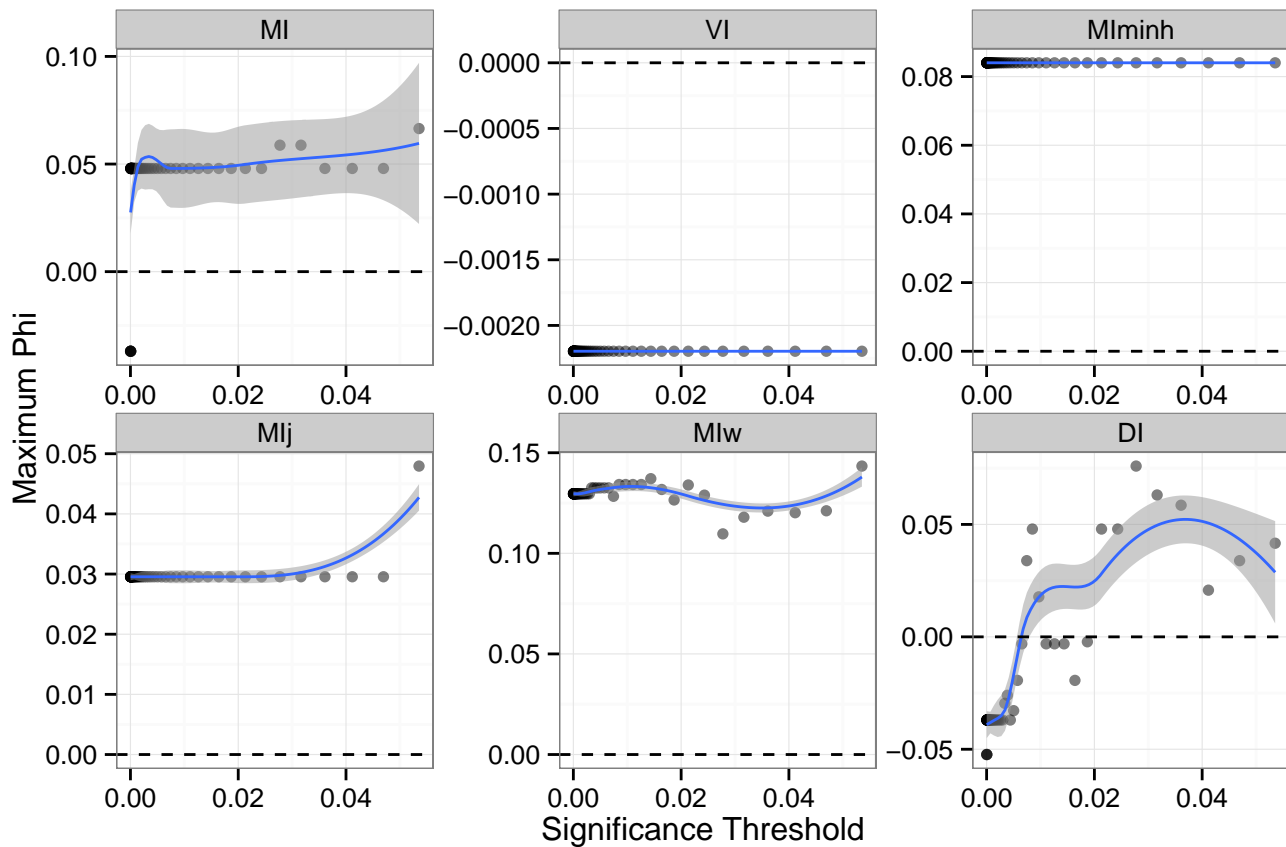

Supplement: Additional file 13 — Figure S1. HisKA-RR. Number of effective sequences (N eff) versus number of sequence (N) in the 60 sub-sampled HisKA-RR alignments. Dashed line indicates the diagonal. Blue line indicates a linear fit with 95 % confidence intervals in gray. Figure S2. Ovch32. Number of effective sequences (N eff) versus number of sequence (N) in the Ovch32 alignments. Dashed line indicates the diagonal. Blue line indicates a linear fit with 95 % confidence intervals in gray. Figure S3. Distribution of C β distances in HisKA-RR interaction (PDB: 3DGE). Figure S4. Distribution of C β distances in Ovch32 interactions [67] (See supplemental file for PDB accessions). Figure S5. Ovch32. Precision (PPV) versus Neff at FPR < 0.1 %. Blue lines indicate a loess fit to each method, 95 % confidence intervals are shown in gray. Figure S6. Ovch32. Power (TPR) versus Neff at FPR < 5 %. Blue lines indicate a loess fit to each method, 95 % confidence intervals are shown in gray. Figure S7. Ovch32. ϕ max versus Neff. Blue lines indicate a loess fit to each method, 95 % confidence intervals are shown in gray. Figure S8. HisKA-RR alt.. Power (TPR) vs Neff/L at FPR < 5 %. A stricter definition of positives, defined experimentally in [46–48] is used. Blue lines indicate a loess fit to each method, 95 % confidence intervals are shown in gray. Figure S9. HisKA-RR alt.. Power (TPR) vs Neff/L at FPR < 0.1 %. A stricter definition of positives, defined experimentally in [46–48] is used. Blue lines indicate a loess fit to each method, 95 % confidence intervals are shown in gray. Figure S10. HisKA-RR alt.. Precision (PPV) vs Neff/L at FPR < 0.1 %. A stricter definition of positives, defined experimentally in [46–48] is used. Blue lines indicate a loess fit to each method, 95 % confidence intervals are shown in gray. Figure S11. Ovch32. Power (TPR) at FPR < 5 % and Precision (PPV) at FPR < 0.1 % versus Neff/L. Blue lines indicate a loess fit to each method, 95 % confidence intervals are shown in gray. Figure S12 [file 12859_2015_677_MOESM13_ESM.zip › 12859_2015_677_add13/Fig_S37_MCC.perm.all.padj.pdf]

# HIV-hu interactors

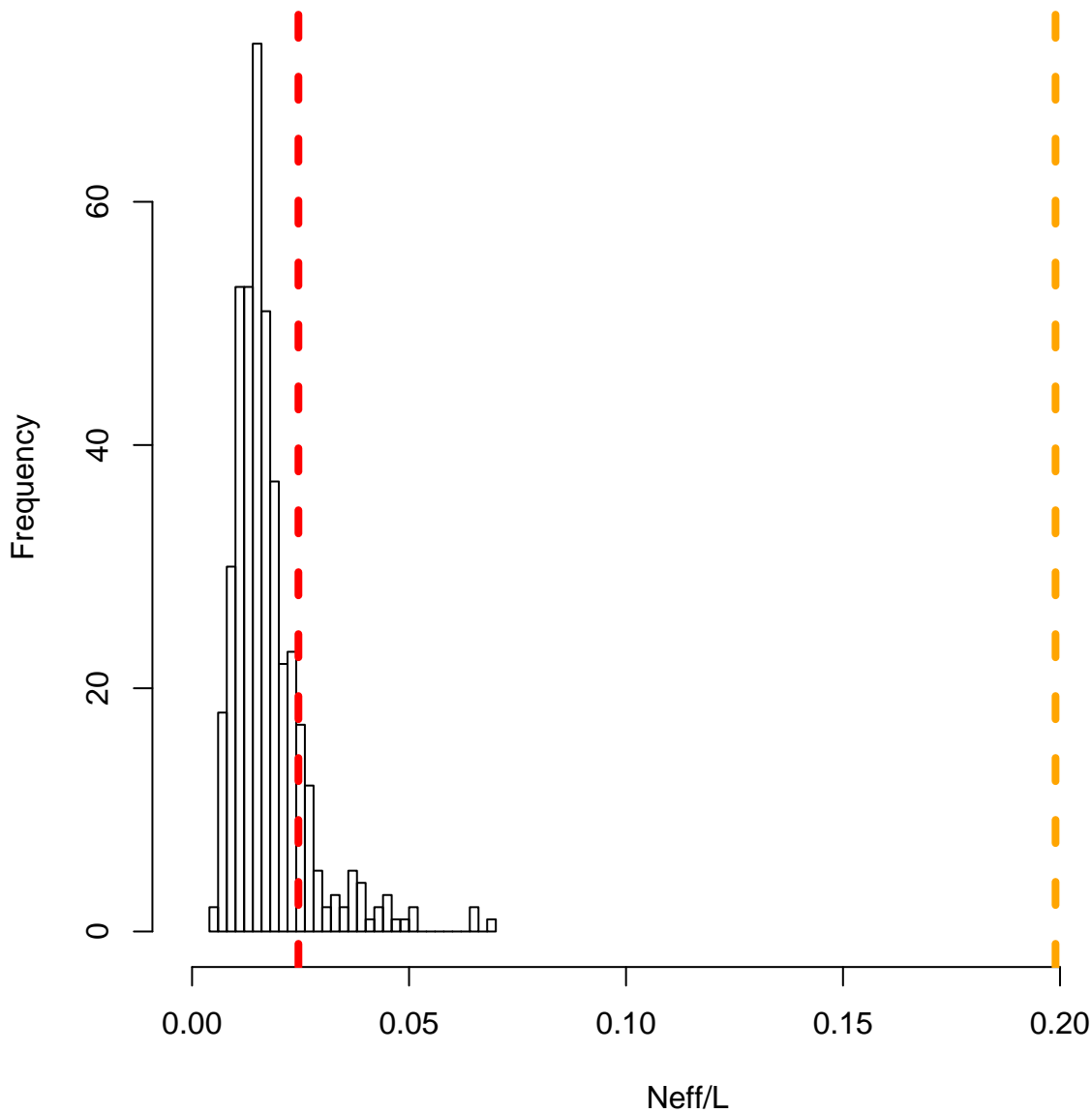

Supplement: Additional file 13 — Figure S1. HisKA-RR. Number of effective sequences (N eff) versus number of sequence (N) in the 60 sub-sampled HisKA-RR alignments. Dashed line indicates the diagonal. Blue line indicates a linear fit with 95 % confidence intervals in gray. Figure S2. Ovch32. Number of effective sequences (N eff) versus number of sequence (N) in the Ovch32 alignments. Dashed line indicates the diagonal. Blue line indicates a linear fit with 95 % confidence intervals in gray. Figure S3. Distribution of C β distances in HisKA-RR interaction (PDB: 3DGE). Figure S4. Distribution of C β distances in Ovch32 interactions [67] (See supplemental file for PDB accessions). Figure S5. Ovch32. Precision (PPV) versus Neff at FPR < 0.1 %. Blue lines indicate a loess fit to each method, 95 % confidence intervals are shown in gray. Figure S6. Ovch32. Power (TPR) versus Neff at FPR < 5 %. Blue lines indicate a loess fit to each method, 95 % confidence intervals are shown in gray. Figure S7. Ovch32. ϕ max versus Neff. Blue lines indicate a loess fit to each method, 95 % confidence intervals are shown in gray. Figure S8. HisKA-RR alt.. Power (TPR) vs Neff/L at FPR < 5 %. A stricter definition of positives, defined experimentally in [46–48] is used. Blue lines indicate a loess fit to each method, 95 % confidence intervals are shown in gray. Figure S9. HisKA-RR alt.. Power (TPR) vs Neff/L at FPR < 0.1 %. A stricter definition of positives, defined experimentally in [46–48] is used. Blue lines indicate a loess fit to each method, 95 % confidence intervals are shown in gray. Figure S10. HisKA-RR alt.. Precision (PPV) vs Neff/L at FPR < 0.1 %. A stricter definition of positives, defined experimentally in [46–48] is used. Blue lines indicate a loess fit to each method, 95 % confidence intervals are shown in gray. Figure S11. Ovch32. Power (TPR) at FPR < 5 % and Precision (PPV) at FPR < 0.1 % versus Neff/L. Blue lines indicate a loess fit to each method, 95 % confidence intervals are shown in gray. Figure S12 [file 12859_2015_677_MOESM13_ESM.zip › 12859_2015_677_add13/Fig_S38_nefflhist.pdf]

HisKA-RR Cb distances

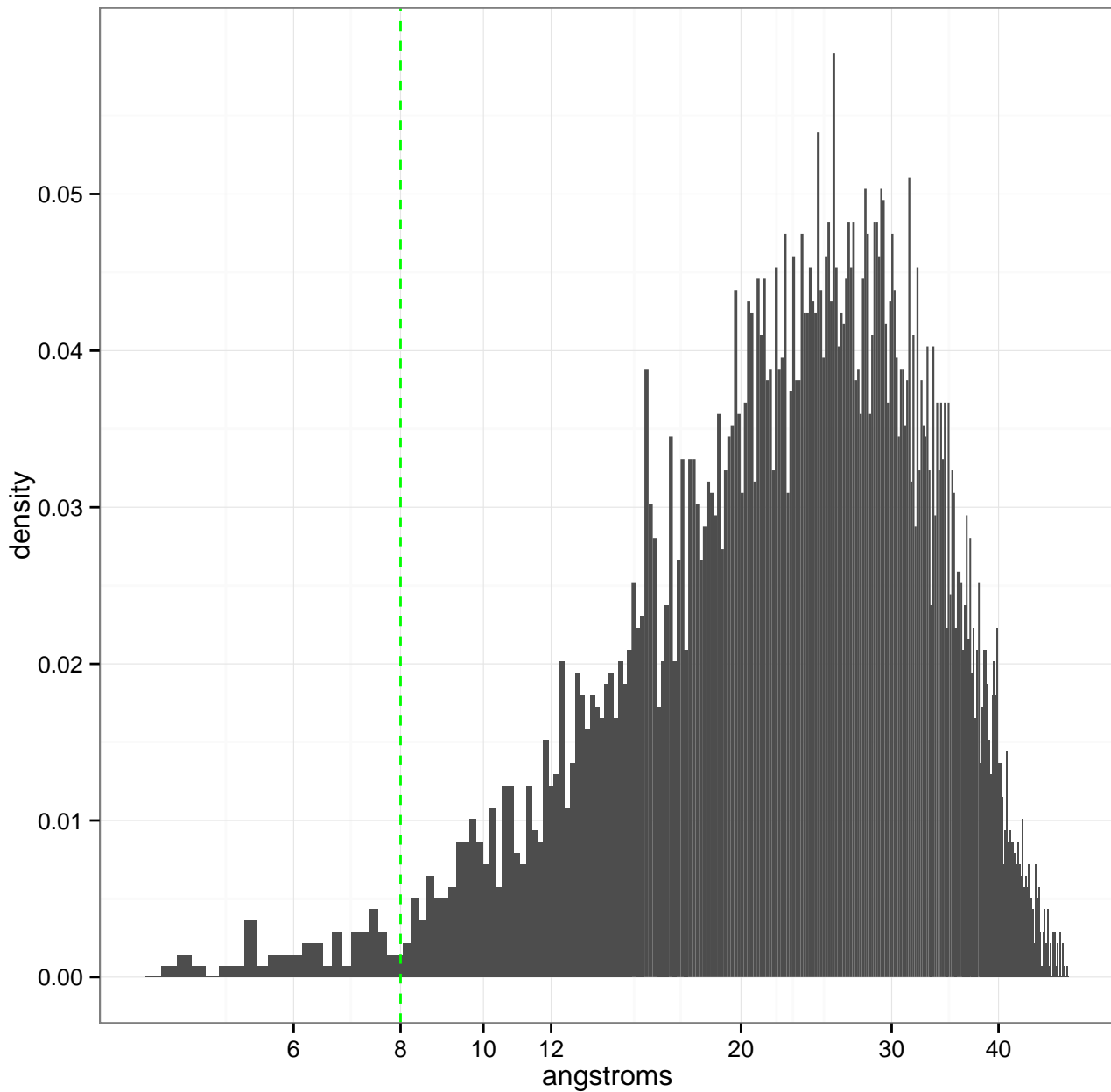

Supplement: Additional file 13 — Figure S1. HisKA-RR. Number of effective sequences (N eff) versus number of sequence (N) in the 60 sub-sampled HisKA-RR alignments. Dashed line indicates the diagonal. Blue line indicates a linear fit with 95 % confidence intervals in gray. Figure S2. Ovch32. Number of effective sequences (N eff) versus number of sequence (N) in the Ovch32 alignments. Dashed line indicates the diagonal. Blue line indicates a linear fit with 95 % confidence intervals in gray. Figure S3. Distribution of C β distances in HisKA-RR interaction (PDB: 3DGE). Figure S4. Distribution of C β distances in Ovch32 interactions [67] (See supplemental file for PDB accessions). Figure S5. Ovch32. Precision (PPV) versus Neff at FPR < 0.1 %. Blue lines indicate a loess fit to each method, 95 % confidence intervals are shown in gray. Figure S6. Ovch32. Power (TPR) versus Neff at FPR < 5 %. Blue lines indicate a loess fit to each method, 95 % confidence intervals are shown in gray. Figure S7. Ovch32. ϕ max versus Neff. Blue lines indicate a loess fit to each method, 95 % confidence intervals are shown in gray. Figure S8. HisKA-RR alt.. Power (TPR) vs Neff/L at FPR < 5 %. A stricter definition of positives, defined experimentally in [46–48] is used. Blue lines indicate a loess fit to each method, 95 % confidence intervals are shown in gray. Figure S9. HisKA-RR alt.. Power (TPR) vs Neff/L at FPR < 0.1 %. A stricter definition of positives, defined experimentally in [46–48] is used. Blue lines indicate a loess fit to each method, 95 % confidence intervals are shown in gray. Figure S10. HisKA-RR alt.. Precision (PPV) vs Neff/L at FPR < 0.1 %. A stricter definition of positives, defined experimentally in [46–48] is used. Blue lines indicate a loess fit to each method, 95 % confidence intervals are shown in gray. Figure S11. Ovch32. Power (TPR) at FPR < 5 % and Precision (PPV) at FPR < 0.1 % versus Neff/L. Blue lines indicate a loess fit to each method, 95 % confidence intervals are shown in gray. Figure S12 [file 12859_2015_677_MOESM13_ESM.zip › 12859_2015_677_add13/Fig_S3_DistanceDistribution.pdf]

Ovch32 Cb distances

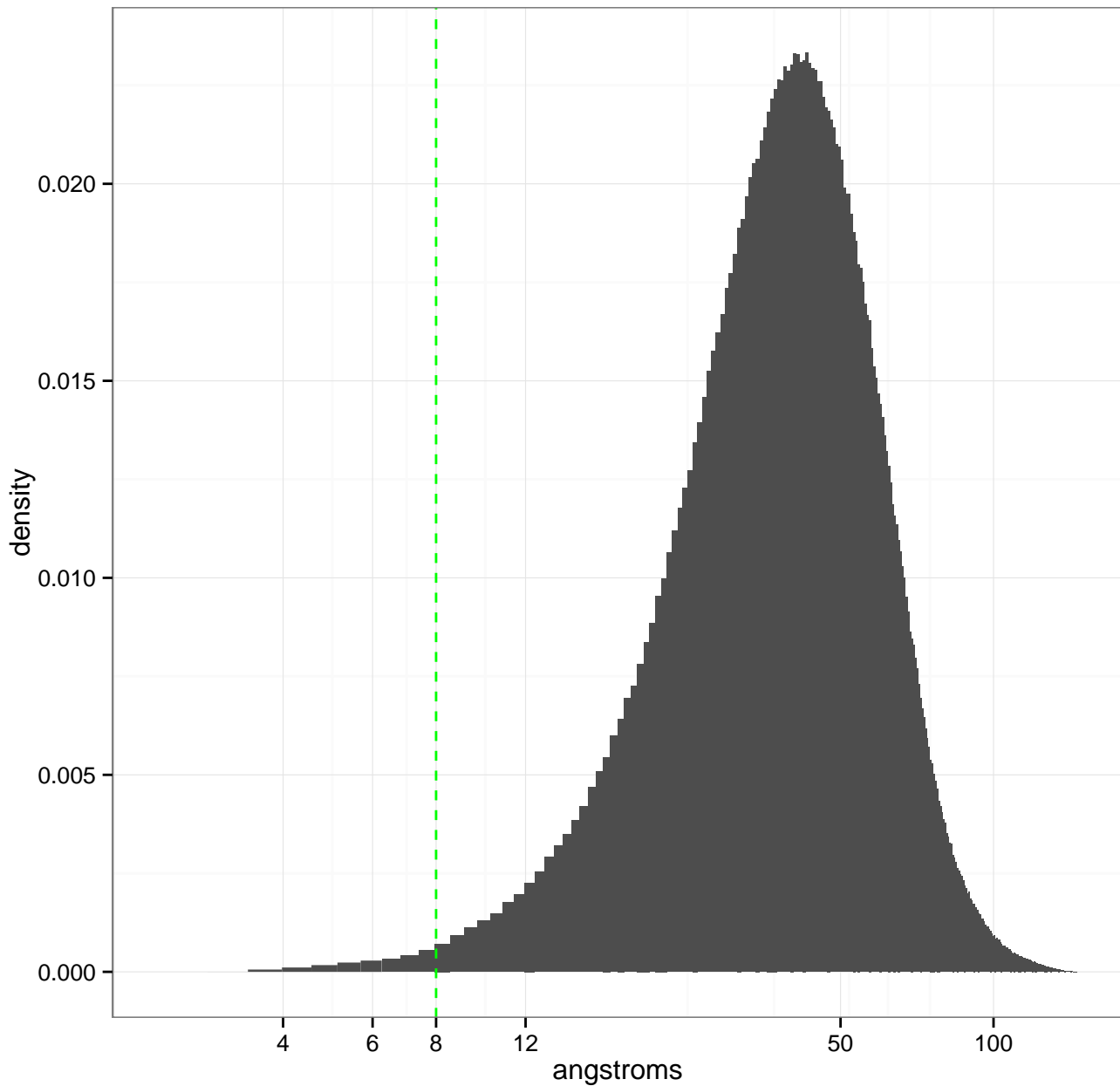

Supplement: Additional file 13 — Figure S1. HisKA-RR. Number of effective sequences (N eff) versus number of sequence (N) in the 60 sub-sampled HisKA-RR alignments. Dashed line indicates the diagonal. Blue line indicates a linear fit with 95 % confidence intervals in gray. Figure S2. Ovch32. Number of effective sequences (N eff) versus number of sequence (N) in the Ovch32 alignments. Dashed line indicates the diagonal. Blue line indicates a linear fit with 95 % confidence intervals in gray. Figure S3. Distribution of C β distances in HisKA-RR interaction (PDB: 3DGE). Figure S4. Distribution of C β distances in Ovch32 interactions [67] (See supplemental file for PDB accessions). Figure S5. Ovch32. Precision (PPV) versus Neff at FPR < 0.1 %. Blue lines indicate a loess fit to each method, 95 % confidence intervals are shown in gray. Figure S6. Ovch32. Power (TPR) versus Neff at FPR < 5 %. Blue lines indicate a loess fit to each method, 95 % confidence intervals are shown in gray. Figure S7. Ovch32. ϕ max versus Neff. Blue lines indicate a loess fit to each method, 95 % confidence intervals are shown in gray. Figure S8. HisKA-RR alt.. Power (TPR) vs Neff/L at FPR < 5 %. A stricter definition of positives, defined experimentally in [46–48] is used. Blue lines indicate a loess fit to each method, 95 % confidence intervals are shown in gray. Figure S9. HisKA-RR alt.. Power (TPR) vs Neff/L at FPR < 0.1 %. A stricter definition of positives, defined experimentally in [46–48] is used. Blue lines indicate a loess fit to each method, 95 % confidence intervals are shown in gray. Figure S10. HisKA-RR alt.. Precision (PPV) vs Neff/L at FPR < 0.1 %. A stricter definition of positives, defined experimentally in [46–48] is used. Blue lines indicate a loess fit to each method, 95 % confidence intervals are shown in gray. Figure S11. Ovch32. Power (TPR) at FPR < 5 % and Precision (PPV) at FPR < 0.1 % versus Neff/L. Blue lines indicate a loess fit to each method, 95 % confidence intervals are shown in gray. Figure S12 [file 12859_2015_677_MOESM13_ESM.zip › 12859_2015_677_add13/Fig_S4_DistanceDistribution.pdf]

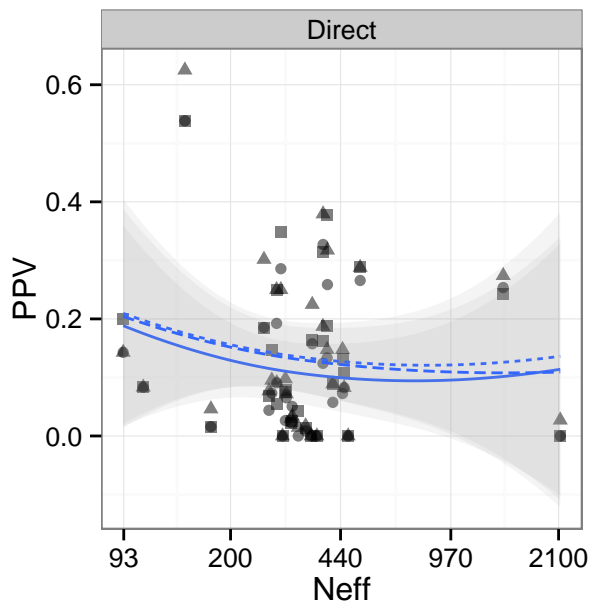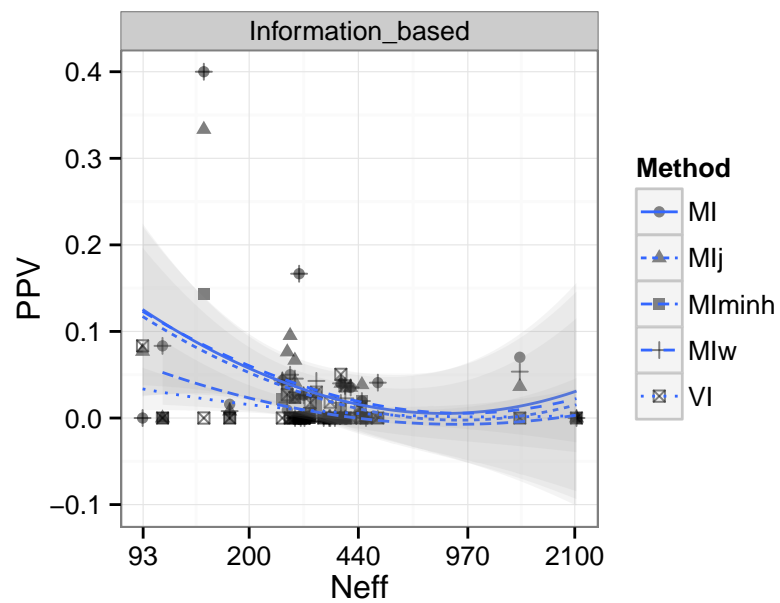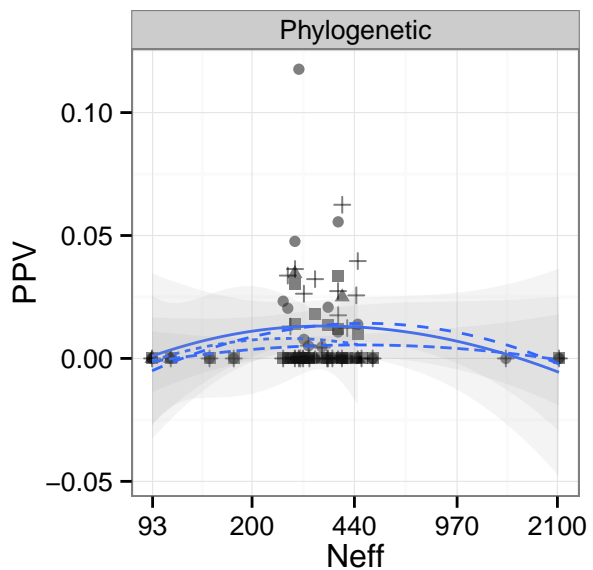

Supplement: Additional file 13 — Figure S1. HisKA-RR. Number of effective sequences (N eff) versus number of sequence (N) in the 60 sub-sampled HisKA-RR alignments. Dashed line indicates the diagonal. Blue line indicates a linear fit with 95 % confidence intervals in gray. Figure S2. Ovch32. Number of effective sequences (N eff) versus number of sequence (N) in the Ovch32 alignments. Dashed line indicates the diagonal. Blue line indicates a linear fit with 95 % confidence intervals in gray. Figure S3. Distribution of C β distances in HisKA-RR interaction (PDB: 3DGE). Figure S4. Distribution of C β distances in Ovch32 interactions [67] (See supplemental file for PDB accessions). Figure S5. Ovch32. Precision (PPV) versus Neff at FPR < 0.1 %. Blue lines indicate a loess fit to each method, 95 % confidence intervals are shown in gray. Figure S6. Ovch32. Power (TPR) versus Neff at FPR < 5 %. Blue lines indicate a loess fit to each method, 95 % confidence intervals are shown in gray. Figure S7. Ovch32. ϕ max versus Neff. Blue lines indicate a loess fit to each method, 95 % confidence intervals are shown in gray. Figure S8. HisKA-RR alt.. Power (TPR) vs Neff/L at FPR < 5 %. A stricter definition of positives, defined experimentally in [46–48] is used. Blue lines indicate a loess fit to each method, 95 % confidence intervals are shown in gray. Figure S9. HisKA-RR alt.. Power (TPR) vs Neff/L at FPR < 0.1 %. A stricter definition of positives, defined experimentally in [46–48] is used. Blue lines indicate a loess fit to each method, 95 % confidence intervals are shown in gray. Figure S10. HisKA-RR alt.. Precision (PPV) vs Neff/L at FPR < 0.1 %. A stricter definition of positives, defined experimentally in [46–48] is used. Blue lines indicate a loess fit to each method, 95 % confidence intervals are shown in gray. Figure S11. Ovch32. Power (TPR) at FPR < 5 % and Precision (PPV) at FPR < 0.1 % versus Neff/L. Blue lines indicate a loess fit to each method, 95 % confidence intervals are shown in gray. Figure S12 [file 12859_2015_677_MOESM13_ESM.zip › 12859_2015_677_add13/Fig_S5_PPV_Neff_at_0.001.pdf]

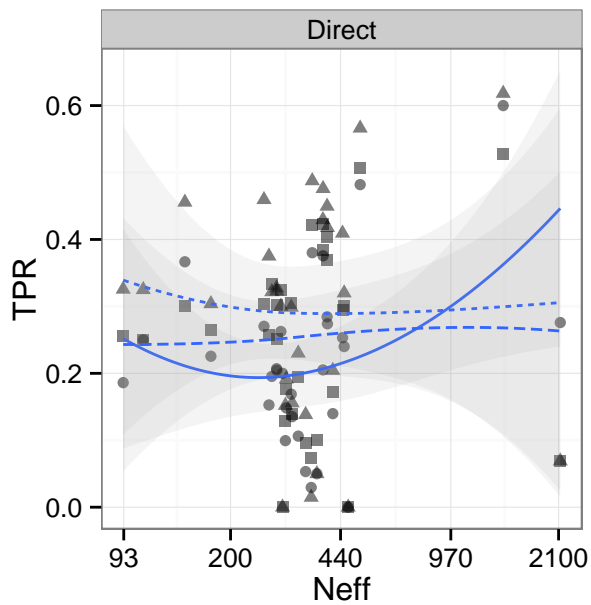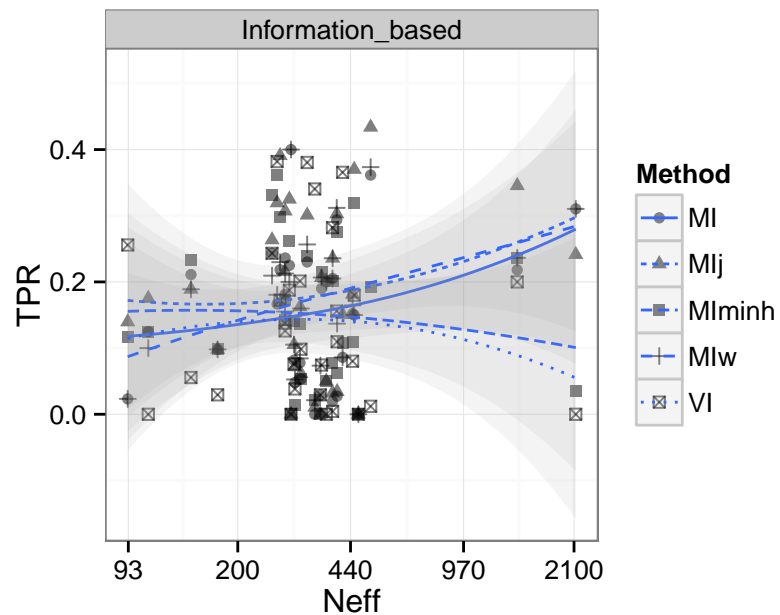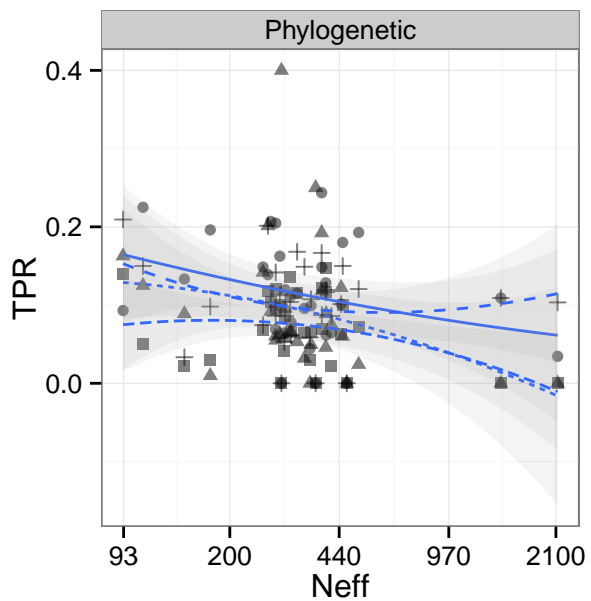

Supplement: Additional file 13 — Figure S1. HisKA-RR. Number of effective sequences (N eff) versus number of sequence (N) in the 60 sub-sampled HisKA-RR alignments. Dashed line indicates the diagonal. Blue line indicates a linear fit with 95 % confidence intervals in gray. Figure S2. Ovch32. Number of effective sequences (N eff) versus number of sequence (N) in the Ovch32 alignments. Dashed line indicates the diagonal. Blue line indicates a linear fit with 95 % confidence intervals in gray. Figure S3. Distribution of C β distances in HisKA-RR interaction (PDB: 3DGE). Figure S4. Distribution of C β distances in Ovch32 interactions [67] (See supplemental file for PDB accessions). Figure S5. Ovch32. Precision (PPV) versus Neff at FPR < 0.1 %. Blue lines indicate a loess fit to each method, 95 % confidence intervals are shown in gray. Figure S6. Ovch32. Power (TPR) versus Neff at FPR < 5 %. Blue lines indicate a loess fit to each method, 95 % confidence intervals are shown in gray. Figure S7. Ovch32. ϕ max versus Neff. Blue lines indicate a loess fit to each method, 95 % confidence intervals are shown in gray. Figure S8. HisKA-RR alt.. Power (TPR) vs Neff/L at FPR < 5 %. A stricter definition of positives, defined experimentally in [46–48] is used. Blue lines indicate a loess fit to each method, 95 % confidence intervals are shown in gray. Figure S9. HisKA-RR alt.. Power (TPR) vs Neff/L at FPR < 0.1 %. A stricter definition of positives, defined experimentally in [46–48] is used. Blue lines indicate a loess fit to each method, 95 % confidence intervals are shown in gray. Figure S10. HisKA-RR alt.. Precision (PPV) vs Neff/L at FPR < 0.1 %. A stricter definition of positives, defined experimentally in [46–48] is used. Blue lines indicate a loess fit to each method, 95 % confidence intervals are shown in gray. Figure S11. Ovch32. Power (TPR) at FPR < 5 % and Precision (PPV) at FPR < 0.1 % versus Neff/L. Blue lines indicate a loess fit to each method, 95 % confidence intervals are shown in gray. Figure S12 [file 12859_2015_677_MOESM13_ESM.zip › 12859_2015_677_add13/Fig_S6_TPR_Neff_at_0.05.pdf]

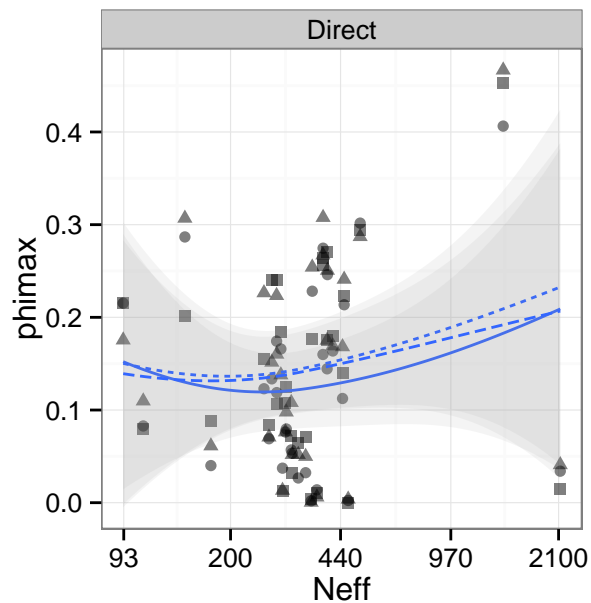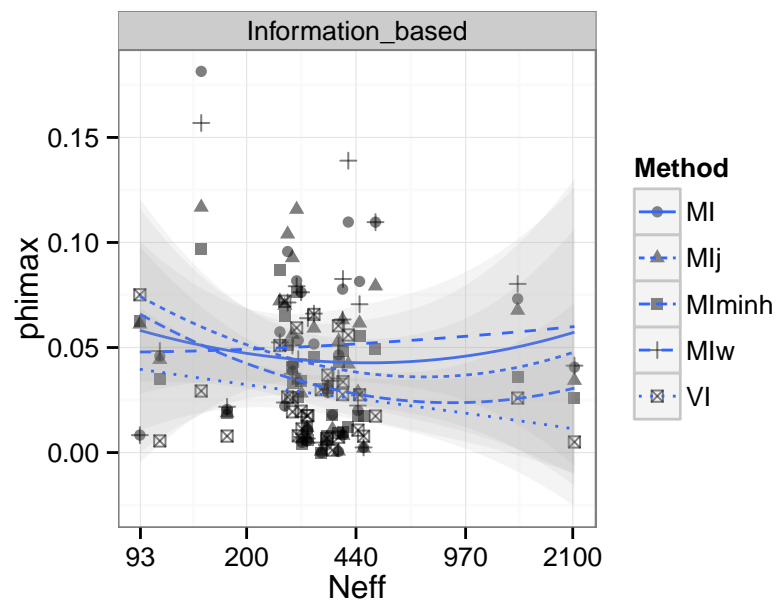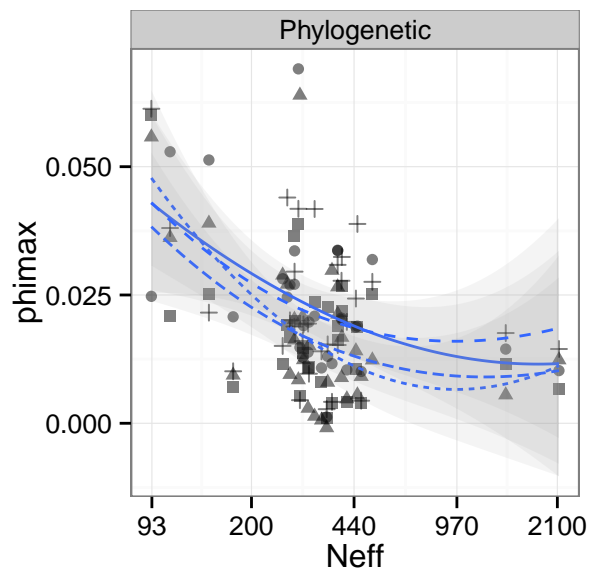

Supplement: Additional file 13 — Figure S1. HisKA-RR. Number of effective sequences (N eff) versus number of sequence (N) in the 60 sub-sampled HisKA-RR alignments. Dashed line indicates the diagonal. Blue line indicates a linear fit with 95 % confidence intervals in gray. Figure S2. Ovch32. Number of effective sequences (N eff) versus number of sequence (N) in the Ovch32 alignments. Dashed line indicates the diagonal. Blue line indicates a linear fit with 95 % confidence intervals in gray. Figure S3. Distribution of C β distances in HisKA-RR interaction (PDB: 3DGE). Figure S4. Distribution of C β distances in Ovch32 interactions [67] (See supplemental file for PDB accessions). Figure S5. Ovch32. Precision (PPV) versus Neff at FPR < 0.1 %. Blue lines indicate a loess fit to each method, 95 % confidence intervals are shown in gray. Figure S6. Ovch32. Power (TPR) versus Neff at FPR < 5 %. Blue lines indicate a loess fit to each method, 95 % confidence intervals are shown in gray. Figure S7. Ovch32. ϕ max versus Neff. Blue lines indicate a loess fit to each method, 95 % confidence intervals are shown in gray. Figure S8. HisKA-RR alt.. Power (TPR) vs Neff/L at FPR < 5 %. A stricter definition of positives, defined experimentally in [46–48] is used. Blue lines indicate a loess fit to each method, 95 % confidence intervals are shown in gray. Figure S9. HisKA-RR alt.. Power (TPR) vs Neff/L at FPR < 0.1 %. A stricter definition of positives, defined experimentally in [46–48] is used. Blue lines indicate a loess fit to each method, 95 % confidence intervals are shown in gray. Figure S10. HisKA-RR alt.. Precision (PPV) vs Neff/L at FPR < 0.1 %. A stricter definition of positives, defined experimentally in [46–48] is used. Blue lines indicate a loess fit to each method, 95 % confidence intervals are shown in gray. Figure S11. Ovch32. Power (TPR) at FPR < 5 % and Precision (PPV) at FPR < 0.1 % versus Neff/L. Blue lines indicate a loess fit to each method, 95 % confidence intervals are shown in gray. Figure S12 [file 12859_2015_677_MOESM13_ESM.zip › 12859_2015_677_add13/Fig_S7_phimax_Neff_at_NA.pdf]

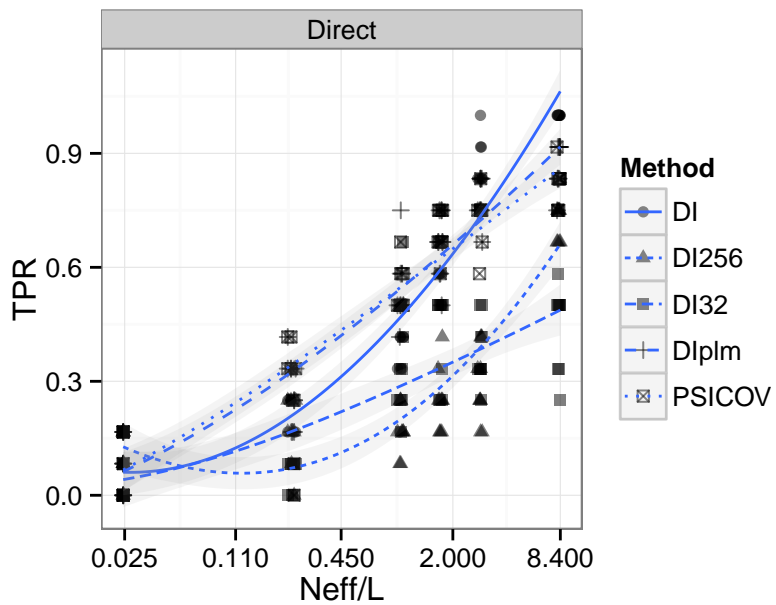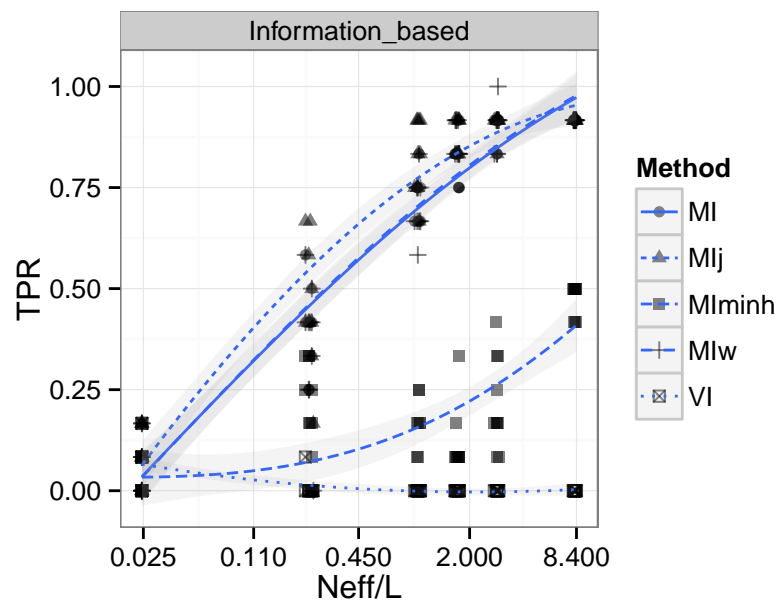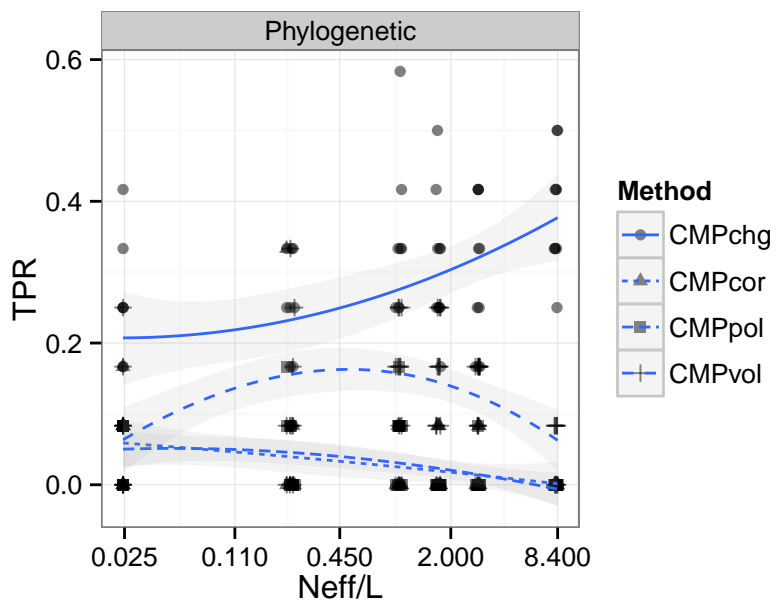

Supplement: Additional file 13 — Figure S1. HisKA-RR. Number of effective sequences (N eff) versus number of sequence (N) in the 60 sub-sampled HisKA-RR alignments. Dashed line indicates the diagonal. Blue line indicates a linear fit with 95 % confidence intervals in gray. Figure S2. Ovch32. Number of effective sequences (N eff) versus number of sequence (N) in the Ovch32 alignments. Dashed line indicates the diagonal. Blue line indicates a linear fit with 95 % confidence intervals in gray. Figure S3. Distribution of C β distances in HisKA-RR interaction (PDB: 3DGE). Figure S4. Distribution of C β distances in Ovch32 interactions [67] (See supplemental file for PDB accessions). Figure S5. Ovch32. Precision (PPV) versus Neff at FPR < 0.1 %. Blue lines indicate a loess fit to each method, 95 % confidence intervals are shown in gray. Figure S6. Ovch32. Power (TPR) versus Neff at FPR < 5 %. Blue lines indicate a loess fit to each method, 95 % confidence intervals are shown in gray. Figure S7. Ovch32. ϕ max versus Neff. Blue lines indicate a loess fit to each method, 95 % confidence intervals are shown in gray. Figure S8. HisKA-RR alt.. Power (TPR) vs Neff/L at FPR < 5 %. A stricter definition of positives, defined experimentally in [46–48] is used. Blue lines indicate a loess fit to each method, 95 % confidence intervals are shown in gray. Figure S9. HisKA-RR alt.. Power (TPR) vs Neff/L at FPR < 0.1 %. A stricter definition of positives, defined experimentally in [46–48] is used. Blue lines indicate a loess fit to each method, 95 % confidence intervals are shown in gray. Figure S10. HisKA-RR alt.. Precision (PPV) vs Neff/L at FPR < 0.1 %. A stricter definition of positives, defined experimentally in [46–48] is used. Blue lines indicate a loess fit to each method, 95 % confidence intervals are shown in gray. Figure S11. Ovch32. Power (TPR) at FPR < 5 % and Precision (PPV) at FPR < 0.1 % versus Neff/L. Blue lines indicate a loess fit to each method, 95 % confidence intervals are shown in gray. Figure S12 [file 12859_2015_677_MOESM13_ESM.zip › 12859_2015_677_add13/Fig_S8_TPR_Neff_per_col_at_0.05.pdf]

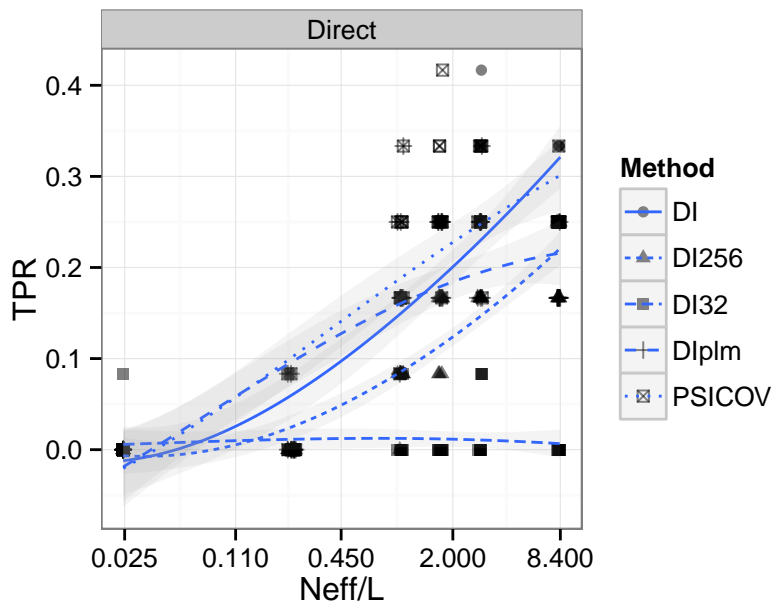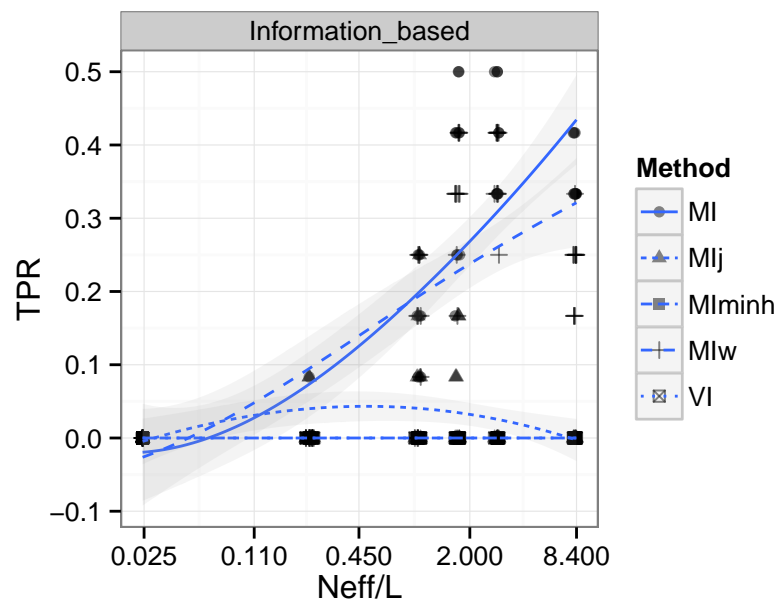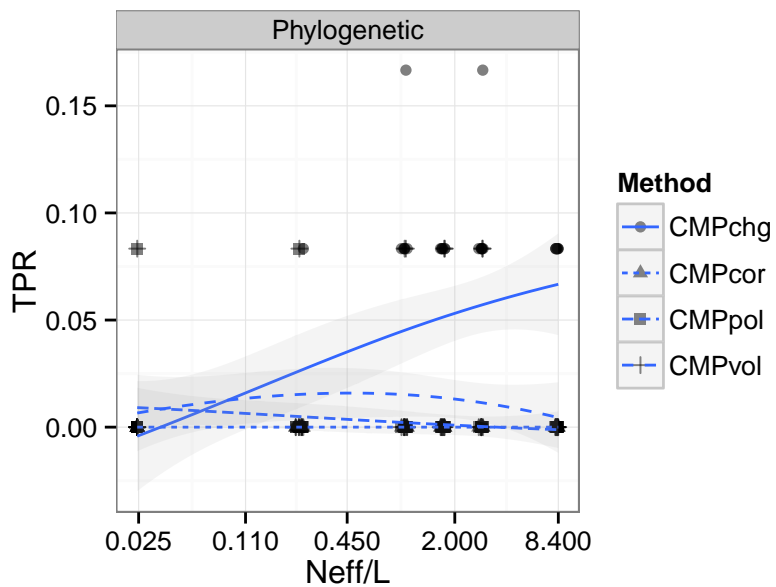

Supplement: Additional file 13 — Figure S1. HisKA-RR. Number of effective sequences (N eff) versus number of sequence (N) in the 60 sub-sampled HisKA-RR alignments. Dashed line indicates the diagonal. Blue line indicates a linear fit with 95 % confidence intervals in gray. Figure S2. Ovch32. Number of effective sequences (N eff) versus number of sequence (N) in the Ovch32 alignments. Dashed line indicates the diagonal. Blue line indicates a linear fit with 95 % confidence intervals in gray. Figure S3. Distribution of C β distances in HisKA-RR interaction (PDB: 3DGE). Figure S4. Distribution of C β distances in Ovch32 interactions [67] (See supplemental file for PDB accessions). Figure S5. Ovch32. Precision (PPV) versus Neff at FPR < 0.1 %. Blue lines indicate a loess fit to each method, 95 % confidence intervals are shown in gray. Figure S6. Ovch32. Power (TPR) versus Neff at FPR < 5 %. Blue lines indicate a loess fit to each method, 95 % confidence intervals are shown in gray. Figure S7. Ovch32. ϕ max versus Neff. Blue lines indicate a loess fit to each method, 95 % confidence intervals are shown in gray. Figure S8. HisKA-RR alt.. Power (TPR) vs Neff/L at FPR < 5 %. A stricter definition of positives, defined experimentally in [46–48] is used. Blue lines indicate a loess fit to each method, 95 % confidence intervals are shown in gray. Figure S9. HisKA-RR alt.. Power (TPR) vs Neff/L at FPR < 0.1 %. A stricter definition of positives, defined experimentally in [46–48] is used. Blue lines indicate a loess fit to each method, 95 % confidence intervals are shown in gray. Figure S10. HisKA-RR alt.. Precision (PPV) vs Neff/L at FPR < 0.1 %. A stricter definition of positives, defined experimentally in [46–48] is used. Blue lines indicate a loess fit to each method, 95 % confidence intervals are shown in gray. Figure S11. Ovch32. Power (TPR) at FPR < 5 % and Precision (PPV) at FPR < 0.1 % versus Neff/L. Blue lines indicate a loess fit to each method, 95 % confidence intervals are shown in gray. Figure S12 [file 12859_2015_677_MOESM13_ESM.zip › 12859_2015_677_add13/Fig_S9_TPR_Neff_per_col_at_0.001.pdf]
